# Synthesis and Characterization of Folic Acid-Conjugated Terbium Complexes as Luminescent Probes for Targeting Folate Receptor-Expressing Cells

**DOI:** 10.1021/acs.jmedchem.4c00919

**Published:** 2024-08-14

**Authors:** Grace T. McMullon, Aiarpi Ezdoglian, Anna C. Booth, Pilar Jimenez-Royo, Philip S. Murphy, Gerrit Jansen, Conny J. van der Laken, Stephen Faulkner

**Affiliations:** † Chemistry Research Laboratory, University of Oxford, Oxford OX1 3TA, United Kingdom; ‡ Department of Rheumatology and Clinical Immunology, Amsterdam University Medical Center, Location VU University Medical Center, 1081 HV Amsterdam, Netherlands; § GlaxoSmithKline, Gunnels Wood Road, Stevenage, Hertfordshire SG1 2NY, United Kingdom

## Abstract

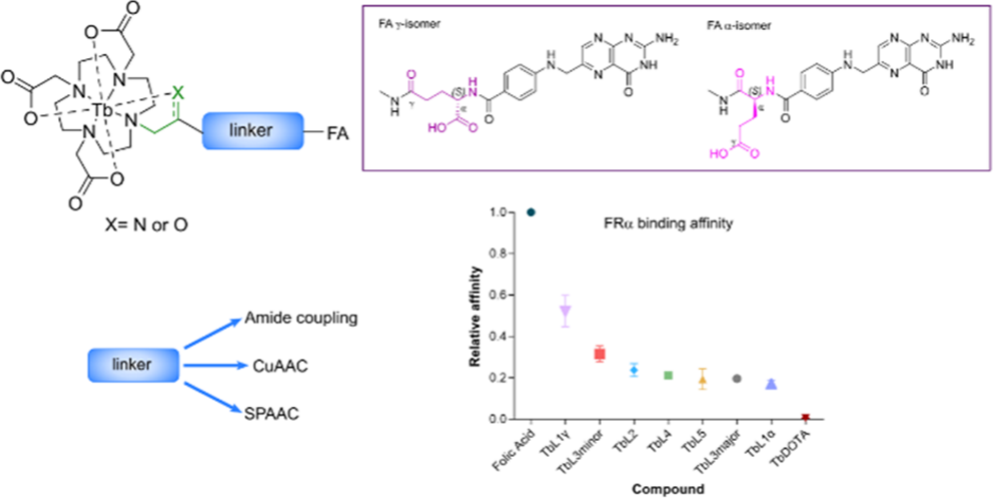

Several conjugates between folic acid and a series of kinetically stable lanthanide complexes have been synthesized, using amide coupling and azide–alkyne cycloaddition methodologies to link the metal-binding domain to folate through a variety of spacer groups. While all these complexes exhibit affinity for the folate receptor, it is clear that the point of attachment to folate is essential, with linkage through the γ-carboxylic acid giving rise to significantly enhanced receptor affinity. All the conjugates studied show affinities consistent with displacing biological circulating folate derivatives, 5-methyltetrahydrofolate, from folate receptors. All the complexes exhibit luminescence with a short-lived component arising from ligand fluorescence overlaid on a much longer lived terbium-centered component. These can be separated using time-gating methods. From the results obtained, the most promising approach to achieve sensitized luminescence in these systems requires incorporating a sensitizing chromophore close to the lanthanide.

## 1 Introduction

Chronic inflammation is prevalent in society with autoimmune diseases such as rheumatoid arthritis (RA), asthma, and Crohn’s disease becoming increasingly common. Still, it can also appear as part of other diseases such as COVID-19 infection, and host against transplant rejection. Because of this, there is a great need to develop imaging agents that help selectively visualize inflammatory lesions *in vivo* to support early diagnosis and development of a personalized treatment approach. Macrophages play a crucial orchestrating role in the development and maintenance of chronic inflammation; therefore, these cells are promising targets for imaging inflammation.^1,2^ Folate receptor β (FRβ) is selectively overexpressed in activated macrophages and this overexpression has demonstrated a robust correlation with inflammation levels in several diseases such as RA and osteoarthritis.^3−6^

While macrophages utilize FRβ during activation, rapidly dividing cancer cells require more folate for DNA synthesis. Therefore, folate receptors are often overexpressed on cancer cells, supporting increased folate uptake.^7−9^ Folate receptor α (FRα) rather than FRβ is overexpressed in various cancers such as ovarian, lung, and breast cancers^10^ and is used for both imaging and therapeutic purposes.^11−15^

To this end, using FRα and FRβ in imaging improves the management and treatment of a spectrum of conditions characterized by chronic inflammation and uncontrolled cellular proliferation.

Human folate receptors have a high binding affinity to folic acid and antifolates,^4^ and as such there has been great interest in the development of probes based on their structure.^16,17^ In fact, the antifolate drug methotrexate is an anchor drug in several chronic rheumatic diseases including RA.^18^

Folic acid consists of three distinguishable fragments: pterin, 4-aminobenzoic acid, and glutamic acid, the latter of which has two potential sites for conjugation (α- and γ- carboxylates). The crystal structures of FRα^19^ and FRβ^20^ complexed with folic acid have been reported, helping to elucidate the important structural features of the folate-derivates within the FR binding pocket.^19,20^ The pteroic acid binds furthest within the FR binding cavity, and the terminal glutamic acid resides closest to the entrance of the receptor cavity with the glutamic acid carboxylates forming hydrogen bonds and charge–charge interactions.^20^ These structures support the functionalization of the γ-carboxyl moiety of glutamic acid,^20^ which is the most prolific position for attachment of an imaging or therapeutic group reported in the literature.^13,21−25^ Nevertheless, positions of functionalization remain widely debated among the community, with some reporting little to no difference between the α- and γ-regioisomers,^16,26^ in some cases leading to the use of a mixture of the regioisomers.^17,27^ In this work, one of our aims was to evaluate the importance of the site of glutamic acid attachment.

Compounds targeting the FRα and FRβ have previously been reported as imaging agents in magnetic resonance imaging (MRI),^23,24,28^ positron emission tomography (PET),^13,15^ optical^25,27,29^ and single-photon emission computed tomography (SPECT).^6,30^ Optical imaging (OI) is a highly sensitive, noninvasive diagnostic technique that is used to visualize disease-specific biomarkers both *in vitro* and *in vivo*. Luminescence microscopy offers submicrometer spatial resolution and high sensitivity, which is especially useful in investigating systems at a cellular level often used in preclinical models in early development. In the oncology field, OI agents are used for immediate visualization over short distances, for example during endoscopy, enabling interventions with precision. OTL38 is the first FDA-approved folic acid near-infrared dye for the detection of ovarian and lung cancers for use in fluorescence-guided surgery.^31−34^ This compound significantly improves tumor identification and enables more precise tissue resection due to the high spatial resolution. In rheumatology OI is superior in small joint imaging for fine tissue margins. OI also reduces the radiation exposure compared to PET imaging. However, OI cannot be used for deeper anatomical structures and in some cases the natural tissue autofluorescence can affect the quality of the tissue detection: in these cases PET imaging would be the preferred modality. Additionally, specialized equipment is required for OI utilization. Thus, while both PET and OI are useful diagnostic tools, they have distinct selection pros and cons.

Lanthanide complexes have been used for cellular imaging due to their desirable properties: characteristic narrow, distinctive emission peaks and long-lived luminescence.^35^ These long lanthanide emission lifetimes enable time-resolved image acquisition to distinguish from short-lived autofluorescence - this leads to improved signal-to-noise ratio and therefore more precise visualization.^36,37^ Furthermore, emerging lanthanide radiochemistry with ^161^Tb, will mean that this is directly transferrable to PET imaging applications. ^161^Tb and ^177^Lu offer potential for translation of this work to radioconjugates with folate targeting.^38,39^

**Chart 1:**
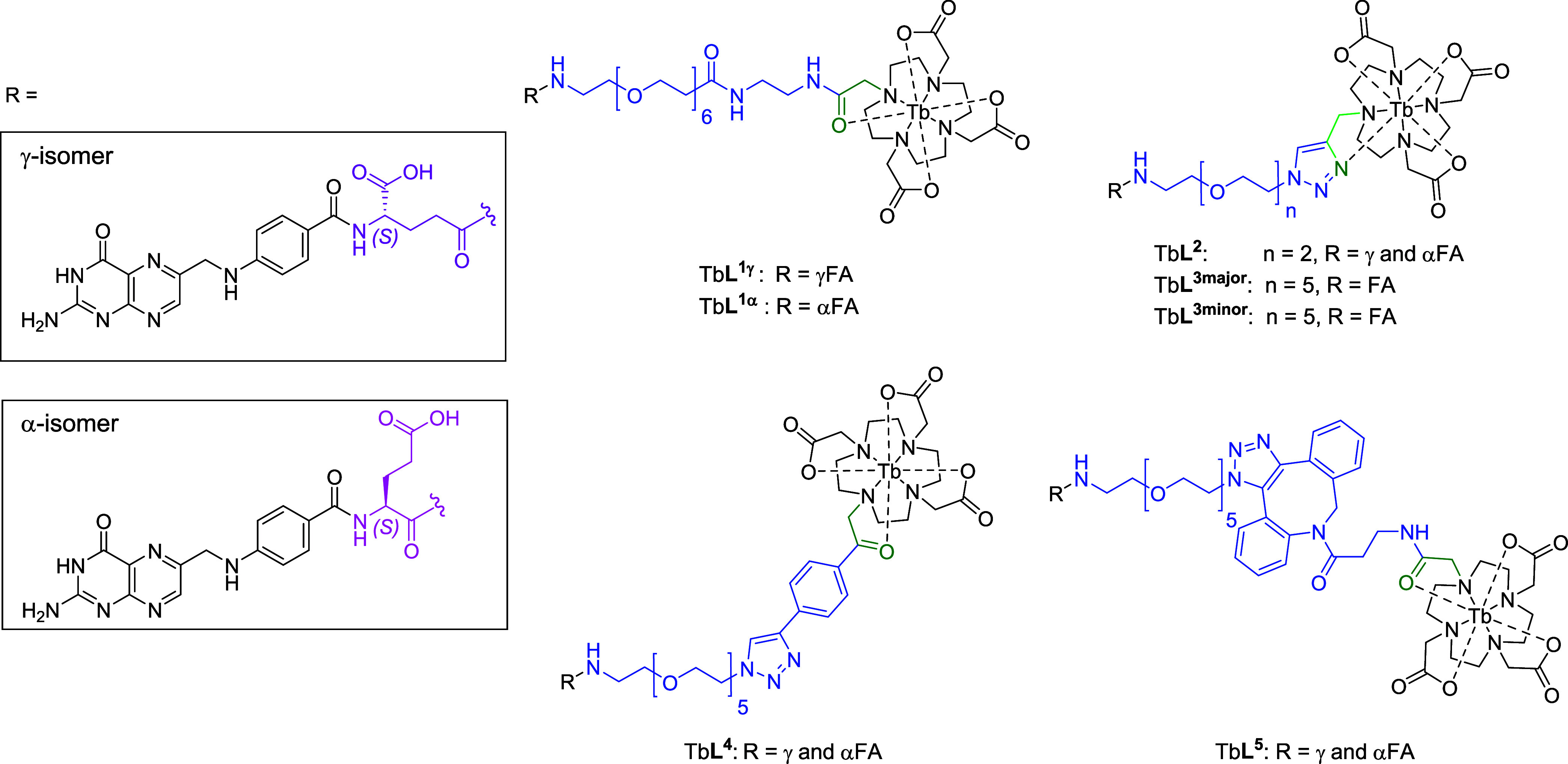
Tb-folate Conjugates, TbL^1^-L^5^a

This work describes the synthesis and characterization of a series of first-generation folic acid terbium conjugate imaging probes (Tb**L**^**1**–**5**^) for FR^+^ cell imaging (Chart 1). The basic structure is that of a bifunctional chelating agent that consists of a terbium chelate connected to a folate moiety via a linker of varying lengths and bioconjugation strategies. Terbium was selected as it is more emissive than europium, because of the larger energy gap between their main emissive and ground states.^40^ This work explores different conjugation techniques for attaching the folate-targeting moiety including amide bond formation and the formation of triazoles by azide–alkyne cycloaddition. Both copper-catalyzed and strain-promoted triazole cycloadditions are studied using the previously reported pDO3A (propargyl 1,4,7,10-tetraazacyclododecane-1,4,7-trisacetic acid) for CuAAC (copper-catalyzed azide–alkyne cycloaddition)^41^ and DOTA-DBCO (dibenzocyclooctyne-1,4,7,10-tetraazacyclododecane-1,4,7,10-tetraacetic acid) for SPAAC (strain-promoted azide–alkyne cycloaddition),^42,43^ as well as an unreported macrocyclic DO3A with a 4′-ethynylacetophenone pendant arm for functionalization. Moreover, different linker lengths were incorporated to determine the influence on probe emission and cellular FR binding properties.

a Dark green highlights carboxyl- oxygen coordination, light green highlights azide-nitrogen coordination, dark purple highlights folic acid as the γ-regioisomer, and magenta highlights folic acid as the α-regioisomer, blue highlights the different linkers.

## 2 Results and Discussion

### 2.1 Synthesis of Complexes

For bioconjugation, to obtain the separate regioisomers of the folate-PEG_6_ ligand, **S2**^**α/γ**^ the molecule was prepared by stepwise solid-phase synthesis adapted from literature and purified by RP-HPLC in low yields (γ = 19%, α = 5%).^22,44^ Folate-PEG_6_**S2**^**α/γ**^ was then coupled to DOTA-NHS ester, followed by metal complexation with terbium trifluoromethane sulfonate in water at pH 4 (Scheme S1). Tb**L**^**1γ**^ and Tb**L**^**1α**^ were obtained separately as yellow powders after purification by RP-HPLC and lypophilization.^22^

**Scheme 1:**
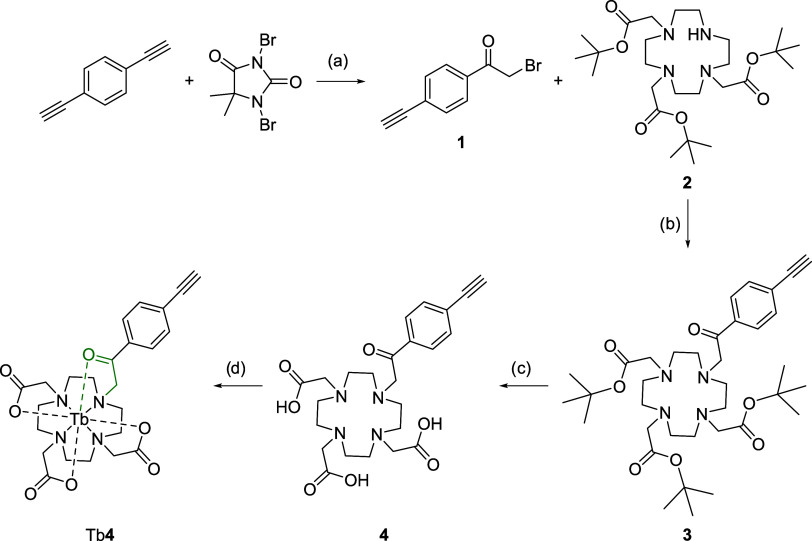
Synthesis of Tb-ethynylacetophenoneDO3A, Tb4 Reagents and conditions: (a) ethylene thiourea, acetone:H_2_O (100:1), 45 °C, 4 h, 19%; (b) K_2_CO_3_, MeCN, 20 °C → 60 °C, 16 h, 35%; (c) TFA/DCM (1:1), 20 °C, 16–24 h, 90%; (d) Tb(OTf)_3_, MeOH, pH 5, 60 °C, 48 h.

For bioorthogonal azide–alkyne cycloaddition, the previously reported alkynes, pDO3A, **7** and DOTA-DBCO, **S6**, were prepared according to modified literature procedures.^41−43^ The previously unreported 4′-ethynyl-2-acetophenone-DO3A, **4** was prepared as outlined in Scheme 1. Briefly, the 4′-ethynyl-2-bromoacetophenone, **1** was prepared by reacting 1,4-diethynylbenzene and 1,3-dibromo-5,5-dimethylhydantoin in the presence of *N,N*′-ethylenethiourea as catalyst in aqueous acetone at 45 °C and then purified by column chromatography to afford the product, **1** as a yellow oil. 4′-Ethynyl-2-bromoacetophenone, **1** was then reacted with *tert*-butyl protected DO3A, **2** to yield **3**. Subsequent deprotection with 1:1 trifluoroacetic acid and dichloromethane yielded **4**. Metal complexation was carried out with terbium trifluoromethane sulfonate to produce Tb**4**.

**Scheme 2:**
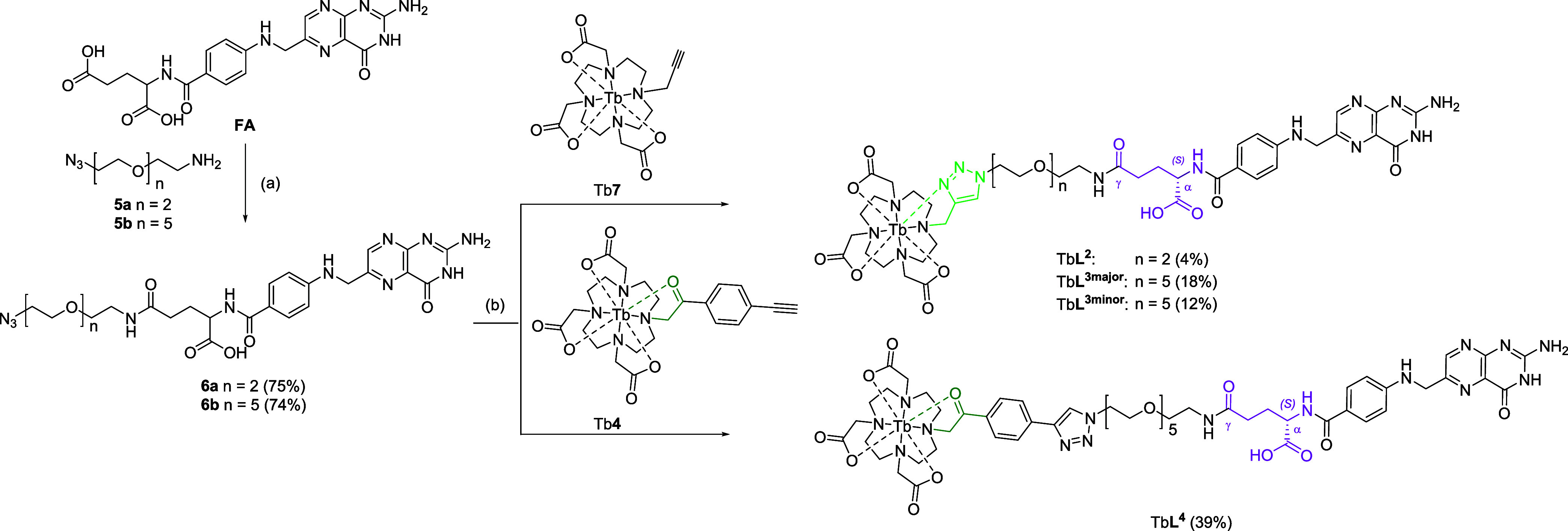
Synthesis of TbL^2^-TbL^4^ using CuAAC Conditions Reagents and conditions: (a) DCC (2.0 equiv), pyridine, DMSO, 20 °C, 60 h, dark; (b) (CuOTf)_2_·C_6_H_6_ (0.1 equiv) or CuSO_4_·5H_2_O (0.1 equiv) and sodium ascorbate (0.5 equiv), Ar, 40–80 °C, MeOH/DMSO/H_2_O, 24–48 h. Only α-isomer is shown.

Azido folate, **6**, was prepared using a modified literature procedure by Ke et al. as outlined in Scheme 2.^45^ Folic acid (FA) was dissolved in dimethylsulfoxide and reacted with the azide-PEG_n_-amine, **5**, in the presence of *N,N*′-dicyclohexylcarbodiimide (DCC) and pyridine to give **5**, as a mixture of the α- and γ- isomers. The mixture was found to be difficult to separate by RP-HPLC, so was used without further separation and clicked with the alkyne-bearing terbium conjugates, Tb**7** and Tb**4**.

The final step in the synthesis of Tb**L**^**2**^-Tb**L**^**4**^ was carried out by reacting the respective terminal alkynes, Tb**7** and Tb**4**, with the azido folate, **6** in a copper-catalyzed azide–alkyne cycloaddition using copper(II) sulfate and sodium ascorbate as a reducing agent to produce the copper(I) catalyst in situ. All complexes were purified by RP-HPLC in acidic conditions to yield the conjugates as yellow powders and as the 1,4-disubstituted triazoles. Complexes Tb**L**^**2**^ and Tb**L**^**4**^ were obtained as mixtures of the α- and γ-regioisomers as these were difficult to separate by HPLC. Tb**L**^**3**^, however, underwent successive rounds of HPLC purification to initially separate a purified mixture of Tb**L**^**3**^ and then separate the α- and γ-regioisomers to yield Tb**L**^**3 major**^ and Tb**L**^**3 minor**^. Due to the paramagnetic nature of the terbium center, regioisomers could not be assigned as the α- or γ-regioisomer.

Synthesis of Tb**L**^**5**^ was carried out in the absence of a copper catalyst and the reaction was promoted by additional ring strain imposed by the two conjugated benzene rings on the Tb-DOTA-DBCO. In addition to the α- and γ-regioisomers of **6**, the SPAAC affords both the 1,3- and 1,4-triazole products resulting in a mixture of 4 isomers. Due to the high similarity of the complexes, these could not be separated by RP-HPLC and remained as a mixture of the four isomers.

### 2.2 Photophysical Characterization

#### 2.2.1 Electronic Spectroscopy

**Figure 1:**
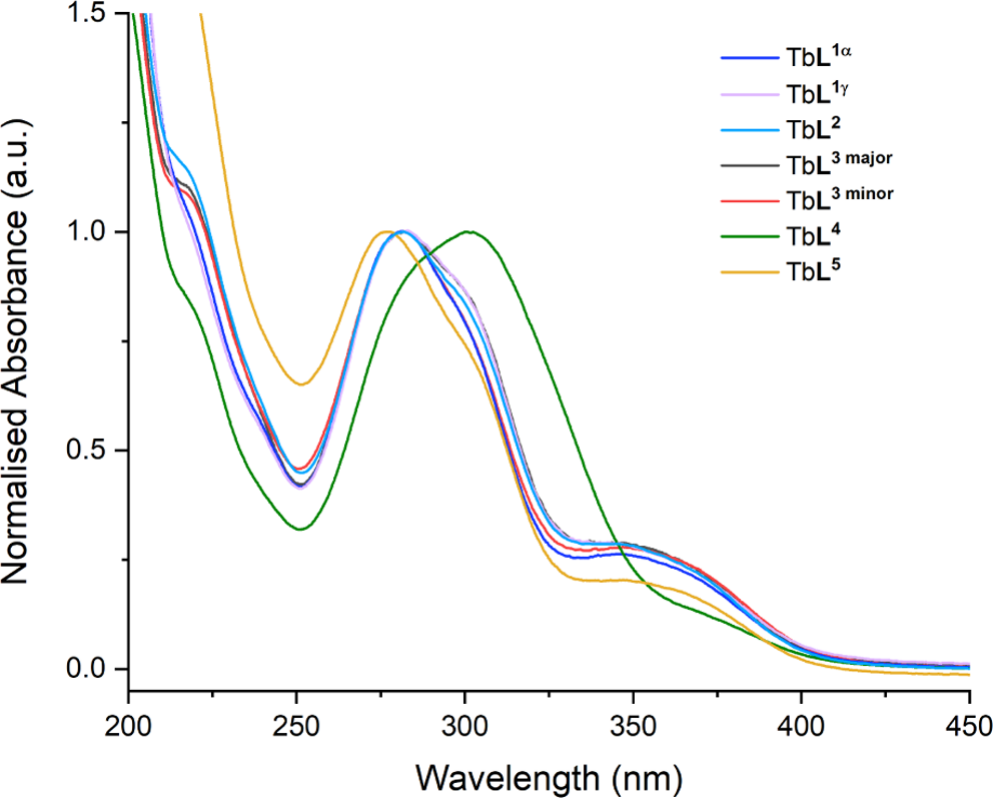
Normalized absorption spectra of Tb**L**_**1**_-Tb**L**_**5**_ in H_2_O.

The absorption spectra of the terbium folate complexes are shown in Figure 1. The absorption spectrum of folic acid is characterized by two absorption bands in the region of 250–300 and 300–400 nm, which correspond to the π → π* transition for the pterin and the *n* → π* transition for the *p*-amino benzoyl acid moieties respectively.^46^ All the terbium complexes exhibit these two distinct absorption characteristics of the FA moiety. Differences are observed for Tb**L**_,_^**4**^ which is red-shifted and broadened due to the overlap with the acetophenone moiety; similar absorbance spectra of acetophenone-folate complexes have been reported by Quici et al.^16^

**Figure 2:**
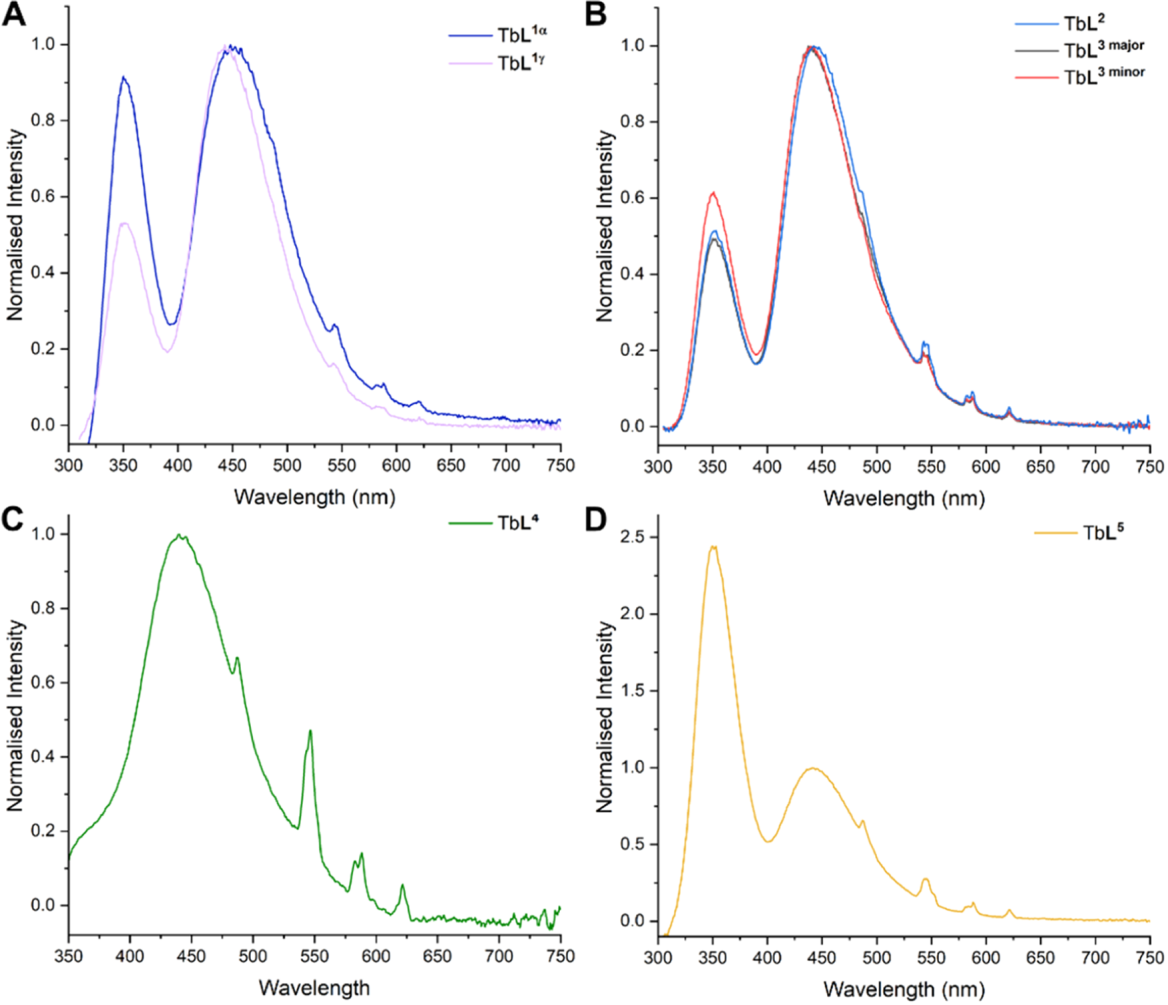
Normalized fluorescence emission spectra of Tb-folate complexes in H_2_O (A) α: λ_ex_ = 283 nm, γ: λ_ex_ = 277 nm; (B) λ_ex_ = 282 nm; (C) λ_ex_ = 301 nm; (D) λ_ex_ = 277 nm.

All complexes, except for Tb**L**^**4**^, were excited through the pterin absorption band at ca. 280 nm using weak indirect excitation *via* Förster energy transfer to the terbium center. For Tb**L**^**4**^, indirect Dexter-mediated energy transfer through the acetophenone chromophore was achieved following excitation at 301 nm (Figure 2C). The complexes all display strong fluorescence from the folate moiety (Figure 2), with the dominant pterin emission peak at ∼440 nm, while the characteristic terbium emission peaks are just visible at 545, 588, and 621 nm corresponding to the ^5^D_4_ → ^7^F_J_ (*J* = 5–3) respectively. This suggests that the variations in the structure with the α- and γ- folate regioisomers makes minimal difference in the absorption of the terbium emission spectra as shown for Tb**L**^**1**^ and Tb**L**^**3**^. Similarly, the length of PEG chain appears to make little difference to the fluorescence emission for Tb**L**^**2**^ (*n* = 2) and Tb**L**^**3**^ (*n* = 5), as shown in Figure 2b. Furthermore, in the case of Tb**L**^**4**^, it is clear that absorption is occurring through both the folate and the carboxyphenacyl chromophore; thus, while the lanthanide emission is stronger in this case, there is still a substantial background of folate fluorescence.

**Figure 3:**
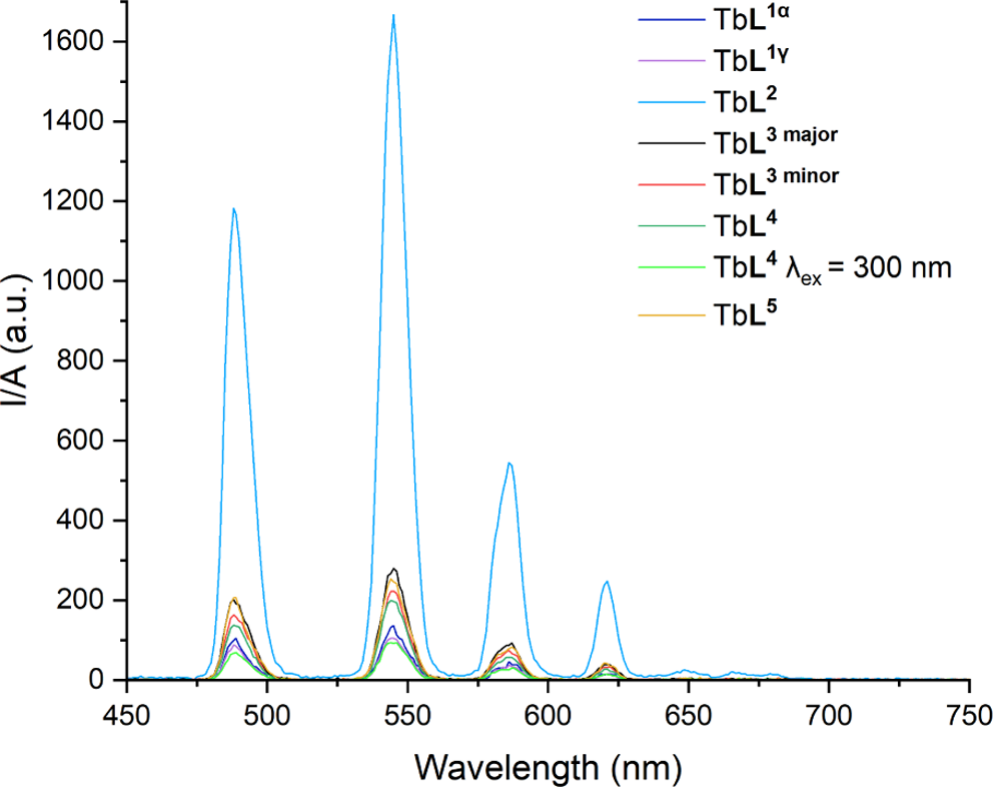
Phosphorescence emission spectra of Tb-folate complexes in [Tb**L**] = 25 μM in H_2_O, λ_ex_ = 280 nm. Spectra corrected for absorbance.

Therefore, time-gating techniques can be employed to remove the short-lived fluorescence from the folate and the cell. This is analogous to phosphorescence emission, where the phosphorescence emission is recorded over a longer period of time to capture the longer-lived terbium emission with an initial delay to remove the biological autofluorescence and organic folate-centered emission. Translating this over would utilize techniques such as Phosphorescence Lifetime Imaging Microscopy (PLIM). The phosphorescence emission spectra for Tb**L**^**1**^-Tb**L**^**5**^ are shown in Figure 3, where it can be seen that the folate moiety is able to act as a sensitizer to indirectly excite the terbium center. In such a system, a variety of processes are possible: long-lived luminescence from the lanthanide and short-lived fluorescence from the ligand offer competitive routes to radiative emission. Interestingly, the length of the PEG chain spacer is important for the efficiency of this energy transfer, with the shortest PEG chain for Tb**L**^**2**^ (*n* = 2) resulting in the greatest emission when recorded under the same conditions. Subtle differences are also observed between the Tb**L**^**1**^ (PEG *n* = 6) with the weakest emission and the intermediate emission from Tb**L**^**3**^, Tb**L**^**4**^ and Tb**L**^**5**^ (PEG *n* = 5). For Tb**L**^**4**^, when excited at 300 nm through the acetophenone moiety, weaker emission is observed, therefore there may be other pathways that quench the excited state. The folate sensitization occurs through space *via* Förster energy transfer and is affected by the PEG spacer length.

#### 2.2.2 Lifetimes and *q* Values

The emission lifetimes of the complexes Tb**L**^**1**^-Tb**L**^**5**^ were measured to determine the number of water molecules, *q*, coordinated to the metal center and are summarized in Table 1. This is calculated from the modified Horrocks equation that accounts for the difference in emission lifetimes when quenched by O–H or O–D oscillators.^47^ This is useful to inform future time-gating measurements for cell imaging.

**Table 1:** Photophysical Data for Terbium Conjugates TbL^1^-L^5^ Measured in H_2_O

**complex**	**λ**_**ex**_**(nm)**	***k***_**H**_**2**_**O**_**(m s**^**–1**^**)**	***k***_**D**_**2**_**O**_**(m s**^**–1**^**)**	***q***	**ε** (M^**–1**^ **cm**^**–1**^**)**	**Φ**^a^**(%)**
TbL^1α^	283	0.52	0.30	0.8	16 302	0.9
TbL^1γ^	277	0.55	0.35	0.7	22 635	0.9
TbL^2^	282	0.52	0.31	0.7	16 031	5.8
TbL^3 major^	282	0.52	0.32	0.7	20 004	1.7
TbL^3 minor^	282	0.54	0.32	0.7	25 137	1.5
TbL^4^	301	0.73*	0.41*	1.3*	25 634	0.5
TbL^5^	277	0.52	0.29	0.9	20 633	0.6

a Quantum yield measured by using quinine sulfate in 0.5 M H_2_SO_4_(aq) as the standard (Φ= 0.55).^48^*B*iexponential decay fit *y* = A1 × exp(−*x*/*t*1) + A2 × exp(−*x*/*t*2) + *y*0. A1 (fast decay weighting) = 0.82, A2 (slow decay weighting) = 0.18 for H_2_O and D_2_O. *k*_H_2_O_ fast = 11.37 ms^–1^; *k*_D_2_O_ fast = 11.22 ms^–1^. *k* = 1/*t*2; Estimated error ±10%.

The complexes all display weak molar extinction coefficients, which primarily arises from the folate moiety. Low quantum yields (Φ) were recorded for all complexes, with Tb**L**^**2**^ displaying the highest Φ. For these conjugates to function as fluorescent probes for the standard microscopy techniques, these are too weakly emissive to compete with the cellular autofluorescence. This is highlighted when comparing to the FDA-approved folate-heptamethine cyanine dye, OTL38 which has a Φ = 15.1% and ε = 272 000 M^–1^ cm^–1^ with λ_ex_ = 776 nm, λ_em_ = 793 nm.^10^ This is able to excite at a lower energy wavelength, which is less damaging for biological imaging as well as emitting in the NIR-window where there is a reduced fluorescence from the cell.

#### 2.2.3 Folate Receptor Fluorescent Folic Acid Binding Competition Assay

**Figure 4:**
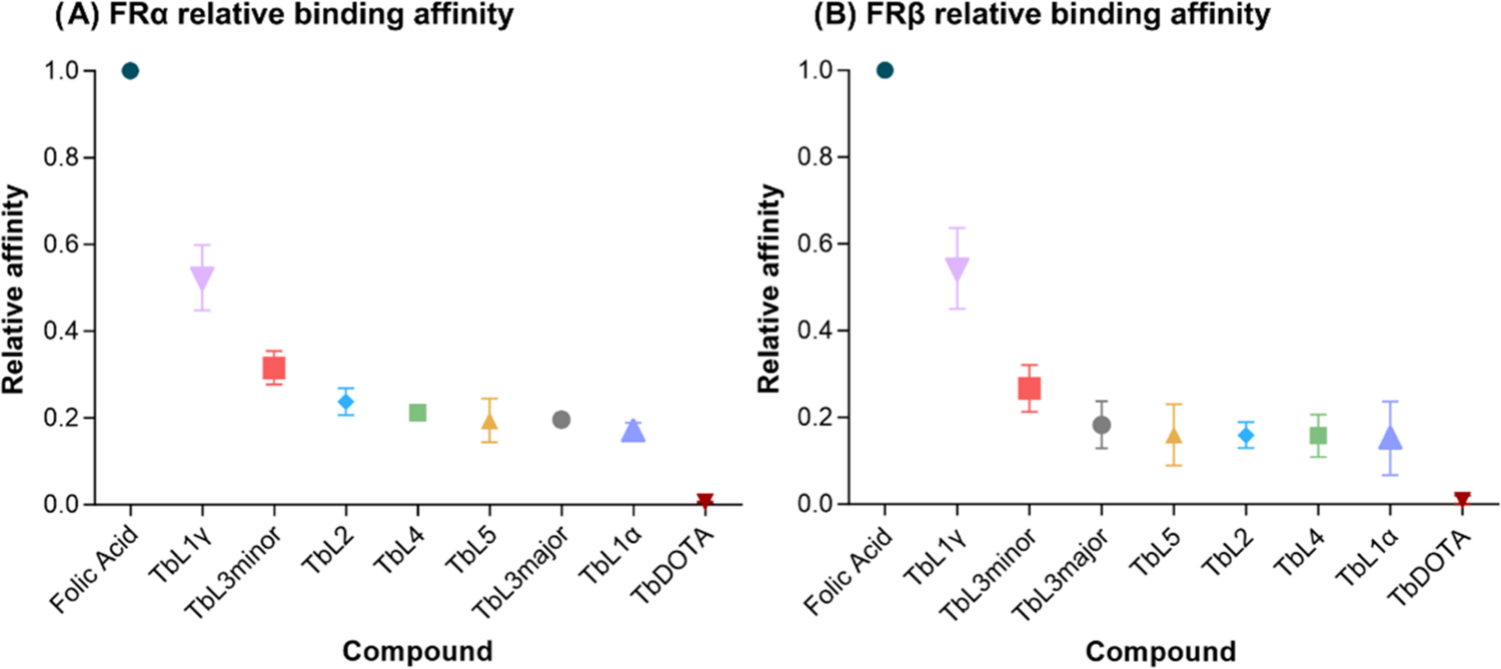
Relative binding affinities for folate-terbium conjugates compared to folic acid. (A) FRα on KB cells; **(B**) FRβ on CHO/FRβ transfected cells.

To assess the binding affinity of the terbium-folate conjugates, a flow cytometry binding competition assay was carried out with folate-FITC and increasing concentrations of terbium-folate conjugates with FRα and FRβ expressing cells. The affinity of FRα and FRβ for the terbium-folate conjugates is expressed relative to folic acid, which is given the value of 1. Based on the folate-FITC FR binding competition data, all terbium-folate conjugates, except the nonfolate-conjugated compound Tb-DOTA, showed relative binding affinities to FRα and FRβ being 2–5 fold lower than folic acid (Figure 4a,b, respectively). Notably, Tb**L**^**1γ**^ had the highest relative binding affinity of all folate-conjugated Tb compounds. A slightly lower relative binding affinity was observed for Tb**L**^**3 minor**^ (one-third of folic acid). All other compounds Tb**L**^**2**^, Tb**L**^**3major**^, Tb**L**^**4**^, Tb**L**^**1α**^ had comparable relative binding affinities around 20% of folic acid. All compounds, except Tb-DOTA showed dose-dependent reduction of folate-FITC signal.

It is important to note that FR relative binding affinities for Tb-folate conjugates should be considered in comparison of 5-methyltetrahydrofolate as the major circulating form of folate in plasma. Given that FR affinities for 5-methyltetrahydrofolate are approximately 10-fold lower than folic acid,^4,49^ this implies that Tb-folate conjugates can displace natural folates from the receptor with proficient binding affinity.

Beyond binding affinities, we also examined whether the Tb-folate conjugates harbored any toxic effects. Following incubations of KB cells for 24 h with 500 nM of the Tb conjugates, no effects on cell viability were observed (results not shown).

#### 2.2.4 *In Silico* Assessment of Membrane Permeability

To predict the behavior of the Tb-folate compounds regarding the permeability through the blood-brain barrier and the cell membrane, *in silico* methods were utilized. The main parameters of focus were the total free energy penalty for the ligand to change state and enter the membrane (DG_in_) and the predicted permeability through model cell lines such as Ralch Russ canine kidney cell (RRCK), Madin-Darby canine kidney cells (MDCK) as a model of the blood-brain barrier, and Caco-2 cells as a model of the gut-blood barrier.^50,51^ Glycerol was used as a positive control for permeabilization. All Tb-folate compounds showed poor permeability profiles, both the blood-brain and the gut-blood barriers, indicating that these compounds cannot be orally administered or used for imaging brain inflammation or cancer (Table 2).

**Table 2:** *In Silico* Assessment of Membrane Permeability^a^

**ID compounds**	**DG**_**in**, _kcal/mol^b^	**log *P***_**m**_**(log Perm RRCK)**^c^	**Perm**, nm/s	***P***_**Caco, sm/s**_**(QPPCaco)**^d^	*^**P**^*_**MDCK, sm/s**_**(QPPMDCK)**^e^
DOTA	28.7	–7.24	0.58	0.0	0.0
DOTA-Ca	38.4	–6.94	1.15	n/c	n/c
ChEBI_27470	44.2	–7.28	0.52	0.0	0.0
glycerol	8.5	–4.37	426.58	351.0	159.4
L1- α	38.4	–7.32	0.48	0.0	0.0
L1- γ	40.2	–7.53	0.30	0.0	0.0
L2-α	34.6	–6.77	1.70	0.1	0.0
L2-γ	35.7	–6.95	1.12	0.1	0.1
L3- α	33.4	–6.95	1.12	0.1	0.1
L3- γ	36.3	–7.21	0.62	0.2	0.1
L4- α	34.6	–7.19	0.65	0.2	0.1
L4- γ	35.7	–7.36	0.44	0.1	0.0
L5- α	33.1	–7.43	0.37	0.1	0.0
L5- γ	36.8	–7.72	0.19	0.0	0.0

a n/c -No calculation

b ^**DG**^**in** is the total free energy penalty for the ligand to change state and enter the membrane.

c Log P_m_: Logarithm of the RRCK permeability in cm/s

d Predicted apparent Caco-2 cell permeability in nm/sec. Caco-2 cells are a model for the gut-blood barrier. QikProp predictions are for nonactive transport (<25 poor, > 500 great).

e Predicted apparent MDCK cell permeability in nm/sec. MDCK cells are considered to be a good mimic for the blood-brain barrier. QikProp predictions are for nonactive transport (<25 poor, > 500 great).

Finally, we focused on the Tb-folate compounds concerning Lipinski’s and Jorgensen’s rules to model their bioavailability. Lipinski’s rule of five assesses this from the number of hydrogen bond donors and acceptors, molecular weight, and partition coefficient,^52^ and Jorgensen’s rule of three is to model the permeability and solubility of the compounds. All compounds are characterized by low bioavailability (SI section 6).

### 2.3 Summary

This work has involved the synthesis of a range of folate-terbium bioconjugates, with the γ-isomer of the folic acid showing favorable FR binding over its α-analogue. The length of the PEG spacer was shown to alter the through space Förster energy transfer with shorter chains showing greater phosphorescence. These novel fluorescent complexes are a good initial set of probes which could be used for PLIM. The probes can be further improved by including a sensitizing chromophore to enhance fluorescent emission for use in more readily available microscopic techniques. Alternatively using different lanthanides in the same constructs, these could be extended for use as an MRI contrast agent (by using Gd) or a theragnostic for PET imaging and treatment (using radioactive ^152^Tb and ^149^Tb or ^177^Lu).

## 3 Conclusions

This work has shown that a wide range of conjugation methods can be used to access folate bioconjugates with lanthanide complexes, and that these complexes retain appropriate affinities for folate receptors (particularly when conjugated via the folate γ-carboxylate). Due to the distance of the UV-active folate compound from the lanthanide center and the inefficient spectral overlap between the folate emission spectrum and the terbium absorption spectrum, the compounds suffered from the characteristic weak molar extinction coefficients associated with direct Ln^3+^ excitation and therefore weak emission. While this can be resolved using time-gated techniques such as PLIM, it is clear that the next generation of luminescent probes must incorporate a sensitizing chromophore which absorbs light outside the range of folate absorption. Until that can be achieved, it will be difficult to achieve the kind of brightness associated with organic fluorophores in conventional confocal microscopy.

The ease with which lanthanide complexes can be incorporated into these systems also suggests another possibility. Use of ^161^Tb or ^177^Lu could be an effective route to PET contrast imaging of folate receptors for use in cancer and inflammation imaging.

## 4 Experimental Procedures

### 4.1 Chemistry

#### 4.1.1 General Procedures

Commercially available solvents and reagents were used without further purification unless otherwise stated. Reagents were laboratory grade and obtained from standard commercial sources including Sigma-Aldrich, Fisher Scientific, Alfa Aesar, Fluorochem, CheMatech, ChemicalPoint, and Insight Biotechnology. Anhydrous solvents used were obtained by passing through a *MBraun MPSP-800* column (degassed with N_2_) and then used immediately. Deionized water was obtained using an Elix Essential water purification system. Propargyl bromide was used as 80 wt % solution in toluene (Sigma-Aldrich). Neutral alumina for column chromatography was purchased from Alfa Aesar (aluminum oxide activated, neutral Brockmann grade I, pore size 58 Å, 60 mesh). Silica gel for column chromatography was purchased from Merck (Geduran, pore size 60 Å, 230–400 mesh, 40–63 μm). Aluminum oxide neutral TLC plates and silica TLC plates were TLC gel 60 purchased from Merck. Brine is a saturated sodium chloride solution. All terbium complexes are confirmed to be over >95% pure by HPLC analysis, except for Tb**L**^**2**^ (85%).

##### 4.1.1.1 NMR Spectroscopy

NMR spectra were recorded at 298 K unless otherwise stated. 600 MHz ^1^H NMR and 151 MHz ^13^C NMR were obtained using a Bruker NEO 600 with broadband helium cryoprobe equipped with a 14 T magnet. 500 MHz ^1^H NMR, 126 MHz ^13^C NMR were recorded on a Bruker Avance III NMR equipped with a 11.75 T magnet. 400 MHz ^1^H NMR and 101 MHz ^13^C NMR were obtained using a Bruker Avance III nanobay 400 NMR equipped with a 9.4 T magnet.

##### 4.1.1.2 Mass Spectrometry

Low-resolution mass spectra were obtained using either an Agilent Technology 1260 Infinity or a Waters LCT Premier XE spectrometer. LC-MS were measured by a Waters LCT Premier benchtop orthogonal acceleration time-of-flight LC-MS system. High-resolution accurate mass spectra were performed by the staff of the Chemistry Research Laboratory, University of Oxford using Bruker μTOF or Waters Micromass GCT spectrometers and recorded to 4 decimal places.

##### 4.1.1.3 UV–vis Spectroscopy

UV–vis spectra were recorded on a Cary60 UV–vis spectrophotometer fitted with a xenon source lamp or on a Jasco V-770 UV–visible/NIR spectrophotometer.

##### 4.1.1.4 Luminescence Spectroscopy

HORIBA Jobin Yvon FluoroLog3 fluorimeter (Hamamatsu R928 detector and a double-grating emission monochromator) was used to acquire the luminescence spectra operated under FluorEssence software. The standard conditions for acquiring emission and excitation spectra are room temperature and steady-stated mode unless otherwise stated. HORIBA Jobin Yvon FluoroLog3 fluorimeter system equipped with a Xenon flash lamp was used to acquire emission lifetimes. Luminescence lifetimes were obtained by tail fit for Tb(III) complexes using Origin software.

##### 4.1.1.5 4′-Ethynyl-2-bromoacetophenone (**1**)

Prepared with minor adaptation from the procedure described by Wu et al.^53^ 1,4-Diethynylbenzene (2.50 g, 19.8 mmol, 1.00 equiv), 1,3-dibromo-5,5-dimethylhydantoin (6.80 g, 23.8 mmol, 1.20 equiv) and *N,N’*-ethylenethiourea (0.304 g, 2.97 mmol, 0.15 equiv) were dissolved in 75 mL aqueous acetone (V_acetone_:V_water_ = 100:1). The reaction was stirred at 45 °C and monitored by TLC (4–7 h). Upon completion, the reaction mixture was cooled to 15 to 25° and concentrated under vacuum. The residue was purified by column chromatography on short silica gel (pentane/ethyl acetate, 100:0 to 95:5), followed by a second column (pentane/Et_2_O, 95:5 to 88:12) to afford the product 1 as a yellow oil/solid (0.848 g, 3.8 mmol, 19%). *R*_f_ = 0.36 (pentane/EtOAc, 9:1).

^**1**^**H NMR** (500 MHz, CDCl_3_) δ 7.94 (m, 2H, Ar***H***), 7.59 (m, 2H, Ar***H***), 4.43 (s, 2H, C***H***_***2***_Br), 3.29 (s, 1H, C≡C***H***) ppm. ^**13**^**C NMR** (126 MHz, CDCl_3_) δ 190.7 (***C***=O), 133.7 (Ar***C***), 132.6 (2 × Ar***C***), 129.0 (2 × Ar***C***), 128.0 (Ar***C***), 82.6 (***C***≡CH), 81.2 (C≡***C***H), 30.7 (***C***H_2_Br) ppm. **GC EI**^**+**^**MS** found 221.96803 C_10_H_7_BrO requires 221.96748 (|Δ *m*/*z*| = 9.46 ppm).

##### 4.1.1.6 *t*Bu_3_DO3AH: 1,4,7-Tris(tert-butoxycarbonylmethyl)-1,4,7,10-tetraazacyclododecane hydrobromide salt (**2**)

Prepared by the procedure described by Dadabhoy et al.^54^ using cyclen (6.0 g, 34.8 mmol, 1.0 equiv) to yield product **2** as a white solid (9.09 g, 44%).

^**1**^**H NMR** (400 MHz, CDCl_3_): δ 10.05–9.87 (br s, 2H, NH·HBr), 3.31 (s, 4H, COCH_2_N), 3.23 (s, 2H, COCH_2_N), 3.04 (br t, 4H, NCH_2_), 2.87 (br t, 8H, NCH_2_), 2.82 (br t, 4H, NCH_2_), 1.40 (s, 18H, 2 × C(CH_3_)_3_), 1.39 (s, 9H, C(CH_3_)_3_) ppm. The ^1^H NMR data corresponds to reported literature values.^54^**ES-MS** (MeOH): *m*/*z* 515.4 [M + H]+.

##### 4.1.1.7 1-(4′-Ethynyl-2-acetophenone)-4,7,10-tris(tert-butoxycarboxymethyl)-1,4,7,10-tetraazacyclododecane (**3**)

tBu_3_DO3AH, **2** (1.54 g, 2.99 mmol, 1.00 equiv) was added to dry MeCN (15 mL) with potassium carbonate (1.03 g, 7.47 mmol, 2.50 equiv) and stirred for 30 min at 15 to 25 °C. To this, **1** (0.73 g, 3.29 mmol, 1.10 equiv) in dry MeCN (5 mL) was added dropwise. The reaction was left stirring overnight for 16 h at 60 °C under a positive pressure of N_2_. Inorganic solids were removed by filtration and the solvent was removed under reduced pressure. The resulting residue was purified by column chromatography, DCM/MeOH, 100% to 9:1 using 1% increments to yield **3** as a light brown crystalline powder (0.78 g, 35%) *R*_f_ = 0.1 (DCM/iPrOH, 9:1)

^**1**^**H NMR** (400 MHz, CDCl_3_) δ 7.85 (m, ArH), 7.54 (m, 2H, ArH), 4.00 (s, 2H, CH_2_–arm/CH_2_–ring), 3.72–1.79 (m, 23H, CH_2_–arm/CH_2_–ring, C≡CH), 1.43 (s, 27H, (CH_3_)_3_) ppm. ^**13**^**C NMR** (126 MHz, CDCl_3_) δ 199.2 (C=Oketone), 173.2 (2 × C=Oester), 173.0 (C=O_ester_), 135.4 (C_aryl_), 132.5 (2 × CH_aryl_), 127.7 (2 × CH_aryl_), 127.6 (C_aryl_), 82.7 (C≡CH), 82.1, 82.0, 80.9 (C≡CH), 60.5, 56.0, 55.7, 53.6, 52.9, 48.8, 28.3 (CH_3_), 28.21 (CH_3_), 28.15 (CH_3_), 28.0 (CH_3_), 27.9 (CH_3_) ppm. **ES-MS**(MeOH): *m*/*z* 657.4 [M + H]^+^ LC-MS (MeOH) *m*/*z*: 657.09 [M + H]^+^; HRMS (ES+) found 657.4217 [M + H]^+^; C_36_H_56_O_7_N_4_ requires 657.4222 (|Δ *m*/*z*| = 0.67 ppm).

#### 4.1.2 General Procedure of *Tert*-butyl TFA Deprotection A

To a solution of the ligand, **L** (1.00 equiv) dissolved in DCM (12.5 vol), TFA was added dropwise (12.5 vol, excess) and the reaction was stirred for at least 16 h at 15 to 25 °C, monitored by LCMS. Extra DCM (∼250 vol) was added and removed under reduced pressure; this was carried out a further three times with MeOH (∼250 vol). The residue was dissolved in minimal MeOH and triturated with diethyl ether, centrifuged and the supernatant was discarded. This trituration was repeated (total twice) and the solid was dried under high vacuum.

##### 4.1.2.1 1-(4′-Ethynyl-2-acetophenone)-4,7,10-tris(carboxymethyl)-1,4,7,10-tetraazacyclododecane triflate salt/·xTFA (**4**)

Following general procedure A, compound **4** was prepared from **3**(0.2 g) to yield product **4** as a dark yellow powder (0.134 g, 0.274 mmol, 90%).

^**1**^**H NMR** (600 MHz, D_2_O/CD_3_OD (1:1)) δ 7.96 (m, 2H, ArH), 7.62 (m, 2H, ArH), 4.52 (br s, 1H), 3.88–3.69 (m, 8H, CH_2-arm_, CH_2-ring_, C≡CH), 3.59–3.32 (s, 9H, CH_2-arm_, CH_2-ring_,), 3.27–3.02 (m, 8H, CH_2-arm_, CH_2-ring_) ppm.

^**13**^**C NMR** (151 MHz, D_2_O/CD_3_OD (1:1)) δ 163.4 (q, ^2^J_C–F_ = 35.0 Hz), 133.3 (CH_aryl_), 129.1 (CH_aryl_), 117.6 (q, ^1^J_C–F_ = 292.0 Hz), 83.6 (C≡CH), 82.6 (C≡CH), 59.3, 57.2, 55.1, 54.3, 52.1 ppm. ^**19**^**F NMR** (565 MHz, D_2_O/CD_3_OD (1:1)) δ −77.5 ppm. **ES-MS**(MeOH): *m*/*z* 489.3 [M + H]^+^, 511.3 [M + Na]^+^ LC-MS (MeOH) *m*/*z*: 489.0 [M + H]^+^, 976.9 [M + 2H]^2+^; HRMS (ES+) found 489.2344 [M + H^+^]; C_24_H_33_O_7_N_4_ requires 489.2344 (|Δ *m*/*z*| = 0.02 ppm).

#### 4.1.3 General Procedure of Tb Complexation B

To a solution of the ligand, L (1.00 equiv), Tb(OTf)_3_ (1.05 equiv) was added, and the reaction mixture was stirred at 60 °C for 30 min. The pH was adjusted to 4 by dropwise addition of an aqueous 1 M NaOH solution. The reaction was then allowed to stir at 60 °C for 48 h. The pH of this solution was adjusted to 7 by addition of 1 M NaOH and the solvent removed under reduced pressure.

##### 4.1.3.1 Tb**4**

Following general procedure B in MeOH, the title compound was prepared from **4** (0.15 g, 0.31 mmol, 1.00 equiv) and Tb(OTf)_3_(0.20 g, 0.33 mmol, 1.05 equiv) and purified by column chromatography on neutral alumina (MeCN/H_2_O, 8:2 to 7:3) to yield the product as a pale beige-pink powder (0.108 g, 0.17 mmol, 79%). *R*_f_ = 0.13 (MeCN/H_2_O, 7:3).

^**1**^**H NMR** (400 MHz, MeOD) δ 354.9, 316.5, 291.3, 264.8, 228.3, 215.9, 164.6, 154.7, 106.0, 93.5, 23.9, 17.5, – 10.9, – 13.2, – 24.6, – 26.3, – 31.4, – 36.4, – 48.9, – 51.0, – 58.5, – 78.3, – 101.8, – 105.5, – 125.7, – 133.5, – 157.8, – 180.1, – 213.8, – 241.9, – 249.3, – 256.3, – 352.2, – 360.3, – 477.3 ppm. Only major resolved peaks outside the +10 to −10 ppm range are reported. ^**19**^**F NMR** (377 MHz, MeOD) δ – 77.7, – 82.0 ppm.

**ES-MS** (MeOH) *m*/*z* 645.2 [M + H]^+^, 667.2 [M + Na]^+^; LC-MS (MeOH) *m*/*z*: 645.736 [M + H^+^]; **HRMS** (ES^+^), found (*m*/*z*): 645.1359 [M + H]^+^; C_24_H_30_N_4_O_7_^159^Tb requires 645.1362 (|Δ *m*/*z*| = 0.51 ppm).

**UV–vis** (H_2_O): λ_max_ 292 nm.

**Luminescence lifetimes**: H_2_O: 1.42 ms, D_2_O: 2.27 ms, *q* = 1.0.

##### 4.1.3.2 N^10^-(TFA)-pteroic acid (S1)

Prepared by the procedure described by Yamaguchi et al.^55^ using pteroic acid (0.5 g, 1.60 mmol, 1.00 equiv) to yield **S1** as a brown solid (0.48g, 1.17 mmol, 73%) and used directly without further purification.

^**1**^**H NMR** (600 MHz, DMSO-*d*_6_/D_2_O (8:2)) δ 8.56 (s, 1H, H1), 7.94 (d, ^3^*J*_H–H_ = 8.3 Hz, 2H, H4), 7.57 (d, ^3^*J*_H–H_ = 8.1 Hz, 2H, H3), 5.09 (s, 2H, H2) ppm. ^**13**^**C NMR** (151 MHz, DMSO-*d*_6_/D_2_O (8:2)) δ 167.4 (COOH), 161.7, 156.6 (q, ^2^J_C–F_ = 35.6 Hz, COCF_3_), 155.9, 154.3, 150.1 (C1), 145.5, 143.6, 131.8, 131.2, 131.1 (C4), 129.7 (C3), 128.7, 121.4, 116.7 (q, ^1^J_C–F_ = 288.6 Hz, CF_3_), 54.4 (C2) ppm. ^**19**^**F NMR** (377 MHz, CDCl_3_) δ – 66.2 (CF_3_), 73.9, 74.1 ppm.

**ES-MS** (MeOH) *m*/*z* 409.069 [M + H]+, HRMS (ES+), found (*m*/*z*): 409.0865 [M + H]+; C_16_H_12_O_4_N_6_F_3_ requires 409.0867. (|Δ*m*/*z*|=0.43 ppm).

##### 4.1.3.3 FA-PEG_6_-EDA-NH_2_ (**S2α** or **S2γ**)

Prepared with minor adaptation from the procedure described by Chen et al.^22^ and Kularatne et al.^44^ Refer to Scheme S1 for an overview of the synthetic route. The procedure was carried out in parallel batches of four. 1,2-diaminoethane trityl resin (1.2–1.7 mmol/g, 70 mg, 0.1 mmol) was swollen with DCM (3 mL) for 2 h and then drained, followed by DMF (3 mL) for 2 h and then drained through a Biotage phase separator column. After swelling the resin, a solution of Fmoc-PEG_6_–OH (86 mg, 0.15 mmol, 1.50 equiv), HATU (57 mg, 0.15 mmol, 1.50 equiv), and DIPEA (35 μL, 0.2 mmol, 2.00 equiv) in DMF (4 mL) was stirred for 15 min before addition to the resin. N_2_ was bubbled through the suspension overnight, and the resin was washed with DMF (3 × 15 mL) followed by i-PrOH (3 × 15 mL). The resin was then swollen in DMF (5 mL) for at least 30 min before the next step. The above resin was incubated with 20% piperidine in DMF (5 mL) for 30 min to remove the Fmoc- protection, then followed by washings with DMF (3 × 15 mL) and i-PrOH (3 × 15 mL). The resin was then swollen in DMF (5 mL) for at least 30 min before the next step. The above sequence was repeated for two more coupling steps for conjugation of γ-isomer: Fmoc-Glu-OtBu (CAS: 84793-07-7) or α-isomer: Fmoc-Glu(OtBu)–OH (CAS: 71989-18-9) (64 mg, 0.15 mmol, 1.50 equiv) and N^10^-TFA-Ptc–OH (61 mg, 0.15 mmol, 1.50 equiv). The product was cleaved from the resin using a TFA: H_2_O: TIPS mix (95:2.5:2.5) (5 mL) for 2–3 h at room temperature unstirred. The filtrate was collected, and the resin was washed with DCM (3 × 15 mL) and i-PrOH (3 × 10 mL) and the filtrate and washes were concentrated under vacuum. The product was washed with copious amounts of DCM that was removed under reduced pressure, then washed with MeOH and removed. The concentrated product was dissolved in minimal MeOH (1–3 mL) and precipitated in cold diethyl ether (∼400 mL) and centrifuged (4000 rpm, 4 °C, 10 min) and a yellow solid isolated that was characterized by MS (ES-MS (MeOH) *m*/*z* 915.3 [M + H]^+^). The yellow precipitate was stirred with saturated Na_2_CO_3_ solution at room temperature to cleave the trifluoroacetyl protecting group for ∼1 h followed by MS. The mixture was neutralized to pH 7 with 2 M HCl (aq.) and the solvent was removed under reduced pressure. The residue was dialyzed for at least 24 h. The precipitate was purified by HPLC (S3.2 HPLC conditions method 1).

##### 4.1.3.4 S2α

t_R_ (prep) = 1.73 min (crude 0.571g, 0.70 mmol, 174%) (200 mg crude purified by HPLC to give 15.6 mg, 0.019 mmol, 5%)

^**1**^**H NMR** (600 MHz, D_2_O, pH 9) δ 8.62 (s, 1H, ***H1***), 7.69 (m, 2H, ***H4***), 6.82 (m, 2H, ***H3***), 4.58 (s, 2H, ***H2***), 4.39 (dd, ^3^*J*_*H–H*_ = 8.8, 5.5 Hz, 1H, ***H5***), 3.73 (t, ^3^*J*_*H–H*_ = 6.0 Hz, 2H, ***H8***), 3.68–3.38 (m, 26H, ***H10*** and PEG), 3.01 (t, ^3^*J*_*H–H*_ = 6.0 Hz, 2H, ***H11***), 2.52 (t, ^3^*J*_*H–H*_ = 5.9 Hz, 2H, ***H9***), 2.36 (t, *J* = 7.8 Hz, 2H, ***H7***), 2.19–2.12 (m, 1H, ***H6***), 2.12–2.05 (m, 1H, ***H6***) ppm.

^**13**^**C NMR** (151 MHz, D_2_O, pH 9) δ 181.6 (***C***OOH), 174.7 (***C***ONH), 174.5 (***C***ONH), 172.2 (***C***ONH), 169.9 (***C***ONH (ring)), 163.2, 161.0, 155.4, 151.3, 147.8, 147.5 (***C1***), 129.3 (***C4***), 128.1, 120.8, 112.5 (***C3***), 69.6 (PEG), 69.47 (PEG), 69.45 (PEG), 69.43 (PEG), 69.41 (PEG), 68.9 (PEG), 66.5 (***C8***), 55.1 (***C5***), 45.7 (***C2***), 39.4 (***C11***), 39.1 (***C10***), 38.5, 36.0, 35.9 (***C9***), 33.9 (***C7***), 27.7 (***C6***) ppm. **ES-MS** (MeOH) *m*/*z* 819.3 [M + H]^+^; LC-MS (H_2_O) *m*/*z*: 818.4 [M + H]^+^.

##### 4.1.3.5 S2γ

t_R_ (prep) = 4.7 min. (63.2 mg, 0.078 mmol, 19%).

^**1**^**H NMR** (600 MHz, D_2_O, pH 9) δ 8.64 (s, 1H, ***H1***), 7.69 (m, 2H, ***H4***), 6.85 (m, 2H, ***H3***), 4.62 (s, 2H, ***H2***), 4.37 (dd, ^3^*J*_*H–H*_ = 9.5, 4.0 Hz, 1H, ***H5***), 3.79–3.65 (m, 2H, ***H8***), 3.62–3.43 (m, 20H, PEG), 3.38 (q, *J* = 6.3 Hz, 4H, ***H10*** and PEG), 3.29–3.14 (m, 2H, PEG), 2.92 (t, ^3^*J*_*H–H*_ = 5.8 Hz, 2H, ***H11***), 2.52 (t, ^3^*J*_*H–H*_ = 6.0 Hz, 2H, ***H9***), 2.40 (dt, ^2^*J*_*H–H*_ = 14.6, ^3^*J*_*H–H*_ = 7.3 Hz, 1H, ***H7***), 2.34 (dt, ^2^*J*_*H–H*_ = 14.6, ^3^*J*_*H–H*_ = 6.9 Hz, 1H, ***H7***), 2.26 (dtd, ^2^*J*_*H–H*_ = 14.3, ^3^*J*_*H–H*_ = 7.2, 4.1 Hz, 1H, ***H6***), 2.08 (ddt, ^2^*J*_*H–H*_ = 14.1, ^3^*J*_*H–H*_ = 8.8, 7.1 Hz, 1H, ***H6***) ppm.^**13**^**C NMR** (151 MHz, D_2_O, pH 9) δ 178.6 (***C***OOH), 175.6 (***C***ONH), 174.6 (***C***ONH), 172.7 (***C***ONH), 169.2 (***C***ONH (ring)), 155.5, 151.1, 147.9, 147.5 (***C1***), 129.1 (***C4***), 128.2, 121.5 (***C3***), 112.7, 69.4 (PEG), 69.4 (PEG), 69.2 (PEG), 68.6 (PEG), 66.6 (***C8***), 55.2 (***C5***), 45.8 (***C2***), 39.5 (***C11***), 39.3 (***C10***), 38.9, 35.9 (***C9***), 32.7 (***C7***), 27.7 (***C6***) ppm. **ES-MS** (MeOH) *m*/*z* 819.3 [M + H]^+^.

##### 4.1.3.6 Folate-PEG_6_-DOTA (**L**^**1αγ**^)

Prepared with minor adaptation from the procedure described by Chen et al.^22^ and Kularatne et al.^44^ FA-PEG_6_-EDA-NH_2_, **S2** (0.150 g, 0.183 mmol 1.00 equiv) was added to a flame-dried flask and dissolved in DMSO (1.8 mL, 0.1 M final concentration). DIPEA (112 μL, 0.641 mmol, 3.50–3.80 equiv) and DOTA-NHS ester (0.153 g, 0.201 mmol, 1.10 equiv) were added and the reaction was stirred at 20 °C overnight under an Ar atmosphere. The reaction was monitored by LC-MS and upon completion (∼24 h) the unreacted DOTA-NHS ester was cleaved by the addition of water (1 mL) and stirred for 30 min. The reaction mixture was concentrated under reduced pressure and the crude product precipitated by dropwise addition into vigorously stirred cold (0 °C) acetone (∼600 mL). The suspension was centrifuged (4000 rpm, 4 °C, 10 min), and the yellow solid isolated. The solid was dissolved in water and freeze-dried to remove trace DMSO and yield the crude product as a yellow powder. The precipitate was purified by HPLC (S3.1 HPLC conditions method 1).

##### 4.1.3.7 L^1α^

Crude yield: (0.221 g, 0.183 mmol, 100%). Approximately 100 mg of the crude was purified HPLC to afford the pure product (5.1 mg, 0.004 mmol, 2%).

^**1**^**H NMR** (600 MHz, D_2_O, pH 9) δ 8.65 (s, 1H, ***H1***), 7.72 (d, ^3^*J*_*H–H*_ = 8.8 Hz, 2H, ***H4***), 6.87 (d, ^3^*J*_*H–H*_ = 8.8 Hz, 2H, ***H3***), 4.64 (s, 2H, ***H2***), 4.38 (dd, ^3^*J*_*H–H*_ = 8.9, 5.4 Hz, 1H, ***H5***), 3.76 (t, *J* = 6.2 Hz, 2H, ***H8***), 3.72–3.17 (m, 30H, C***H***_***2***-ring_/ C***H***_***2***-arm_/PEG/ ***H10***/ ***H11***), 3.12 (s, 5H, cyclen-ring), 2.90 (br. s, 5H, cyclen-ring), 2.53 (t, ^3^*J*_*H–H*_ = 6.2 Hz, 1H, ***H9***), 2.35 (td, ^3^*J*_*H–H*_ = 7.5, 2.1 Hz, 2H, ***H7***), 2.18–2.03 (m, 2H, ***H6***) ppm.

^**13**^**C NMR** (151 MHz, D_2_O, pH 9) δ 181.6 (C^7^***C***OOH), 174.5, 174.0, 172.6, 171.0, 170.1 (***C***ONH(ring)), 163.5, 155.5, 151.4, 147.9, 147.6 (***C1***), 129.4 (***C4***), 129.1, 128.2, 121.0, 112.7 (***C3***), 69.59 (PEG), 69.56 (PEG), 69.53 (PEG), 69.50 (PEG), 69.47 (PEG), 69.42 (PEG), 69.40 (PEG), 68.9 (PEG), 66.7 (***C8***), 56.9 (cylen-arm), 56.7 (cylen-arm), 56.4 (cylen-arm), 55.0 (***C5***), 51.2, 49.7 (cylen-ring), 49.5 (cylen-ring), 45.8 (***C2***), 39.1 (cyclen-arm), 39.0 (cyclen-ring/***C10***/***C11***), 38.7 (cyclen-ring/***C10***/***C11***), 38.4 (cyclen-ring/***C10***/***C11***), 36.0 (***C9***), 33.9 (***C7***), 27.6 (***C6***) ppm.

##### 4.1.3.8 L^1γ^

Crude yield: (63.8 mg, 0.053 mmol, > 100%).

^**1**^**H NMR** (600 MHz, D_2_O, pH 9) δ 8.71 (s, 1H, ***H1***), 7.63 (d, ^3^*J*_*H–H*_ = 8.6 Hz, 2H, ***H4***), 6.70 (d, ^3^*J*_*H–H*_ = 8.6 Hz, 2H, ***H3***), 4.53 (s, 2H, ***H2***), 4.38 (dd, ^3^*J*_*H–H*_ = 9.4, 4.2 Hz, 1H, ***H5***), 3.93–2.96 (m, 52H, C***H***_***2***-arm_/C***H***_***2***-ring_/PEG/ ***H10***/***H9***), 2.54 (t, ^3^*J*_*H–H*_ = 6.2 Hz, 2H, ***H9***), 2.46–2.32 (m, 2H, ***H7***), 2.26 (dtd, ^2^*J*_*H–H*_ = 14.3, ^3^*J*_*H–H*_ = 7.4, 4.6 Hz, 1H, ***H6***), 2.14–2.05 (m, 1H, ***H6***) ppm. Crude NMR likely to contain DOTA as an impurity.

^**13**^**C NMR** (151 MHz, D_2_O, pH 9) δ 178.6 (C^5^***C***OOH), 178.0, 175.7, 174.0, 172.3, 169.8, 169.0 (C^5^NH***C***O), 154.4, 150.7, 149.6 (***C1***), 149.1 (***C1***), 129.0 (***C4***), 127.2, 121.3, 112.3 (***C3***), 69.5 (PEG), 69.4 (PEG), 69.3 (PEG), 68.7 (PEG), 66.7 (***C8***), 56.4 (cylen-arm), 56.1 (cylen-arm), 56.0 (cylen-arm), 55.9 (cylen-arm), 55.2 (***C5***), 51.8, 50.8, 49.9, 48.3 (cyclen-ring/***C10***/***C11***), 45.5 (***C2***), 38.9 (cyclen-ring/***C10***/***C11***), 38.5 (cyclen-ring/***C10***/***C11***), 36.0 (***C9***), 32.7 (***C7***), 27.8 (***C6***) ppm.

**ES-MS** (MeOH) (mixture of regioisomers) *m*/*z*: 1205.802 [M + H]^+^; HRMS (ES^+^), found (*m*/*z*): 1205.58023 [M + H]^+^; C_52_H_81_O_19_N_14_ requires 1205.57969 (|Δ *m*/*z*| = 0.44 ppm).

**UV–vis** (H_2_O) (mixture of regioisomers) λ_max_ = 281 nm (ε = 20013 M^–1^ cm^–1^), 353 nm.

##### 4.1.3.9 Tb**L**^**1α**^

Following general procedure B in H_2_O, the title compound was prepared from **L**^**1α**^ (65 mg, 0.054 mmol, 1.00 equiv) and Tb(OTf)_3_(0.034 g, 0.057 mmol, 1.05 equiv) and purified by HPLC (S3.2 HPLC Conditions method 2) and lyophilized to yield Tb**L**^**1α**^ as a yellow powder (5.4 mg, 0.004 mmol, 7%). Analytical purity = 98%, t_R_ (ana) = 1.15 min.

^**1**^**H NMR** (500 MHz, D_2_O) δ 252.4, 241.0, 202.6, 189.9, 111.7, 105.0, 76.1, 67.2, 56.1, 47.2, 16.2, 13.2, – 49.0, – 62.0, – 66.4, – 77.5, – 92.9, – 101.1, – 111.3, – 113.7, – 194.9, – 210.2, – 363.2, – 376.0, – 392.5 ppm. Only major resolved peaks outside the +10 to −10 ppm range are reported. **HRMS**(ES^+^) found (*m*/*z*): 1361.4822 [M + H]^+^; C_52_H_78_O_19_N_14_^159^Tb requires 1361.4816 (|Δ*m*/*z*| = 0.47 ppm) **Luminescence lifetimes:** H_2_O: 0.52 ms, D_2_O: 0.30 ms, *q* = 0.80.

##### 4.1.3.10 TbL^1γ^

Following general procedure B, the title compound was prepared from **L**^**1γ**^ (64 mg, 0.054 mmol, 1.00 equiv) and Tb(OTf)_3_(0.034 g, 0.057 mmol, 1.05 equiv) in H_2_O and purified by HPLC (S3.3 HPLC Conditions method 3) and lyophilized to yield Tb**L**^**1γ**^ as a yellow powder (33.3 mg, 0.025 mmol, 46%). Analytical purity = 97%, t_R_ (ana) = 1.13 min.

^**1**^**H NMR** (500 MHz, D_2_O) δ 253.0, 242.4, 239.4, 202.0, 189.7, 111.6, 104.8, 76.3, 68.8, 57.0, 48.0, 16.7, 12.6, – 66.7, – 77.6, – 100.8, – 110.8, – 113.8, – 123.2, – 196.4, – 212.5, – 363.3, – 376.1, – 392.8 ppm. Only major resolved peaks outside the +10 to −10 ppm range are reported. **HRMS**(ES^+^) found (*m*/*z*): 1361.4841 [M + H]^+^; C_52_H_78_O_19_N_14_^159^Tb requires 1361.4816 (|Δ*m*/*z*| = 1.90 ppm). **Luminescence lifetimes:** H_2_O: 0.55 ms, D_2_O: 0.35 ms, *q* = 0.70.

##### 4.1.3.11 Triethylene Glycol Dimethanesulfonate/dimesylate-PEG_2_ (**S3a**)

Prepared by the procedure described by Goswami et al.^56^ and Kohata et al.^57^ Refer to Scheme S2 for overview of the synthesis. Prepared from triethylene glycol (3.0 g, 20.0 mmol, 1.0 equiv) to yield **S3a** as a colorless oil (5.80 g, 18.9 mmol, 95%)

^**1**^**H NMR** (400 MHz, CDCl_3_) δ 4.37 (m, 4H, CH_2_OCH_2_C***H***_2_OMs), 3.77 (m, 4H, CH_2_OC***H***_2_CH_2_OMs), 3.67 (s, 4H, C***H***_2_OCH_2_CH_2_OMs) 3.07 (s, 6H, 2 × SO_3_C***H***_***3***_) ppm.

##### 4.1.3.12 3,6,9,12,15-Pentaoxaheptadecane-1,17-diyl Dimethanesulfonate/dimesylate-PEG_5_ (**S3b**)

Prepared by the procedure described by Goswami et al.^56^ and Kohata et al.^57^ Refer to Scheme S2 for overview of the synthesis. Prepared from hexaethylene glycol (1.46 mL, 5.8 mmol, 1.0 equiv) to yield **S3b** as a colorless oil (2.46 g, 5.61 mmol, 97%).

^**1**^**H NMR** (400 MHz, CDCl_3_) δ 4.38–4.34 (m, 4H, 2 × CH_2_C***H***_***2***_OMs), 3.77–3.72 (m, 4H, 2 × C***H***_***2***_CH_2_OMs), 3.68–3.59 (m, 16H), 3.07 (s, 6H, 2 × SO_3_C***H***_***3***_) ppm. The ^1^H NMR data corresponds to reported literature values.^22^

^**13**^**C NMR** (101 MHz, CDCl_3_) δ 70.70 (***C***H_2_), 70.66 (***C***H_2_), 70.6 (***C***H_2_), 69.5 (CH_2_***C***H_2_OMs), 69.1 (***C***H_2_CH_2_OMs), 37.8 (SO_3_***C***H_3_) ppm.

**ES-MS** (MeOH) *m*/*z* 439.2 [M + H]^+^, 461.2 [M + Na]^+^.

##### 4.1.3.13 1,2-Bis(2-azidoethoxy)ethane (**S4a**)

Prepared by the procedure described by Goswami et al.^56^ and Kohata et al.^57^ Refer to Scheme S2 for overview of the synthesis. Prepared from **S3a** (5.80 g, 18.9 mmol, 1.0 equiv) to yield **S4a** as a pale yellow oil (3.21 g, 16.0 mmol, 80%).

^**1**^**H NMR** (400 MHz, CDCl_3_) δ 3.66–3.63 (overlapping m, 8H, C***H***_2_OC***H***_2_CH_2_N_3_), 3.35 (t, ^3^*J*_H–H_ = 5.01 Hz, 4H, CH_2_OCH_2_C***H***_2_N_3_) ppm.

##### 4.1.3.14 1,17-Diazido-3,6,9,12,15-pentaoxaheptadecane (**S4b**)

Prepared with minor adaptation from the procedure described by Kohata et al.^57^ Refer to Scheme S2 for overview of the synthesis. **S3b** (4.81 g, 10.6 mmol, 1.00 equiv) was dissolved in dry DMF (25 mL) before addition of sodium azide (2.07 g, 46.8 mmol, 2.90 equiv), and the mixture was stirred for 24 h at 65 °C. The reaction mixture was diluted with diethyl ether (80 mL) and filtered through a Celite pad. The filtrate was washed with water (15 mL × 2), followed by brine (20 mL × 3). The isolated organic extracts were combined, and solvent was removed in vacuo. The residue was redissolved in DCM and the inorganic salts were removed by filtration. The filtrate was evaporated to dryness under reduced pressure, affording the product as a pale yellow oil (2.30 g, 6.92 mmol, 65%).

^**1**^**H NMR** (400 MHz, CDCl_3_) δ 3.70–3.63 (m, 20H), 3.38 (t, ^3^J_H–H_ = 5.1 Hz, 4H, C**H**_**2**_N_3_) ppm. The ^1^H NMR data corresponds to reported literature values.^57^

^**13**^**C NMR** (101 MHz, CDCl_3_) δ 70.85, 70.82, 70.77, 70.73, 70.2, 50.8 (**C**H_2_N_3_) ppm.

**ES-MS** (MeOH) *m*/*z* 355.2 [M + Na]^+^.

##### 4.1.3.15 2-(2-(2-Azidoethoxy)ethoxy)ethan-1-amine/azide-PEG_2_-amine (**5a**)

Prepared by the procedure described by Goswami et al.^56^ and Kohata et al.^57^ Refer to Scheme S2 for overview of the synthesis. Prepared from **S4a** (3.21 g, 16.0 mmol, 1.0 equiv) to yield 5**a** as a pale yellow oil 2.14 g, 12.3 mmol, 77%.

^**1**^**H NMR** (400 MHz, CDCl_3_) δ 3.63 (m, 6H, 3 × C***H***_***2***_), 3.47 (m, 2H, C***H***_***2***_), 3.35 (m, 2H, C***H***_***2***_), 2.83 (m, 2H, C***H***_***2***_), 1.39 (br. s, 2H, CH_2_N***H***_2_) ppm.

##### 4.1.3.16 17-Azido-3,6,9,12,15-pentaoxaheptadecan-1-amine/Azide-PEG_5_-amine (**5b**)

Prepared by the procedure described by Goswami et al.^56^ and Kohata et al.^57^ Refer to Scheme S2 for overview of the synthesis. To PEG_5_-diazide, **S5b** (2.30 g, 6.92 mmol, 1.00 equiv), 5% aqueous HCl (25 mL) was added and vigorously stirred at room temperature. To this mixture, a solution of triphenylphosphine (1.63 g, 6.23 mmol, 0.90 equiv) in Et_2_O (18 mL) was added dropwise over ∼3 h. The mixture was stirred for a further 24 h at room temperature. The reaction mixture was washed with ethyl acetate (3 × 100 mL) to remove unreacted starting materials and triphenylphosphine oxide that was formed during the reaction. The aqueous layer was collected, and 20 M potassium hydroxide was added slowly until the pH of the solution was basic (∼pH 12). The product was extracted by washing the aqueous layer with DCM (3 × 100 mL). The organic layer was dried over Na_2_SO_4_, filtered and the filtrate was dried under reduced pressure to afford the product as a pale yellow oil (1.45 g, 4.73 mmol, 68%).

^**1**^**H NMR** (400 MHz, CDCl_3_) δ 3.67–3.57 (m, 18H, OC***H***_***2***_C***H***_***2***_O), 3.48 (t, ^3^*J*_*H–H*_ = 5.2 Hz, 2H, OC***H***_***2***_CH_2_N_3_), 3.36 (t, ^3^*J*_*H–H*_ = 5.1 Hz, 2H, C***H***_***2***_NH_2_), 2.84 (t, ^3^*J*_*H–H*_ = 5.2 Hz, 2H, C***H***_***2***_N_3_), 1.42 (s, 2H, N***H***_***2***_) ppm.

^**13**^**C NMR** (101 MHz, CDCl_3_) δ 73.5 (O***C***H_***2***_CH_2_N_3_), 70.83, 70.77, 70.73, 70.71, 70.5, 70.2, 50.8 (***C***H_2_NH_2_), 41.9 (***C***H_2_N_3_) ppm.

**ES-MS** (MeOH) *m*/*z* 307.2 [M + H]^+^, 329.2 [M + Na]^+^.

#### 4.1.4 General Procedure for Folic Acid Amide Coupling C

Prepared with minor adaptation from the procedure described by Ke et al.^45^ Refer to Scheme S2 for overview of the synthesis. Folic acid (1.00 g, 2.27 mmol, 1.00 equiv) and dicyclohexylcarbodiimide (DCC) (0.935 g, 4.53 mmol, 2.00 equiv) were dissolved in dry DMSO (50 mL) under an inert atmosphere. Pyridine (10 mL) was added to the reaction mixture and stirred for 1 h before addition of the azide-PEG_2_-amine (0.40 g, 2.27 mmol, 1.00 equiv). The reaction mixture was stirred in the dark for a further 60 h. The resulting mixture was then filtered, under an inert atmosphere using a filter cannula, into a flask of vigorously stirring diethyl ether (200 mL) to produce a yellow precipitate. The suspension was continuously agitated with slow stirring and most of the ether was decanted. The yellow solid was washed twice, to remove remaining DMSO, with acetone (60 mL) and diethyl ether (100 mL); stirring was maintained and solvent was decanted each time. Any remaining solvent was then removed in vacuo to obtain the product as a fine yellow powder.

##### 4.1.4.1 Folate-PEG_2_-azide (**6a**)

Following general procedure C, compound **6a** was prepared from folic acid (1.00 g) and azide-PEG_2_-amine, **5a** (0.40 g) to obtain the product as a fine yellow powder (1.02 g, 75%).

^**1**^**H NMR** of a mixture of regioisomers (600 MHz, D_2_O, pH 9) δ 8.62 (s, 1H, ***H1***), 7.74–7.65 (m, 2H, ***H4***), 6.89–6.81 (m, 2H, ***H3***), 4.60 (br. s, 2H, ***H2***), 4.40–4.36 (m, 1H ***H5***), 3.71–3.13 (m, 12H, PEG), 2.48–2.30 (m, 2H, ***H6/H7***), 2.20–2.03 (m, 2H, ***H6/H7***) ppm.

^**13**^**C NMR** (151 MHz, D_2_O) δ 181.7 (***C***OOH), 178.7, 175.7, 174.5, 172.6, 170.1, 169.2, 163.5, 155.5, 151.4, 151.1, 147.8, 147.5 (***C1***), 129.3 (***C4***), 129.2 (***C4***), 129.0, 128.2, 121.5, 120.9, 115.1 (***C3***), 112.8 (***C3***), 112.7, 69.6, 69.51, 69.46, 69.3, 69.2, 69.1, 69.0, 68.9, 68.5, 55.2 (***C5***), 55.1 (***C5***), 50.0, 49.9, 45.8 (***C2***), 39.0, 38.9, 33.9 (***C6***/***C7***), 32.6 (***C6***/***C7***), 27.5 (***C6***/***C7***) ppm.

**ES-MS** (MeOH) *m*/*z* 598.2 [M + H]^+^, 620.2 [M + Na]^+^, LC-MS (MeOH) 597.2 [M + H]^+^, 1193.5 [2M + H]^+^.

##### 4.1.4.2 Folate-PEG_5_-azide (**6b**)

Following general procedure C, compound **6b** was prepared from folic acid (1.30 g) and azide-PEG_5_-amine, **5b** (0.90 g) to obtain the product as a fine yellow powder (1.59 g, 74%). Further purification can be achieved by column chromatography on neutral alumina, dry loaded, in *i*PrOH/H_2_O + 1% [NH_4_]OH (8:2 to 7:3).

The crude was purified by HPLC (S3.4 HPLC conditions 4) using mass directed or UV-directed fraction collection (λ = 280 nm) t_R_ (prep) = 2.4 min.

^**1**^**H NMR** of a mixture of regioisomers (400 MHz, D_2_O with NH_4_OH, pH 9) δ 8.61 (s, 1H, ***H1***), 7.67 (dd, ^3^*J*_*H–H*_ = 8.9, 7.6 Hz, 2H, ***H4***), 6.81 (overlapping d/dd, ^3^*J*_*H–H*_ = 8.9 Hz, 2H, ***H3***), 4.58 (s, 2H, ***H2***), 4.40–4.33 (m, 1H, ***H5***), 3.73–3.32 (m, 23H, PEG), 3.20 (m, 1H, PEG), 2.44–2.29 (m, 2H, ***H6/H7***), 2.27–1.98 (m, 2H, ***H6/H7***) ppm.

^**13**^**C NMR** (151 MHz, D_2_O with NH_4_OH, pH 9) δ 181.62 (***C***OOH), 181.56 (***C***OOH), 178.62 (***C***ONH), 178.55 (***C***ONH), 175.72 (***C***ONH), 175.65 (***C***ONH), 174.50 (***C***ONH), 174.45 (***C***ONH), 171.3 (***C***ONH), 170.2 (***C***ONH), 169.9 (***C***ONH), 169.1 (***C***ONH), 155.2, 151.3, 151.1, 151.0, 150.8, 148.0, 147.9 (***C1***), 147.7 (***C1***), 129.3 (***C4***), 129.2 (***C4***), 129.1 (***C4***), 129.0 (***C4***), 128.0, 122.7, 122.1, 121.4, 120.8, 119.5, 115.10 (***C3***), 115.05 (***C3***), 112.6 (***C3***), 112.5 (***C3***), 69.62 (PEG), 69.59 (PEG), 69.55 (PEG), 69.51 (PEG), 69.47 (PEG), 69.43 (PEG), 69.39 (PEG), 69.3 (PEG), 69.2 (PEG), 69.1 (PEG), 68.91 (PEG), 68.88 (PEG), 68.86 (PEG), 68.7 (PEG), 68.6 (PEG), 55.22 (***C5***), 55.19 (***C5***), 55.04 (***C5***), 54.98 (***C5***), 50.3 (PEG), 50.13 (PEG), 50.08 (PEG), 45.70 (***C2***), 45.67, 39.03, 39.00, 38.9, 33.91 (***C6/C7***), 33.88 (***C6/C7***), 32.7 (***C6/C7***), 32.6 (***C6/C7***), 30.4, 27.84 (***C6/C7***), 27.76 (***C6/C7***), 27.68 (***C6/C7***), 27.66 (***C6/C7***), 23.8 ppm.

**ES-MS** (MeOH) *m*/*z* 730.3 [M + H]^+^, 752.3 [M + Na]^+^, HRMS (ES^+^), found (*m*/*z*): 730.3262 [M + H]^+^; C_31_H_44_O_10_N_11_ requires 730.3267 (|Δ*m*/*z*|= 0.74 ppm).

##### 4.1.4.3 Propargyl-tBu_3_DO3A: 1,4,7-Tri(tert-butoxycarbonylmethyl)–10-(prop-2-ynyl)-1,4,7,10-tetraazacyclododecane (**S5**)

Prepared with minor adaptation from the procedure described by Jauregui et al.^41^ Refer to Scheme S3 for overview. Triester, **2** (4.00 g, 6.72 mmol, 1.00 equiv) was added to dry MeCN (60 mL) with potassium carbonate (2.32g, 16.8 mmol, 2.50 equiv) and stirred for 20 min. The flask was cooled on ice, propargyl bromide (80 wt % in toluene) (0.75 mL, 5.83 mmol, 1.00 equiv) was added dropwise and the reaction was left stirring overnight at 15–25 °C under N_2_. Inorganic solids were removed by filtration and the solvent was removed under reduced pressure. The resulting oil was dissolved in toluene (75 mL) and washed with water (60 mL × 2). The organic layer was dried over MgSO_4_, filtered through Celite pad and the solvent removed to give **S5** as a clear golden oil (2.61 g, 4.72 mmol, 70%).

^**1**^**H NMR** (500 MHz, CDCl_3_) δ 3.42 (s, 2H, C***H***_***2***_C≡C), 3.27 (s, 6H, arm C***H***_***2***_CO_2_), 2.82 (s, 12H, ring C***H***_***2***_), 2.68 (m, 4H, ring C***H***_***2***_), 2.14 (t, *J* = 2.4 Hz, 1H, C≡C***H***), 1.45 (s, 27H, 3 × (C***H***_***3***_)_3_) ppm.

^**13**^**C NMR** (126 MHz, CDCl_3_) δ 171.3 (***C***OO), 80.9 (***C***(CH_3_)_3_), 79.4(***C***≡CH), 72.6(C≡***C***H), 56.9 (***C***H_2_CO arm), 52.3(N***C***H_2_ ring), 52.0 (N***C***H_2_ ring), 51.2 (N***C***H_2_ ring), 43.2 (***C***H_2_C≡CH), 28.37(C(***C***H_3_)_3_), 21.59 ppm.

**ES-MS**(MeOH): *m*/*z* 553.4 [M + H]^+^, 575.4 [M + Na]^+^; HRMS (ES+), found (*m*/*z*): 553.3944 [M + H]^+^; C_29_H_53_O_6_N_4_ requires 553.3960 (|Δ *m*/*z*| = 2.81 ppm).

##### 4.1.4.4 pDO3A:1,4,7-Tris(carboxymethyl)-10-(prop-2-ynyl)-1,4,7,10-tetraazacyclododecane (**7**)

Following general procedure A, compound **7** was prepared from **S5** (0.5 g). The brown solid was further purified and was dissolved in minimal MeOH (∼8 mL) and precipitated upon addition of DCM (∼2 mL), centrifuged and the supernatant discarded to afford the product, 7 as the colorless trifluoroacetate salt (0.178 g, 0.462 mmol, 64%).

^**1**^**H NMR** (500 MHz, D_2_O) δ 3.94–3.71 (m, 6H, C***H***_2_ arm), 3.62 (s, 2H, C***H***_***2***_C≡C), 3.51–3.25 (m, 8H, C***H***_**2**_ ring), 3.25–3.05 (m, 8H, C***H***_***2***_ ring), 2.78 (br. s, 1H, C≡C***H***) ppm.

^**13**^**C NMR** (126 MHz, D_2_O) δ 173.1 (br. s, ***C***=O), 171.0 (br. s, ***C***=O), 76.4 (br. s, ***C***≡CH), 55.5 (br. s), 53.8 (br. s), 50.6 (br. s), 50.3 (br. s), 48.9 (br. s), 48.0 (br. s), 43.0 (br. s) ppm.

**ES-MS**(MeOH): *m*/*z* 385.2 [M + H]^+^; HRMS (ES+), found (*m*/*z*): 385.2080 [M + H]^+^; C_17_H_29_O_6_N_4_ requires 3585.2082 (|Δ *m*/*z*| = 0.48 ppm).

##### 4.1.4.5 Tb**7**

Following general procedure B in MeOH, the Tb**7** was prepared from **7** (0.2 g, 0.4 mmol, 1.00 equiv) and Tb(OTf)_3_(0.25 g, 0.42 mmol, 1.05 equiv). The solvent was removed under reduced pressure and the solid dissolved in 2 mL water. The pH was adjusted to pH 14 to precipitate out excess lanthanide as lanthanide hydroxide. The solution was centrifuged. The supernatant was kept, and the pH was adjusted to pH 7, before removing the solvent. The complexes were dialyzed with Float-a-lyzer with MWCO 500 Da. The solution was lyophilized to yield Tb**7** as a colorless powder (0.24 g, 0.45 mmol, 86%).

^**1**^**H NMR** (400 MHz, D_2_O) δ 414.6, 241.1, 225.5, 186.3, 82.7, 62.1, 51.5, 34.4, – 13.0, – 30.0, – 45.2, – 69.0, – 88.1, – 115.4, – 167.80, – 181.7, – 344.6, – 373.7, – 384.7, – 410.8 ppm. **ES-MS** (MeOH): *m*/*z* 541.2 [M + H]^+^; HRMS (ES^+^) found 541.1105 [M + H^+^]; C_17_H_26_O_6_N_4_^159^Tb requires 541.1100 (|Δ *m*/*z*| = 0.89 ppm). **Luminescence lifetimes:** H_2_O: 1.25 ms, D_2_O: 2.90 ms, *q* = 2.0.

##### 4.1.4.6 Tb.pDO3A Clicked to FA-PEG_2_-N_3_^58^ (Tb**L**^**2**^)

Tb.pDO3A, Tb**7** (0.150 g, 0.28 mmol, 1.00 equiv), FA-PEG_2_-N_3_ (**6a**) (0.185 g, 0.31 mmol, 1.10 equiv) and (CF_3_SO_3_Cu)_2_·C_6_H_6_ (14.2 mg, 0.028 mmol, 0.10 equiv) were combined with MeOH (15 mL) and DMSO (5 mL). The solution was heated at reflux at 80 °C while stirring under Ar for 24 h. Methanol was removed *in vacuo* and the residue was dissolved in water before lyophilization to aid removal of the DMSO. The solid was dissolved in H_2_O (50 mL) and gently stirred with 2–3 g CupriSorb resin for 24 h to remove copper. The insoluble CupriSorb was removed by filtration and the filtrate was dried *in vacuo*. The compound was purified by HPLC (S3.5 HPLC conditions 5) and then dried by lyophilization to afford a yellowy-green powder.

Analytical purity = 85%, t_R_(prep) = 12.62 min; t_R_(ana) = 1.85 min, (18.5 mg, 0.016 mmol, 4%)

^**1**^**H NMR** (500 MHz, D_2_O): δ 318.7, 290.2, 223.6, 220.2, 206.1, 194.7, 179.6, 175.9, 145.5, 116.6, 112.9, 18.5, 14.3, 13.3, 12.0, 11.2, 10.4, – 14.4, – 19.7, – 28.0, – 35.1, – 40.5, – 45.9, – 50.1, – 60.6, – 76.1, – 86.8, – 90.7, – 99.6, – 102.5, – 131.2, – 140.8, – 151.2, – 152.8, – 164.2, – 176.4, – 330.4, – 347.0, – 374.1 ppm. Only major resolved peaks outside the +10 to −10 ppm range are reported.

**ES-MS** (H_2_O) *m*/*z* 1139.7 [M + H]^+^, 1161.8 [M + Na]^+^, HRMS (ES^+^), found (*m*/*z*): 1138.35286 [M + H]^+^; C_42_H_57_O_13_N_15_^159^Tb requires 1138.35082.(|Δ*m*/*z*|= 1.79 ppm); (*m*/*z*): 569.67890 [M + 2H]^2+^; C_42_H_58_O_13_N_15_^159^Tb requires 569.67905 (|Δ*m*/*z*|= 0.26 ppm).

**UV–vis** (H_2_O) λ_max_ = 282 nm.

##### 4.1.4.7 Tb**L**^**3**^

Tb-pDO3A, Tb**7** (0.033 g, 0.062 mmol, 1.00 equiv) and FA-PEG_5_-N_3_, (**6b**) (0.054 g, 0.074 mmol, 1.20 equiv) were dissolved in H_2_O (3.5 mL). To this, CuSO_4_·5H_2_O (1.54 mg, 0.0062 mmol, 0.10 equiv) (62 μL of 0.1 M solution in H_2_O) and sodium ascorbate (6.11 mg, 0.031 mmol, 0.50 equiv) (308 μL of 0.1 M solution in H_2_O) were added and heated at 40 °C under Ar for 25 h monitored by LC-MS. The reaction mixture was diluted in 50 mL H_2_O and gently stirred with 2–3 g CupriSorb resin to remove copper for 24 h. The insoluble CupriSorb was removed by filtration, and the filtrate was dried *in vacuo* to yield the crude as a yellow residue (152 mg, 0.12 mmol, crude 195%).

Tb-pDO3A, Tb**7** (0.031 g, 0.057 mmol, 1.00 equiv) and FA-PEG_5_-N_3_ (**6b**) (0.050 g, 0.069 mmol, 1.20 equiv) were dissolved in H_2_O (5 mL) and degassed with three freeze–pump–thaw cycles. To this, (CuOTf)_2_·C_6_H_6_ (5.0 mg, 0.01 mmol, 0.20 equiv) was added and degassed with a further freeze–pump–thaw cycle and heated at 40 °C under Ar for 3.5 days monitored by LC-MS. The reaction mixture was diluted in 50 mL H_2_O and gently stirred with 2–3 g CupriSorb resin to remove copper for 24 h. The insoluble CupriSorb was removed by filtration, and the filtrate was dried *in vacuo* to yield the crude as a yellow residue (106 mg, 0.083 mmol, crude 146%).

The crude products (258 mg) were combined and purified by HPLC twice. The first HPLC conditions (S3.6 HPLC conditions method 6-purification step 1) isolated the 54.6 mg compound as a mixture of regioisomers from the impurities, t_R_(prep) = 12.34–12.41 min and t_R_(ana) = 2.98 min. The second HPLC conditions separated the major and minor isomers (S3.6 HPLC conditions method 6–purification step 2).

##### 4.1.4.8 Tb**L**^**3 major**^

Analytical purity >99%, t_R_(prep) = 9.71 min, t_R_(ana) = 0.95 min, (26.6 mg, 0.021 mmol, 18%)

^**1**^**H NMR** (500 MHz, D_2_O): δ 322.6, 292.5, 227.0, 222.1, 206.9, 196.1, 181.9, 178.4, 146.4, 118.4, 114.3, 14.6, 12.5, 10.2, −14.1, −19.9, −27.5, −35.2, −41.1, −46.5, −49.9, −60.5, −76.3, −86.8, −90.8, −99.8, −103.5, −132.1, −141.7, −152.0, −154.2, −165.9, −177.9, −333.0, −349.6, −377.7 ppm. Only major resolved peaks outside the +10 to −10 ppm range are reported.

**ES-MS** (H_2_O) (minor isomer) *m*/*z* 1270.38 [M + H]^+^, 635.69 [M + 2H]^2+^; LC-MS (H_2_O) 1266.0 [M – H]^−^, 634.7 [M + 2H]^2+^. HRMS (ES^–^), found (*m*/*z*): 1268.4174 [M + H]^+^; C_48_H_67_O_16_N_15_^159^Tb requires 1268.4149.

##### 4.1.4.9 Tb**L**^**3 minor**^

Analytical purity >9%, t_R_(prep) = 11.02 min, t_R_(ana) = 1.04 min (17.6 mg, 0.014 mmol, 12%)

^**1**^**H NMR** (500 MHz, D_2_O): δ 322.6, 292.6, 227.0, 221.9, 207.0, 196.3, 181.7, 178.2, 146.3, 118.3, 114.2, 14.3, 12.1, −14.3, −20.2, −27.6, −35.4, −41.3, −46.7, −50.0, −60.7, −76.3, −86.8, −90.9, −99.9, −103.6, −132.3, −141.7, −152.3, −154.0, −166.1, −177.8, −332.7, −349.4, −377.6 ppm. Only major resolved peaks outside the +10 to −10 ppm range are reported.

**ES-MS** (H_2_O) (minor isomer) *m*/*z* 1270.38 [M + H]^+^, 635.69 [M + 2H]^2+^; LC-MS (H_2_O) 1267.9 [M + H]^+^, 634.7 [M + 2H]^2+^. HRMS (ES^+^), found (*m*/*z*): 1270.4297 [M + H]^+^; C_48_H_69_O_16_N_15_^159^Tb requires 1270.4295 (|Δ*m*/*z*|= 0.22 ppm).

Tb-1-(4′-ethynyl-2-acetophenone)-4,7,10-tris(carboxymethyl)-1,4,7,10-tetraazacyclododecane clicked to FA-PEG_5_-N_3_ (Tb**L**^**4**^)

Tb-1-(4′-ethynyl-2-acetophenone)-DO3A, Tb**4** (0.0744 g, 0.12 mmol, 1.00 equiv) and FA-PEG_5_-N_3_ (**6b**) (0.101 g, 0.14 mmol, 1.20 equiv) were dissolved in H_2_O/MeOH/DMSO (5.5 mL, 6:4:1). To this, CuSO_4_.5H_2_O (2.88 mg, 0.012 mmol, 0.10 equiv) (115 μL of 0.1 M solution in H_2_O) and sodium ascorbate (11.4 mg, 0.058 mmol, 0.50 equiv) were added and heated at 40 °C under Ar for 2 days monitored by LC-MS. The reaction mixture was concentrated under reduced pressure and then diluted in H_2_O (50 mL) which was removed by lyophilization. The yellow residue was then dissolved in H_2_O (50 mL) and gently stirred with 2–3 g CupriSorb resin to remove copper for 24 h. The insoluble CupriSorb was filtered, and the filtrate was dried *in vacuo* to yield the crude as a yellow residue (0.254 g, 0.2 mmol, crude 173%).

The compound was purified HPLC (S3.7 HPLC conditions method 7) isolated the compound as a mixture of regioisomers from the impurities.

From Tb**4** (50 mg, 0.078 mmol, 1.00 equiv) yielded Tb**L**^**4**^ as yellow powder (41.1 mg, 0.03 mmol, 39%) Analytical purity >97%, t_R_(prep) = 13.4 min, t_R_(ana) = 1.16 min.

^**1**^**H NMR** (500 MHz, D_2_O) (mixture of regioisomers) δ 305.5, 266.4, 257.4, 200.1, 195.8, 176.5, 135.5, 128.0, −18.2, −34.4, −39.8, −53.8, −78.0, −84.9, −95.9, −113.0, −132.3, −142.7, −158.9, −168.6, −185.4, −325.9, −431.3 ppm. Only major resolved peaks outside the +10 to −10 ppm range are reported.

**ES-MS** (MeOH) (mixture of regioisomers) *m*/*z* 687.7 [M + 2H]^2+^; LC-MS (MeOH) 686.6 [M + 2H]^2+^, 1372.5 [M + H]^+^. HRMS (ES^+^), found (*m*/*z*): 1374.4548 [M + H]^+^; C_55_H_73_O_17_N_15_^159^Tb requires 1374.4557 (|Δ*m*/*z*|= 0.64 ppm). HRMS (ES^–^), found (*m*/*z*): 1372.4437 [M – H]^−^; C_55_H_71_O_17_N_15_^159^Tb requires 1372.4411.

DOTA-DBCO/1,4,7,10-Tetraazacyclododecane-1,4,7-tris(acetic acid)–10-[3-oxo-3-(5-azadibenzocyclootyne)acetamide] (**S6**)

Prepared with minor adaptation from the procedure described by Zeng et al.^42^ and Liang et al.^43^ DBCO-amine (40.0 mg, 144 μmol, 1.00 equiv), trimethylamine (224 μL, 1.6 mmol, ∼ 11.0 equiv) and dry DMF (8 mL) were added to a flame-dried flask and stirred for 10 min. DOTA-NHS ester (140 mg, 184 μmol, 1.27 equiv) was added and stirred at room temperature overnight. Water (4 mL) was added to the reaction mixture to hydrolyze excess DOTA-NHS ester. The solvents were removed in vacuo to yield a pale yellow transparent oil. The residue was dissolved in 3.5 mL (8 H2O: 2 MeCN) and purified by preparative HPLC (S3.8 HPLC conditions method 8). UV-directed fraction collection λ = 220 nm. t_R_(prep) = 4.1 min. Solvent was removed in vacuo to yield the **S6** as an off-white solid (66 mg, 69%).

^**1**^**H NMR** (600 MHz, 8 D_2_O: 2 CD_3_CN) δ 8.56 (br. s/m, 1H, N***H***), 8.00 (d, ^3^*J*_*H–H*_ = 7.5 Hz, 1H, Ar***H***), 7.86–7.71 (m, 6H, Ar***H***), 7.68 (d, ^3^*J*_*H–H*_ = 7.5 Hz, 1H, Ar***H***), 5.42 (d, ^2^*J*_*H–H*_ = 14.2 Hz, 1H, ***H3***), 4.13–3.92 (overlapping d/m, 5H, ***H3***, C***H***_***2***-arm_/C***H***_***2***-ring_), 3.85 (s, 2H), 3.77–3.19 (m, 20H, C***H***_***2***-arm_/C***H***_***2***-ring_,, 2 × ***H1***), 2.77 (dt, ^2^*J*_*H–H*_ = 16.0 Hz, ^3^*J*_*H–H*_ = 6.2 Hz, 1H, ***H2***), 2.44 (dt, ^2^*J*_*H–H*_ = 15.9 Hz, ^3^*J*_*H–H*_ = 7.2 Hz, 1H, ***H2***) ppm.

^**13**^**C NMR** (151 MHz, 8 D_2_O: 2 CD_3_CN) δ 172.3 (***C***OOH), 165.3 (***C***OOH), 150.1 (***C***ON), 147.3 (***C***ON), 131.5 (Ar***C***), 128.71 (Ar***C***), 128.69 (Ar***C***), 128.3 (Ar***C***), 128.0 (Ar***C***), 127.6 (Ar***C***), 126.4 (Ar***C***), 125.1 (Ar***C***), 121.8 (***C4/5/8/9***), 120.9 (***C4/5/8/9***), 113.7 (***C4/5/8/9***), 107.2 (***C4/5/8/9***), 55.5 (br. s, ring/arm), 54.9 (***C3***), 54.8 (***C6/7***), 53.6 (ring/arm), 52.7 (br. s, ring/arm), 50.3 (br. s, ring/arm), 50.0 (br. s, ring/arm), 47.6 (br. s, ring/arm), 34.89 (ring/arm), 34.84 (***C1***), 33.2 (***C2***) ppm.

**ES-MS** (MeOH): *m*/*z* 664.2 [M + H]^+^. HRMS (ES+): found (*m*/*z*): 663.3134 [M + H]+; C_34_H_43_O_8_N_6_ requires 663.3137 (|Δ*m*/*z*| = 0.41 ppm).

##### 4.1.4.10 Tb**S6**

To a solution of the ligand **S6** (0.040 g, 0.06 mmol, 1.00 equiv) in 3 mL (H_2_O/MeCN, 7:3), the Tb(OTf)_3_ (0.038 g. 0.063 mmol, 1.05 equiv) was added and the reaction mixture was stirred at 50 °C for 30 min. The pH was adjusted to 4/5 by dropwise addition of an aqueous 1 M NaOH solution. The reaction was left to stir at 50 °C for 48 h. The solvent was removed under reduced pressure and the residue was suspended in water. The pH of this solution was adjusted to 7 by addition of aqueous 1 M NaOH and the solvent was removed under reduced pressure giving the crude as a pale pink powder. The pale pink powder was purified by column chromatography (alumina MeCN/H_2_O, 8:2 to 7:3) to yield Tb**S6** as an off-white powder (75.7 mg, 0.092 mmol, 154%). *R*_f_ = 0.32 (MeCN/H_2_O, 8:2). Excess mass attributed to salts; product used with salts.

^**1**^**H NMR** (400 MHz, D_2_O) δ 251.6, 202.4, 191.6, 172.7, 144.2, 117.4, 111.6, 110.0, 73.6, 59.9, 46.0, 42.6, 25.2, 22.1, 21.5, 20.5, 19.7, 18.1, 17.0, 16.1, 14.5, 14.0, 13.5, 13.1, 12.5, 12.0, 11.1, −26.1, 42.3, −67.7, −79.4, −100.4, −112.8, −127.0, −205.2, −373.7, −379.6, −382.1, −384.8, −398.7, −402.4 ppm.

**ES-MS** (MeOH): *m*/*z* 819.1 [M + H]^+^, 841.1 [M + Na]^+^.HRMS (ES+): found (*m*/*z*): 819.2154 [M + H]^+^; C_34_H_40_O_8_N_61_^59^Tb requires 819.2156 (|Δ*m*/*z*| = 0.20 ppm).

##### 4.1.4.11 Tb-DOTA-DBCO Clicked to FA-PEG_5_-N_3_ (Tb**L**^**5**^)

Crude Tb-DOTA-DBCO, Tb**S6** (59 mg, 0.05 mmol, 1.06 equiv) was dissolved in H_2_O/MeCN (2 mL, 8:2). To this, FA-PEG_5_-N_3_, **6b** (35 mg, 0.047 mmol, 1.00 equiv) was added and the reaction was stirred at 15–25 °C for 24 h. The reaction mixture was concentrated under reduced pressure and the residue dissolved in H_2_O (8 mL) and removed by lyophilization. The crude was purified by HPLC (S3.9 HPLC conditions method 9). (33.7 mg, 0.022 mmol, 44%) Analytical purity >97% t_R_(prep) = 14.1 min, t_R_(ana) = 1.5 min.

^**1**^**H NMR** (500 MHz, D_2_O) δ 256.0, 241.6, 202.8, 194.7, 111.4, 105.4, 65.7, 48.9, 45.7, 26.0, 19.7, 16.5, 15.5, 14.0, 13.0, 12.1, 11.9, 10.6, −46.0, −54.8, −67.0, −76.5, −78.7, −101.5, −110.4, −114.3, −125.0, −197.5, −211.4, −365.5, −374.5, −393.6 ppm.

**ES-MS** (H_2_O/MeCN (8:2)) (mixture of regioisomers) *m*/*z* 1548.4 [M + H]^+^, 775.2 [M + 2H]^2+^; HRMS (ES^+^), found (*m*/*z*): 1570.5191 [M + Na]^+^; C_65_H_82_O_18_N_17_^159^TbNa requires 1570.5169 (|Δ*m*/*z*|= 1.40 ppm).

### 4.2 Folate Receptor Affinities/Folate-FITC Competition

Human KB nasopharyngeal epidermoid carcinoma cells (expressing FRα) and FRβ-transfected Chinese Hamster Ovary cells were cultured in Folate-free RPMI medium (Thermo Fisher Scientific) supplemented with 20% Newborn Calf Serum, 1% antibiotics (penicillin and streptomycin) and 25 mM HEPES, in a humidified 37 °C incubator with 5% CO_2_ as described by Van der Heijden et al.^4^ The culture medium was refreshed every 3 days. Stock solutions (1 mM) of Tb-folate conjugates were made by dissolving in 50 mM NaHCO_3_ and stored at −20 °C until use. Assessment of relative binding affinities of FR for Tb-folate compounds was performed by flow cytometry assays in competition with Folate-FITC as described previously.^59,60^ For KB cell experiments (25,000 cells/100 μL), Tb-folate compounds were diluted in PBS to reach the final concentration: 10, 25, 50, 75, 100, 250, 500, 1000 nM in the presence of 10 nM Folate-FITC. For CHO/FRβ cell experiments (25 000 cells/100 μL), Tb-folate compounds were diluted in PBS to reach the final concentration: 1, 2.5, 5, 7.5, 10, 25, 50, 100 nM in the presence of 1 nM Folate-FITC. As control competitor, folic acid was diluted in PBS, and added, in separate experiments, to reach the same concentration range as Tb-folate compounds. Cells treated with compounds were incubated on ice, in the dark for 10 min. Treated cells were then washed twice with ice-cold PBS by centrifugation (600 RCF for 5 min). Treated cells were kept on ice until the acquisition. Attune NxT flow cytometer (Thermo Fisher Scientific) was used for data acquisition. Flow cytometry data was analyzed in FlowJo v10.8 Software (BD Life Sciences). Statistical analysis was done in GraphPad Prism version 6.0.0 for Windows, GraphPad Software, San Diego, California USA. Data from 6–9 replicative experiments were analyzed. Example output of the competition assays is presented in SI section 7.

Considering FRα and FRβ share the same affinity for folic acid and Folate-FITC, relative affinities for Tb-folate compounds were expressed relative to folic acid.^59,60^ The relative affinity was calculated using
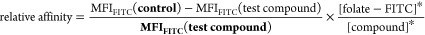
MFI: Mean fluorescent intensity; * concentration in nM.

### 4.3 *In Silico* Assessment

#### 4.3.1 Membrane Permeability

Accurate prediction of membrane permeability is crucial for understanding drug absorption and distribution within the body. In this study, we utilized an improved computational method for estimating membrane permeability based on a physical model that incorporates multiple factors contributing to the free energy of desolvation and state changes, including neutralization and tautomerization upon membrane entry. The model accounts for the differences in dielectric properties between the low-dielectric continuum of the membrane and the high-dielectric continuum of water.^50,51^

First, we looked at the properties of the DOTA fragment and then replaced terbium with calcium, as both calcium and terbium have a coordination number of 8 and are hard metal ions. Additionally, both have two valence electrons in their outermost shell. This similarity in the number of valence electrons can affect their chemical behavior. In silico prediction of the properties with Tb requires more computational power and time, therefore, we simplify the model to increase the speed of the *in silico* prediction (Table 2).

In the first step, QikProp, an absorption, distribution, metabolism, and excretion (ADME), prediction tool developed by Professor William L. Jorgensen was used.^61^ As an input, it takes the 2D structure of the compounds. It prepares the structure by assigning appropriate bond orders, atom types, and protonation states. QikProp computes a range of molecular descriptors that capture various physicochemical properties of the compound. These descriptors include molecular weight, hydrogen bond donors and acceptors, lipophilicity (LogP), polar surface area (PSA), and many others utilizing specific rules. These rules are based on desirable ranges and thresholds for specific properties, such as molecular weight Lipinski’s rule of five criteria,^52^ and Jorgensen’s rule of three.^62^ (SI section 6).

To assess the permeability of the compounds and their ability to cross the blood-brain barrier we looked at values QPPMDCK1 and QPPCaco2. As a comparison, ChEBI_27470 (folic acid) and Glycerol were used (Table 2). We excluded the DOTA part from the prediction model as the algorithm used in the program is not parametrized for such large molecules. There is a limit on the number of atoms. In addition, the DOTA molecule itself is characterized by low permeability. As the “tails” are the main differences between the compounds, we focused on the behavior of the “tails”.

## Supporting Information Available

^1^H NMR spectra, HPLC conditions and folate-FITC competition assay and molecular formula SMILES (PDF)
Molecular formula SMILES (CSV)

## Abbreviations

ADME: absorption, distribution, metabolism and excretion
CHO: Chinese hamster ovary cell
CuAAC: copper-catalyzed azide–alkyne cycloaddition
DCC: *N,N′-*Dicyclohexylcarbodiimide
DBCO: dibenzocyclooctyne
DO3A: 1,4,7,10-tetraazacyclododecane-1,4,7-triacetic acid
DOTA: 1,4,7,10-tetraazacyclododecane-1,4,7,10-tetraacetic acid
FA: folic acid
FITC: fluorescein isothiocyanate
FR: folate receptor
GPI: glycosylphosphatidylinositol
HPLC: high-performance liquid chromatography
MDCK: Madin-Darby canine kidney cells
MFI: mean fluorescence intensity
MRI: magnetic resonance imaging
NIR: near-infrared
OI: optical imaging
pDO3A: propargyl DO3A
PEG: polyethylene glycol
PET: positron emission tomography
PLIM: phosphorescence lifetime imaging microscopy
PSA: polar surface area
RRCK: Ralch Russ canine kidney cell
SPAAC: strain-promoted azide–alkyne cycloaddition
SPECT: single-photon emission computed tomography
TLC: thin layer chromatography
RA: rheumatoid arthritis
RP: reverse phase
UV: ultraviolet
