# Supplementary material for: Synthesis and Characterization of Folic Acid-Conjugated Terbium Complexes as Luminescent Probes for Targeting Folate Receptor-Expressing Cells
Source: J Med Chem. 2024 Aug 14;67(16):14062–76. doi: 10.1021/acs.jmedchem.4c00919 (PMC11345839; doi:10.1021/acs.jmedchem.4c00919)
Supplement: Supplementary file 1 — jm4c00919_si_001.pdf [file jm4c00919_si_001.pdf]

Supporting Information

Synthesis and characterization of folic acid-conjugated terbium complexes as luminescent probes for targeting folate receptor expressing cells

Grace T. McMullon[a#], Aiarpi Ezdoglian[b#], Anna C. Booth[a], Pilar Jimenez-Royo[c], Philip S. Murphy[c], Gerrit Jansen[b], Conny J. van der Laken[b], Stephen Faulkner[a*]

[a] Chemistry Research Laboratory, University of Oxford, Oxford, OX1 3TA United Kingdom

[b] Department of Rheumatology and Clinical Immunology, Amsterdam University Medical Center, Location VU University Medical Center, 1081 HV Amsterdam, Netherlands

[c] GlaxoSmithKline, Gunnels Wood Road, Stevenage, Hertfordshire, SG1 2NY, United Kingdom
[#] Shared first authorship

*Corresponding author, Email: Stephen.faulkner@keble.ox.ac.uk

Contents

1. **Photophysical characterisation**......2
2. **Overview of synthesis of Tb complexes**......3
3. **HPLC purification conditions and traces**......9
4. **HPLC traces of complexes:**......19
5. **^{1}H NMR spectra**......24
6. ***In silico* assessment of permeabilization properties**......38
7. **Folate-FITC competition assay**......40
8. **References**......41

1. Photophysical characterisation

1.1. Luminescence lifetimes

Lifetimes were recorded 0.05 ms after pulsed excitations at the excitation maxima (λ_{ex}) by measuring the decay of the lanthanide main emission peak (Tb 545 nm). The increments after the initial delay were adjusted between 0.2–10 μs depending on the lifetime to have a good sampling of the decay. The obtained data were fitted by mono and double exponential decay models in OriginPro 9, and the most reliable value was chosen according to the adjusted R^2 value and the shape of the residuals. A relative error of 10% is typically found among a series of measurements on the same sample.

The inner sphere hydration numbers (*q*) of Tb^{3+} complexes are calculated by measuring the luminescence lifetimes in both H_2O and D_2O. The q value was calculated from Equation 1 for Tb^{3+} complexes.[1, 2]

$q_{Tb} = 5.0\left(k_{H_2O} - k_{D_2O} - 0.06\right)$ (Equation S1)

where: q_{Tb} is the inner sphere hydration number, *k* is rate constant for luminescence decay.

1.2. Quantum yield measurements

Quantum yields were measured at room temperature and relative to quinine sulfate (QS) in H_2SO_4 0.05 M, $\Phi_{QS} = 0.55$.[3] Quantum yields were calculated according to Equation 2, where 's' refers to the sample and 'ref' refers to the reference standard. *Φ* is the quantum yield, *I* the integrated corrected emission intensity of the emission spectrum, *A* is the absorbance at the excitation wavelength (A = ~ 0.1) and *n* is the refractive index of the solvent. The corrected emission spectra of the sample and reference standard were then measured under the same conditions over the 380–580 nm spectral range. The quantum yields were then calculated according to (2).

$\Phi_s = \left(\frac{I_s}{I_{ref}}\right) \times \left(\frac{A_s}{A_{ref}}\right) \times \left(\frac{n_s}{n_{ref}}\right)^2 \times \Phi_{ref}$ (Equation S2).

2. Overview of synthesis of Tb complexes

2.1. Synthesis of Tb complexes and precursors

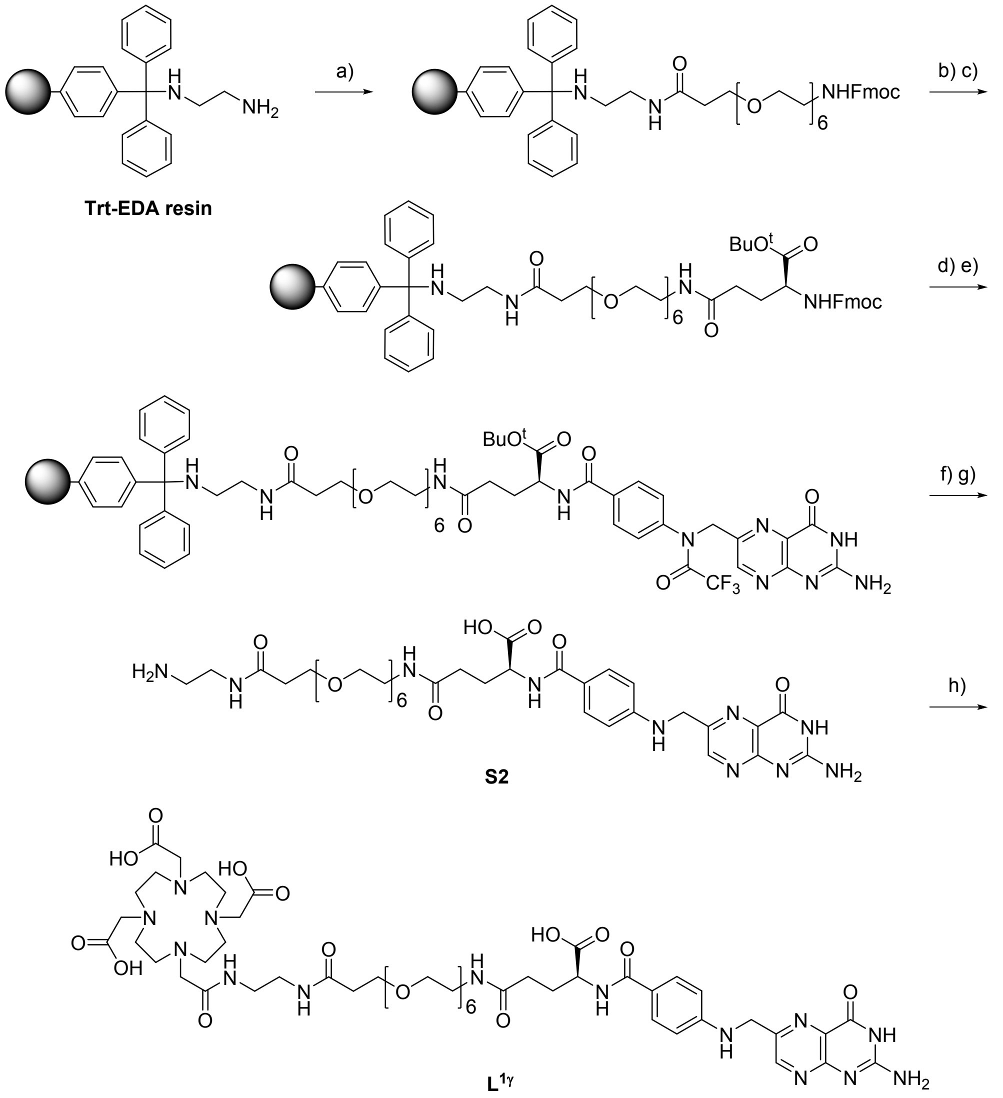

Scheme S1. Synthesis of $L^{1\alpha}$ and $L^{1\gamma}$. Only $L^{1\gamma}$ shown, $L^{1\alpha}$ uses Fmoc-Glu(OtBu)-OH (CAS: 71989-18-9).Conditions and reagents: a) Fmoc-NH-(PEG)$_6$-COOH, HATU, DIPEA, DMF, 20 °C; b) 20% piperidine in DMF, 20 °C, 30 mins; c) Fmoc-Glu-OtBu (CAS: CAS: 84793-07-7) , HATU, DIPEA, DMF, 20 °C; d) 20% piperidine in DMF, 20 °C, 30 mins; e) N_{10} - (TFA)-pteroic acid, HATU, DIPEA, DMF; f) TFA: H_2O: TIPS (95: 2.5:2.5), 20 °C, 3 hrs g) Saturated Na_2CO_3, 20 °C, 30 mins, $L^{1\gamma}$ = 19%; $L^{1\alpha}$= 5%; h) DOTA-NHS ester, DMSO, DIPEA, 20 °C, Ar, 16 h.

N^{10}-(TFA)-pteroic acid (S1)

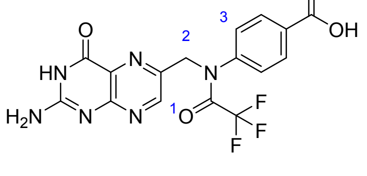

FA-PEG$_6$-EDA-NH$_2$ (S2α or S2γ)

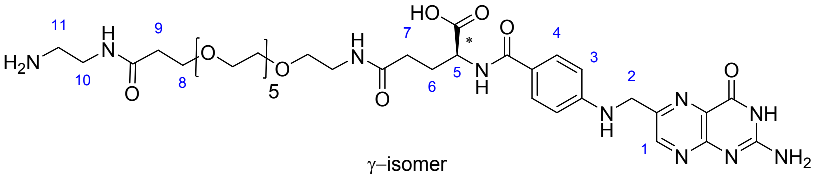

γ–isomer

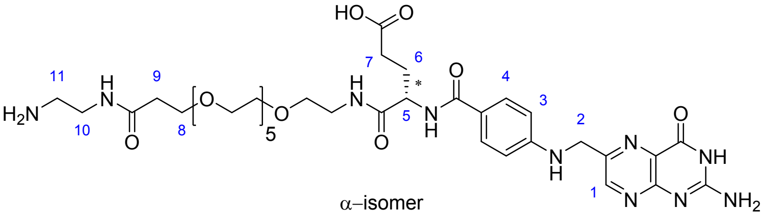

α–isomer

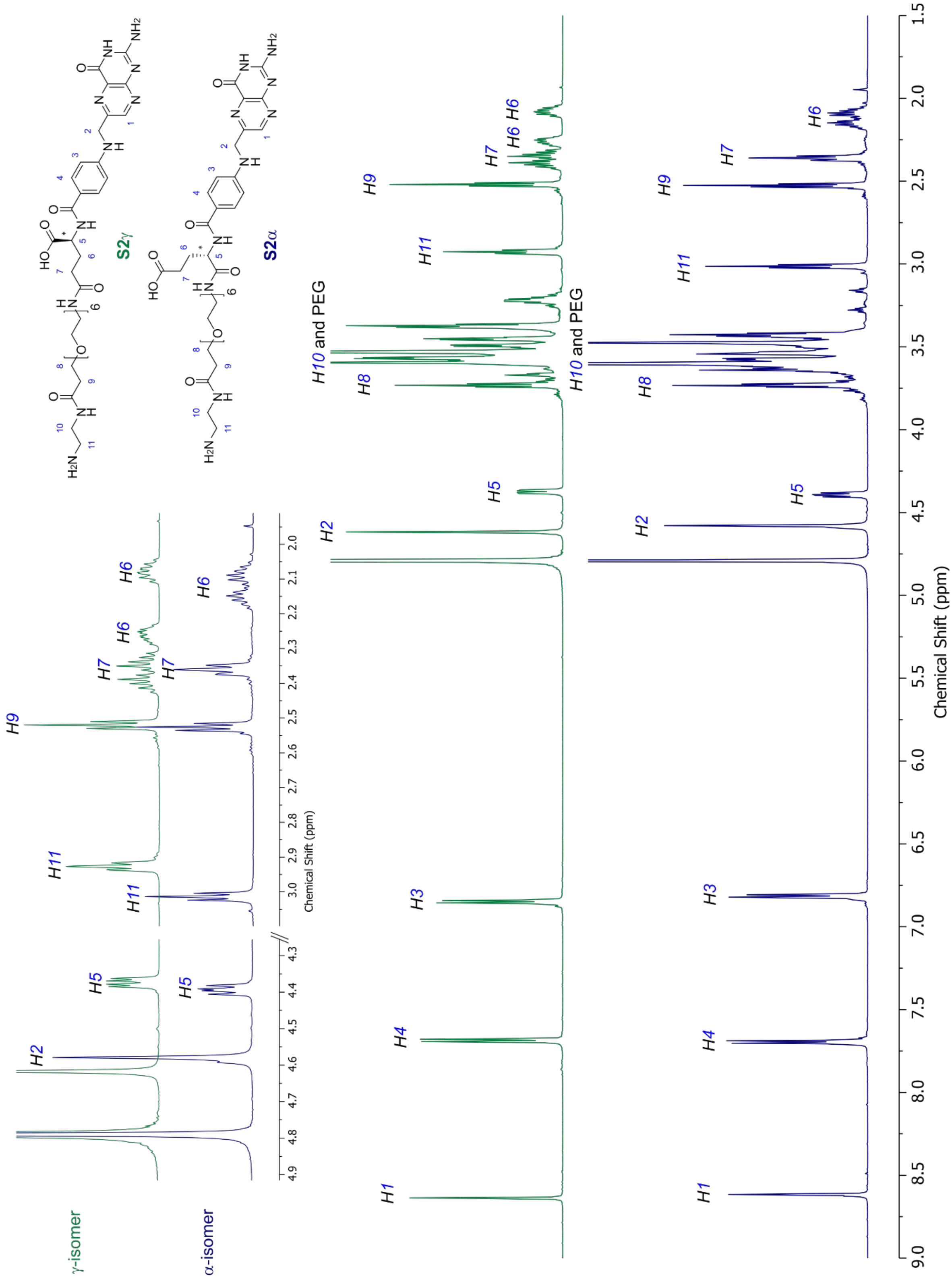

Figure S1. ^{1}H NMR (600 MHz, D_2O) spectra of γ- and α-regioisomers of FA-PEG$_6$-EDA, S2, highlighting key differences between the two isomers.

Folate-PEG$_6$-DOTA (L$^{1\alpha\gamma}$)

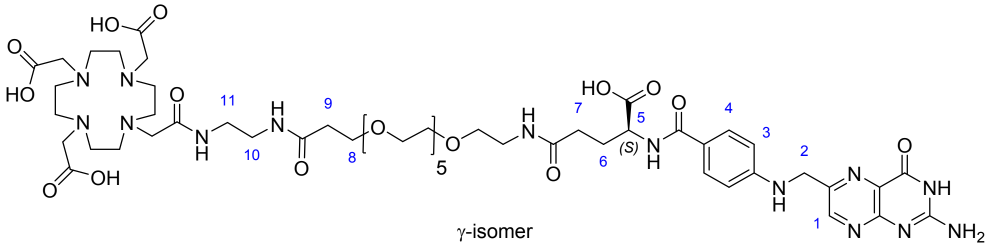

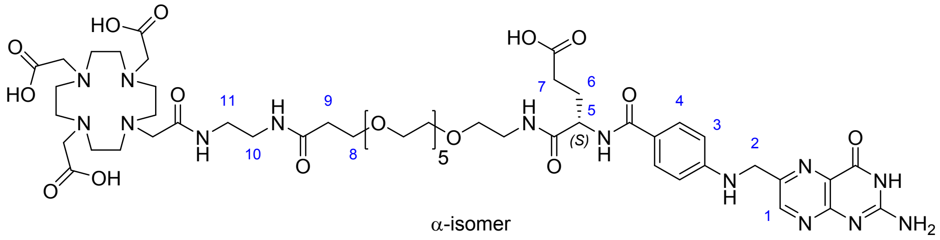

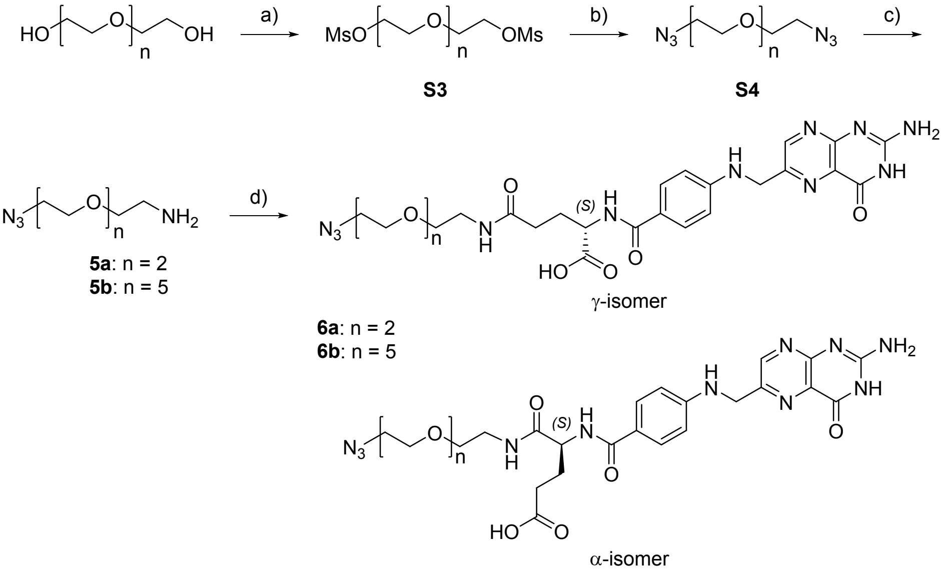

*Scheme S2. Synthesis of **6a** and **6b**. Reagents and conditions: a) MsCl, NEt_3, DMC, 0 °C-20 °C, 4 h, 97%; b) NaN_3, DMF, 65 °C, 16 h, 65%; c) 5% HCl, 0.9 eq. PPh_3 in Et_2O, 20 °C, 24 h, 68%; d) folic acid, DCC, pyridine, DMSO, 20 °C.*

Folate-PEG$_2$-azide (6a)

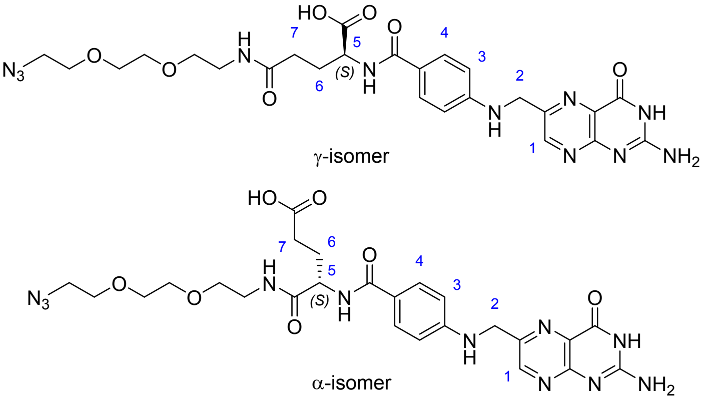

Folate-PEG$_5$-azide (6b)

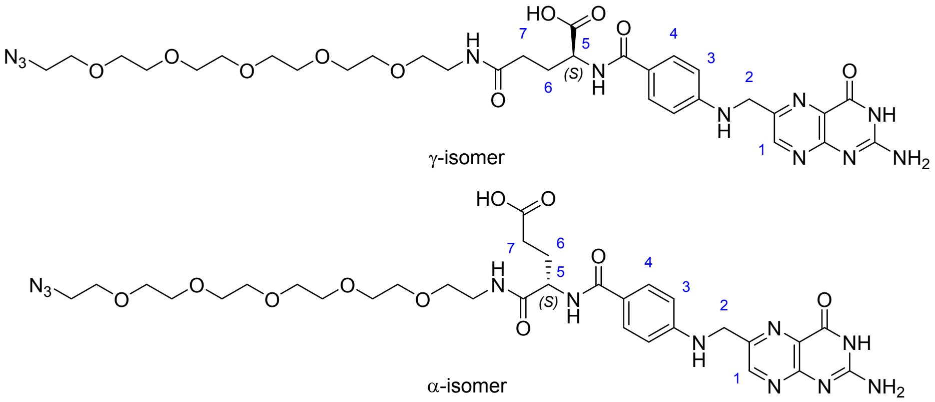

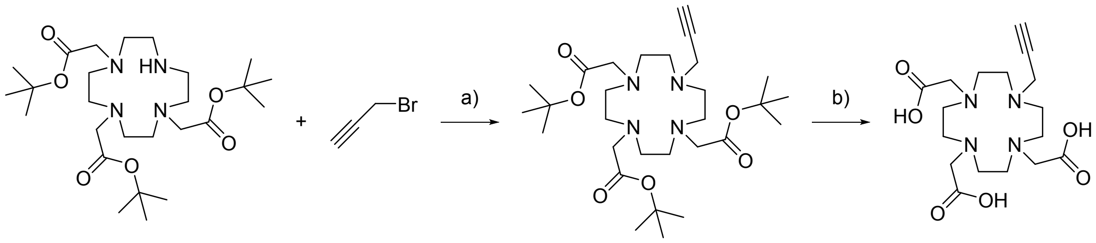

2 S5 **7**

Scheme S3. Synthesis of pDO3A, 7. Reagents and conditions: a) Anh. MeCN, K_2CO_3 (2.5 eq), 0 → 20 °C, 16 h, 70% b) TFA:DCM (1:1), 20 °C, 16 h, 64%.

DOTA-dibenzocyclooctyne (DBCO)/ 1,4,7,10-Tetraazacyclododecane-1,4,7-tris(acetic acid)-10-[3-oxo-3-(5-azadibenzocyclootyne)acetamide] (S6)

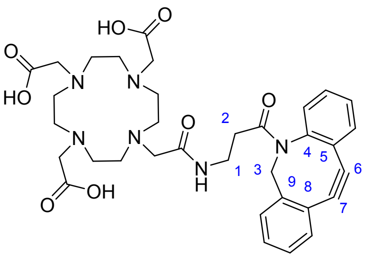

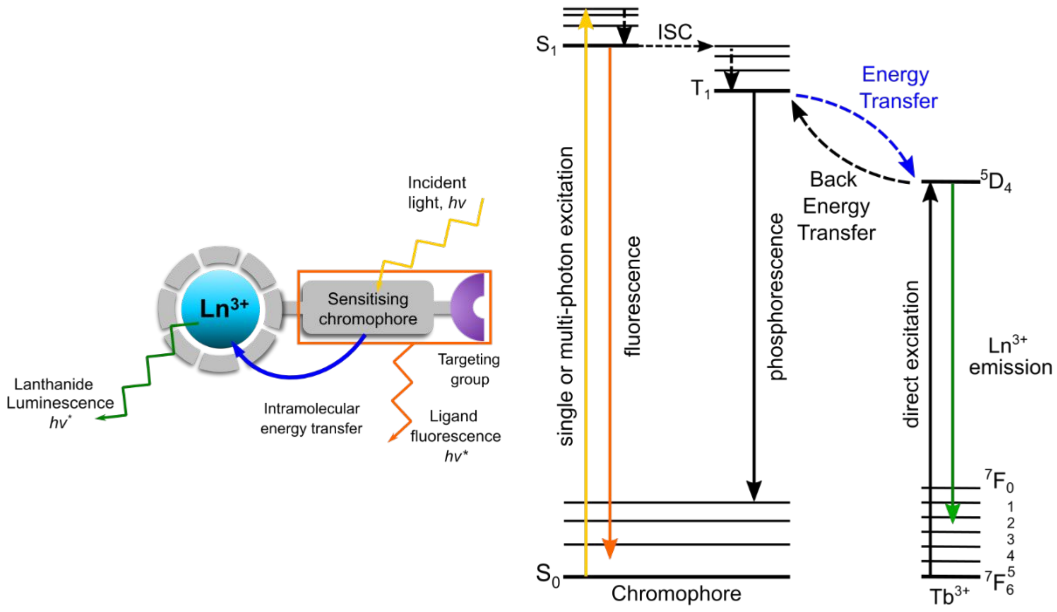

Figure S2. Cartoon depiction of indirect lanthanide excitation via a chromophore (left). Simplified Jablonski diagram showing some pathways for energy transfer for Tb^{3+}(Right). ISC: Intersystem crossing; ET: Energy transfer; BET: Back energy transfer

3. HPLC purification conditions and traces

HPLC purification was carried out for methods 1 and 4 by G. McMullon at the University of Oxford and all other HPLC purification methods were carried out at GSK by Daniel C. Gardener, Andrew Hobbs and Richard Briers.

3.1. HPLC conditions method 1

Instrument name: Waters or Agilent

Column: XBridge BEH C18 OBD Prep Column, 130Å (5 µm, 19 mm ×100 mm)

Mobile phase A: H_2O + 50 µL/L NH_4OH;

Mobile phase B: MeCN + 50 µL/L NH_4OH

Injection Volume: varied volume, typically 750 µL

Collection Mode: UV (DAD) (Agilent/Waters) and/or MS(+/-) (Waters)

Time(min)	A%	B%	Flow rate (mL/min)
0.0	95	5	25
2.0	95	5	25
12.0	60	40	25
13.5	5	95	25
14.0	5	95	25
14.5	95	5	25
15.0	95	5	25

3.2. HPLC conditions method 2

62 mg of sample was dissolved in 4.53 mL DMSO.

Instrument Name: Waters 'Silver' MDAP

Column: XBridge BEH C18 OBD Prep Column, (5µm, 150 x 30 mm)

Mobile phase A: H_2O + 0.1% formic acid

Mobile phase B: MeOH

Injection Volume: varied volume, typically 700 µL

Collection Mode: UV (DAD) and/or MS(+/-)

The gradient was delivered by two pumps in order to use at-column dilution when injecting the sample. The gradient program used for the purification is summarised below:

Chromatographic Pump			Regeneration Pump			
**Time (mins)**	**%B**	**Flow Rate (mL/min)**	**Time (mins)**	**%B**	**Flow Rate (mL/min)**	**Total flow rate (mL/min)**
0.0	15	4	0.0	10	36	40
4.0	15	4	4.0	10	36	40
4.5	15	20	4.5	15	20	40
16.0	45	20	16.0	45	20	40
16.5	60	20	16.5	60	20	40
16.6	100	20	16.6	100	20	40
19.0	100	20	19.0	100	20	40

Analytical Method Details

Instrument Name: Shimadzu 'Achiral Toolbox' UHPLC-MS

Column: BEH C18 (1.7µm, 30 mm × 2.1 mm)

Mobile phase A: H_2O + 0.1% Formic acid

Mobile phase B: MeOH

Temperature (°C): 30

Sample Diluent: DMSO

Injection Volume (µL): 0.5

Time (mins)	%A	%B	Flow rate (mL/min)
0.00	99	1	0.8
2.35	5	95	0.8
2.75	5	95	0.8
2.85	5	95	0.8
3.50	99	1	0.8

3.3. HPLC conditions method 3

85 mg of sample was dissolved in 4.53 mL DMSO.

Instrument Name: Waters 'Silver' MDAP

Column: XBridge BEH C18 OBD Prep Column, (5µm, 150 x 30 mm)

Mobile phase A: H_2O + 0.1% v/v formic acid

Mobile phase B: MeOH

Injection Volume: varied volume, typically 500 µL

The gradient was delivered by two pumps in order to use at-column dilution when injecting the sample.

The gradient program used for the purification is summarised below:

Chromatographic Pump			Regeneration Pump			
**Time (mins)**	**%B**	**Flow Rate (mL/min)**	**Time (mins)**	**%B**	**Flow Rate (mL/min)**	**Total Flow rate (mL/min)**
0.0	15	4	0.0	5	36	40
4.0	15	4	4.0	5	36	40
4.5	15	20	4.5	15	20	40
16.0	45	20	16.0	45	20	40
16.5	60	20	16.5	60	20	40
16.6	100	20	16.6	100	20	40
19.0	100	20	19.0	100	20	40

Analytical Method Details

Instrument Name: Shimadzu 'Achiral Toolbox' UHPLC-MS

Column: BEH C18 (1.7 µm, 30 mm × 2.1 mm)

Mobile phase A: H_2O + 0.1% v/v formic acid

Mobile phase B: MeOH

Temperature (°C): 30

Sample Diluent: DMSO

Injection Volume (µL): 0.5

Time (mins)	%A	%B	Flow Rate (mL/min)
0.00	1	1	0.8
2.35	95	95	0.8
2.75	95	95	0.8
2.85	95	95	0.8
3.50	1	1	0.8

3.4. HPLC conditions method 4

Instruments: Waters or Agilent

Column: XBridge BEH C18 OBD Prep Column, 130Å (5 µm, 19 mm ×100 mm)

Mobile phase A: H_2O + 50 µL/L NH_4OH

Mobile phase B: MeCN + 50 µL/L NH_4OH

Collection Mode: UV (DAD) (Agilent) and/or MS(+/-) (Water)

Time (min)	A %	B %	Flow (mL/min)
0.0	95	5	25
5.0	50	50	25
5.5	5	95	25
6.5	5	95	25
9.0	95	5	25
10.0	95	5	25

3.5. HPLC conditions method 5

120 mg of sample was dissolved in 11.7 mL DMSO: water: MeOH (1:1:1)

Instrument name: Waters ‘Silver’ MDAP

Column: Zorbax SB-Phenyl (5 µm, 150 mm × 30 mm)

Mobile phase A: H_2O + 0.1% v/v TFA acid

Mobile phase B: MeOH

Injection Volume: varied volume, typically 2000 µL

Collection Mode: Fractionation by MS(+/-)

The gradient was delivered by two pumps in order to use at-column dilution when injecting the sample. The gradient program used for the program is summarised below:

Chromatographic Pump:			Regeneration Pump:			
**Time (mins)**	**%B**	**Flow rate (ml/min)**	**Time (mins)**	**%B**	**Flow rate (mL/min)**	**Total Flow rate (mL/min)**
0.0	30	4	0.0	20	36	40
4.0	30	4	4.0	20	36	40
4.5	30	20	4.5	30	20	40
16.0	55	20	16.0	55	20	40
16.5	80	20	16.5	80	20	40
16.6	100	20	16.6	100	20	40
19.0	100	20	19.0	100	20	40

Analytical Method Details

Instrument name: Shimadzu ‘Achiral Toolbox’ UHPLC-MS

Column: Zorbax Phenyl (3.5 µm, 30 mm × 2.1 mm)

Mobile phase A: H_2O + 0.1% v/v TFA acid

Mobile phase B: MeOH

Temperature (°C): 30

Sample diluent: DMSO

Injection volume (µL): 0.5

Flow rate (mL/min): 1.1 (raised 1.8 between 4.71 and 5.39 min)

Time (mins)	%A	%B
0.00	100	0
4.70	0	100
5.40	0	100
5.45	100	0
5.60	100	0

3.6. HPLC conditions method 6

Purification step 1:

156 mg of sample (from $CuSO_4 \cdot 5H_2O$) was dissolved in 7 mL DMSO.

107 mg of sample (from $(CuOTf)_2 \cdot C_6H_6$) was dissolved in 5 mL DMSO.

Instrument Name: Waters 'Platinum' MDAP

Column: CSH C18 (5µm, 150 × 30 mm)

Mobile Phase A: H_2O + 0.1% v/v formic acid

Mobile Phase B: MeCN + 0.1% v/v formic Acid

Injection Volume: 1500 µL

Collection Mode: Fractionation by MS(+)

The gradient was delivered by two pumps in order to use at-column dilution when injecting the sample. The gradient program used for the purification is summarised below:

**Chromatographic Pump:**			**Regeneration Pump:**			
**Time (mins)**	**%B**	**Flow rate (ml/min)**	**Time (mins)**	**%B**	**Flow rate (mL/min)**	**Total Flow rate (mL/min)**
0.0	3	4	0.0	0	36	40
4.0	3	4	4.0	0	36	40
4.5	3	20	4.5	3	20	40
16.0	30	20	16.0	30	20	40
16.5	75	20	16.5	75	20	40
16.6	100	20	16.6	100	20	40
19.0	100	20	19.0	100	20	40

Analytical Method Details

Instrument Name: Waters 'Purple' XEVO UPLC-QTOF-MS

Column: Acquity UPLC CSH C18 (1.7µm, 100 mm × 2.1 mm)

Mobile phase A: H_2O + 0.1% v/v formic acid

Mobile phase B: MeCN + 0.1% v/v formic Acid

Temperature (°C): 50

Sample Diluent: DMSO

Injection Volume (µL): 0.2

**Times (mins)**	**%A**	**%B**	**Flow rate (mL/min)**
0	97	3	0.8
0.2	97	3	0.8
18.0	0	100	0.8
19.0	5	95	0.8
20.0	97	3	0.8

Purification Step 2:

54.6 mg of sample was dissolved in 3 mL DMSO.

Instrument Name: Waters 'Platinum' MDAP

Column: CSH Fluoro-Phenyl (5µm, 150 × 30 mm)

Mobile phase A: H_2O + 0.1% v/v formic acid

Mobile phase B: MeCN + 0.1% v/v formic Acid

Injection Volume: 720 µL

The gradient was delivered by two pumps in order to use at-column dilution when injecting the sample. The gradient program used for the purification is summarised below:

**Chromatographic Pump:**			**Regeneration Pump:**			
**Time (mins)**	**%B**	**Flow rate (mL/min)**	**Time (mins)**	**%B**	**Flow rate (mL/min)**	**Total Flow rate (mL/min)**
0.0	10	4	0	3	36	40
4.0	10	4	4	3	36	40
4.5	10	20	4.5	10	20	40
16.0	40	20	16	40	20	40
16.5	75	20	16.5	75	20	40
16.6	100	20	16.6	100	20	40
19.0	100	20	19	100	20	40

Analytical Method Details

Instrument Name: Shimadzu 'Achiral Toolbox' UHPLC-MS

Column: CSH Fluorophenyl (1.7 µm, 50 mm × 2.1 mm)

Temperature (°C): 30

Mobile phase A: H_2O + 0.1% v/v formic acid

Mobile phase B: MeCN

Sample Diluent: DMSO

Injection Volume (µL): 0.5

**Times (mins)**	**%A**	**%B**	**Flow rate (mL/min)**
0.00	97	3	0.8
2.35	5	95	0.8
2.75	5	95	0.8
2.85	97	3	0.8
3.50	97	3	0.8

3.7. HPLC conditions method 7

254 mg of sample was dissolved in 12 mL DMSO:H_2O 2:10.

Instrument Name: Waters 'Scarlet' MDAP

Column: XBridge BEH C18 OBD Prep Column (5µm, 150 × 30 mm)

Mobile Phase A: H_2O + 0.1% v/v formic acid

Mobile Phase B: MeOH

Injection Volume: 1000 µL

Time (mins)	%A	%B	Flow Rate (mL/min)
0.0	90	10	40
4.0	90	10	40
4.5	90	10	40
16.0	50	50	40
16.5	40	60	40
16.6	0	100	40
19.0	0	100	40

Analytical Method Details

Instrument Name: Shimadzu 'Achiral Toolbox' UHPLC-MS

Column: BEH C18 (1.7µm, 30 mm × 2.1 mm)

Mobile phase A: H_2O + 0.1% v/v formic acid

Mobile phase B: MeOH

Temperature (°C): 30

Sample Diluent: Crude Analysis: DMSO, Post Prep Analysis: DMSO/H_2O (1:1)

Injection Volume (µL): 0.5

Time (mins)	%B	Flow Rate (mL/min)
0.00	1	0.8
2.35	95	0.8
2.75	95	0.8
2.85	95	0.8
3.50	1	0.8

3.8. HPLC conditions method 8

Instrument name: Agilent

Column: Agilent Prep Column C18, 100 Å (5µm, 21.2 mm × 50 mm)

Mobile phase A: H_2O + 0.1% v/v formic acid

Mobile phase B: MeCN + 0.1% v/v formic acid

Injection Volume: varied volume, typically 750 µL

Collection Mode: UV (DAD)

Time (min)	%A	%B	Flow rate (mL/min)
0.0	95	5	25
1.0	95	5	25
10.0	5	95	25
14.9	5	95	25
15.0	95	5	25

3.9. HPLC conditions method 9

170 mg of sample was dissolved in 2.55 mL DMSO (Method 1: Tb-DOTA-DBCO clicked to FA-PEG_5-N_3).

170 mg of sample was dissolved in 3.2 mL DMSO (Method 2: Tb-complexation of DOTA-DBCO clicked to FA-PEG_5-N_3).

Instrument Name: Waters 'Silver' MDAP

Column: XBridge BEH C18 OBD Prep Column (5µm, 150 x 30 mm)

Mobile Phase A: H_2O + 0.1% v/v formic acid

Mobile Phase B: MeOH

Injection Volume: varied volume, typically 500 µL

Collection Mode: UV (DAD) and MS(+/-)

The gradient was delivered by two pumps in order to use at-column dilution when injecting the sample. The gradient program used for the purification is summarised below:

Chromatographic Pump			Regeneration Pump			
Time (mins)	%B	Flow Rate (mL/min)	Time (mins)	%B	Flow Rate (mL/min)	Total Flow rate (mL/min)
0.0	25	4	0	15	36	40
4.0	25	4	4	15	36	40
4.5	25	20	4.5	25	20	40
16.0	60	20	16	60	20	40
16.5	80	20	16.5	80	20	40
16.6	100	20	16.6	100	20	40
100.0	100	20	100	100	20	40

Analytical Method Details

Instrument Name: Shimadzu 'Achiral Toolbox' UHPLC-MS

Column: BEH C18 (1.7µm, 30 mm x 2.1 mm)

Mobile phase A: H_2O + 0.1% v/v formic acid

Mobile phase B: MeOH

Temperature (°C): 30

Sample Diluent: DMSO

Injection Volume (µL): 0.5

Time (mins)	%A	%B	Flow rate (mL/min)
0	99	1	0.8
2.35	5	95	0.8
2.75	5	95	0.8
2.85	5	95	0.8
3.5	99	1	0.8

4. HPLC traces of complexes:

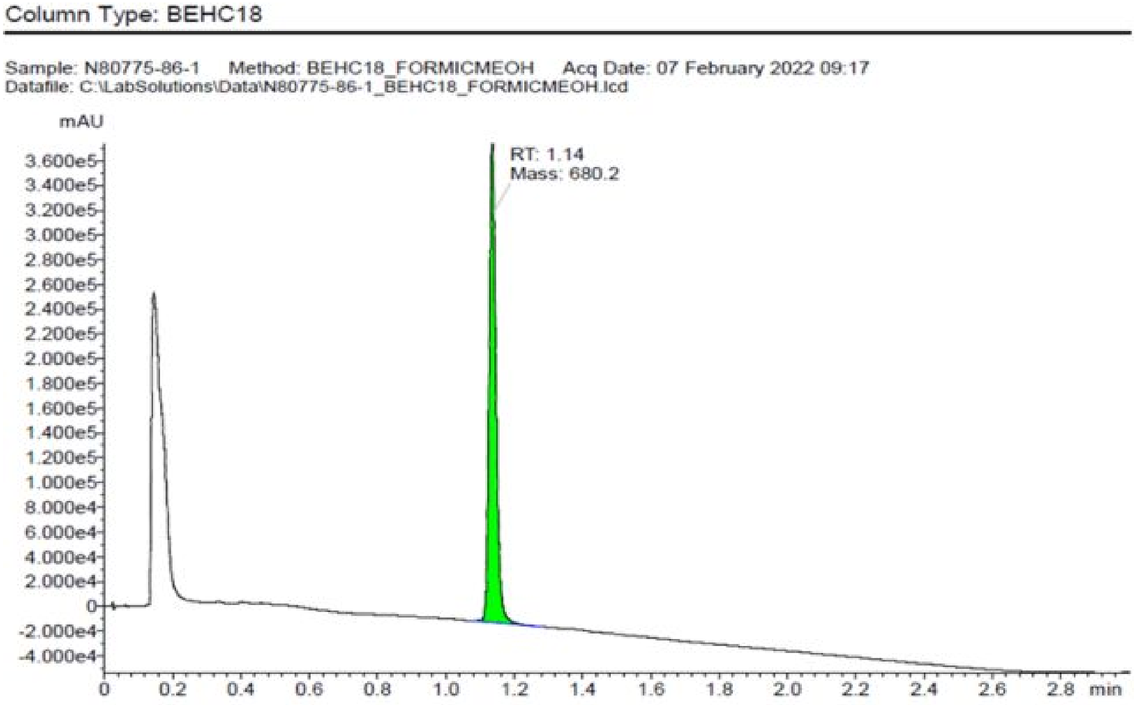

RT (mins)	Peak Area	Peak Area (%)	Sample/Target
1.14	551006	100	N80775-86-1 (680.2)

*Figure S3. Analytical HPLC trace of Tb**L**[1α], t_R = 1.15 min, 5.4 mg, UV purity >98%. HPLC conditions method 2.*

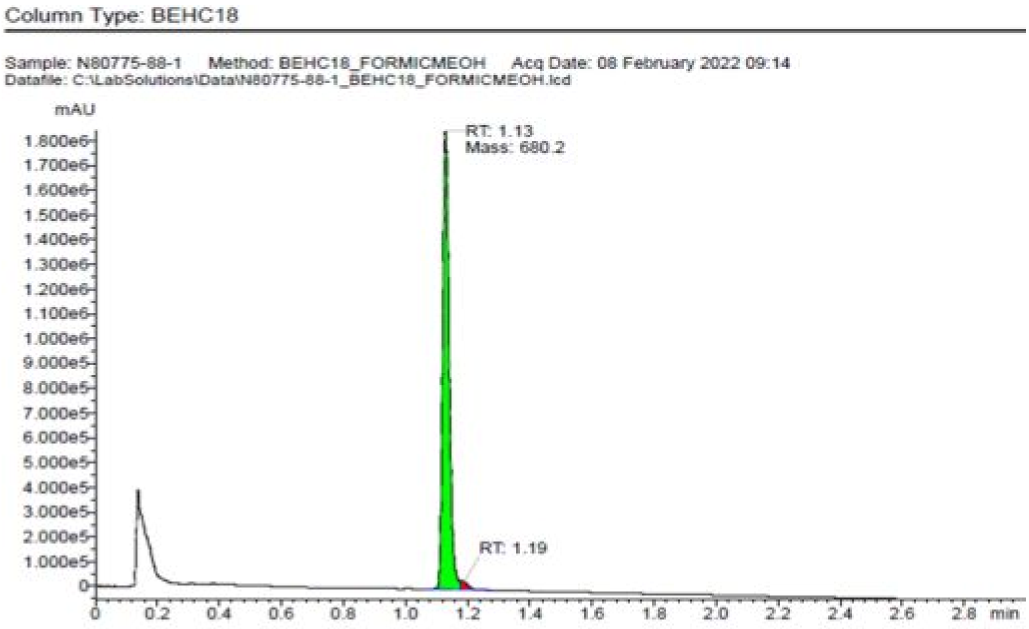

RT (mins)	Peak Area	Peak Area (%)	Sample/Target
1.13	2762014	97.824	N80775-88-1 (680.2)
1.19	61453	2.176	-

*Figure S4. Analytical HPLC trace of Tb**L**[1γ], t_R = 1.13 min, 33.3 mg, UV purity >97%. HPLC conditions method 3.*

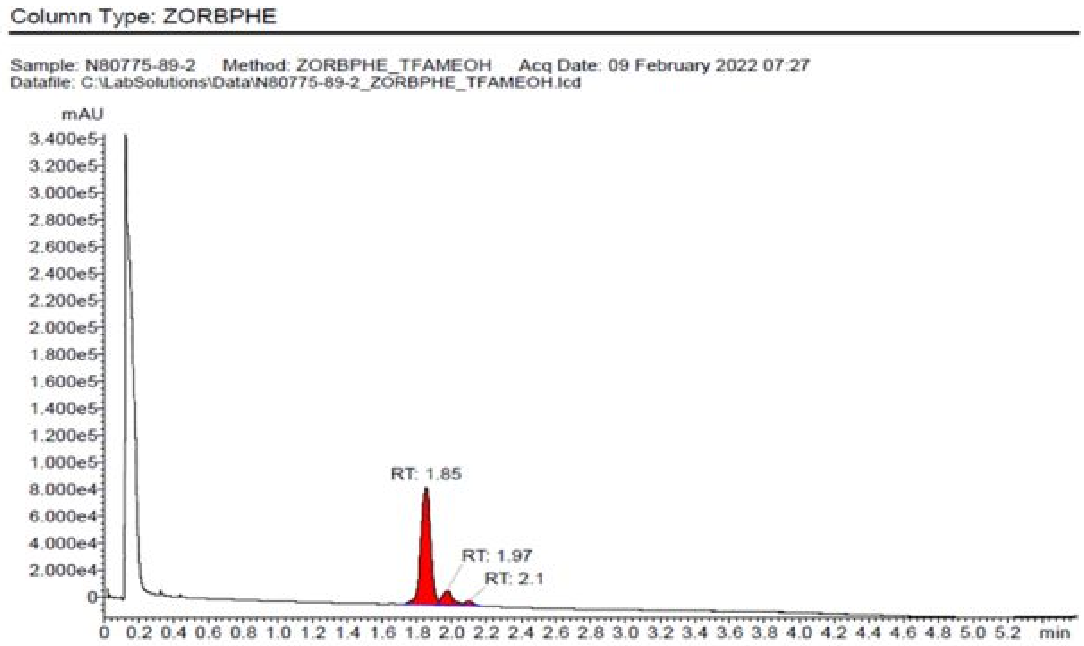

RT (mins)	Peak Area	Peak Area (%)	Sample/Target
1.85	345193	85.394	-
1.97	47111	11.654	-
2.1	11931	2.951	-

*Figure S5. Analytical HPLC trace of Tb**L**^{2}, t_R = 1.18 min, 18.5 mg, UV purity >85%. HPLC conditions 5.*

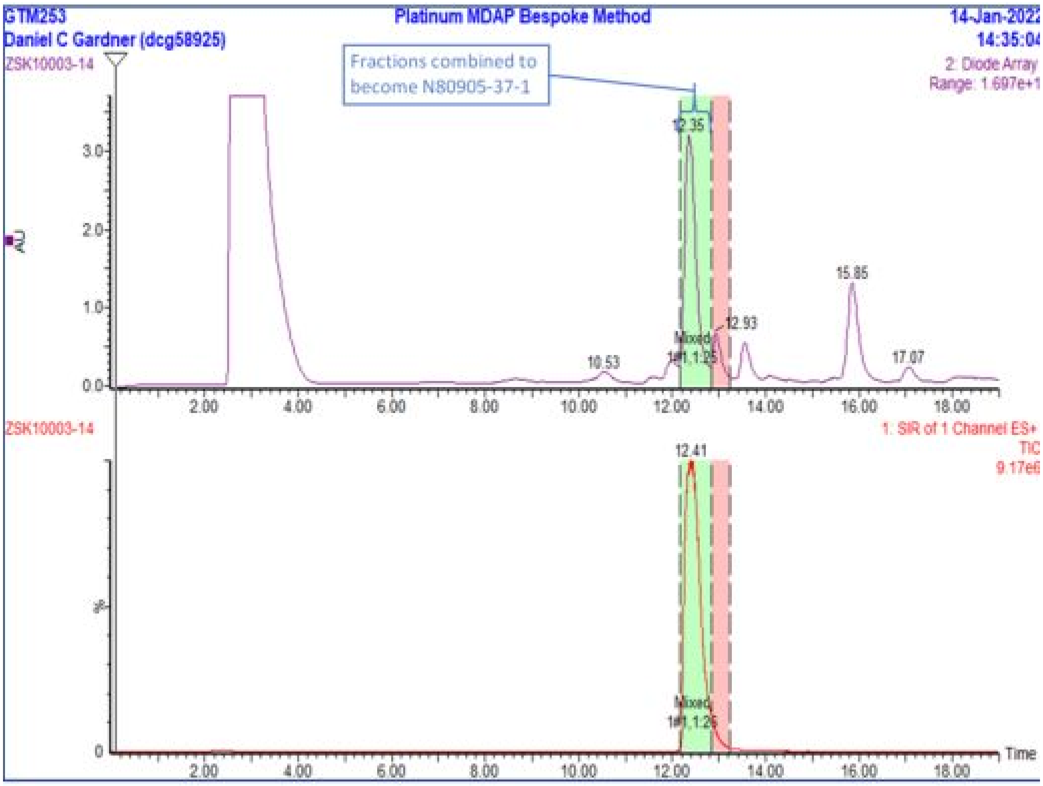

*Figure S6. Chromatograms of preparative HPLC of Tb**L**^{3} containing a mixture of isomers top trace: diode array; bottom trace: total ionisation chromatogram.*

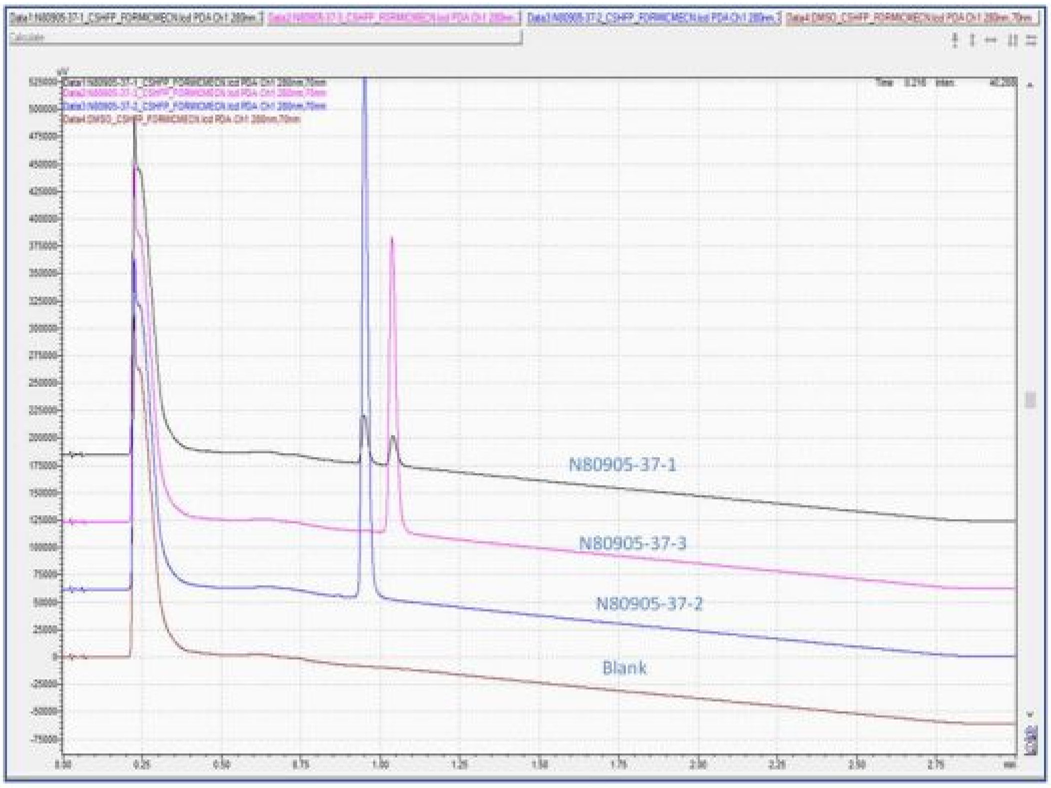

*Figure S7.Overlay of HPLC chromatograms. Tb**L**^{3} mixture of isomers (black, top); Tb**L**$^{3\ minor}$(pink, 2nd from top); Tb**L**$^{3\ major}$ (blue, 2nd from bottom), blank (maroon, bottom). Instrument: Toolbox, Method: CSHFP_FormicMECN*

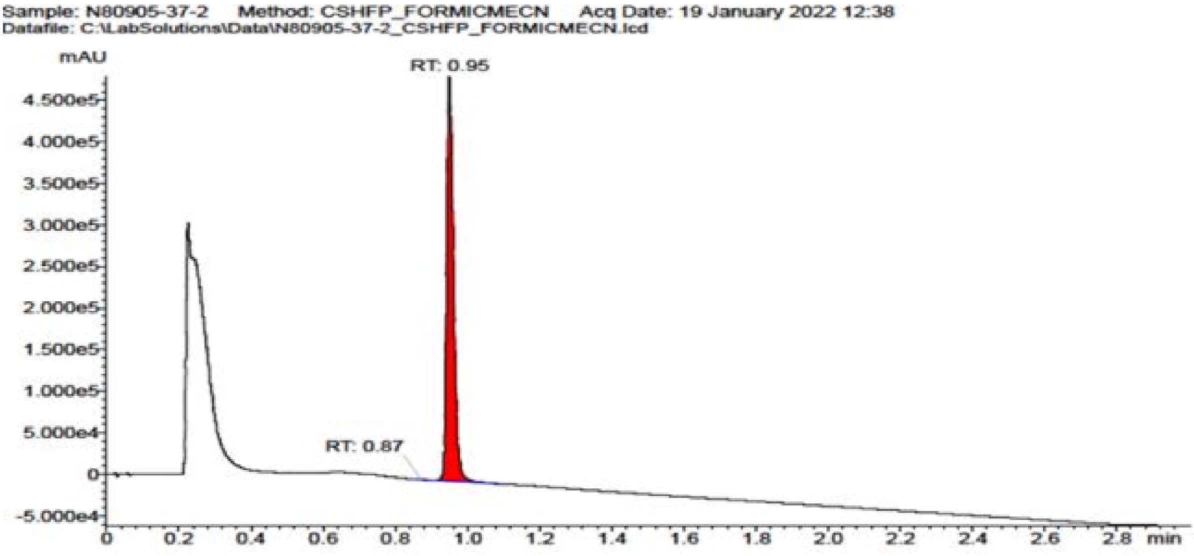

RT (mins)	Peak Area	Peak Area (%)	Sample/Target
0.87	1017	0.154	-
0.95	657738	99.846	-

Figure S8. Analytical HPLC of TbL^{3major}, t_R = 0.95 min, 26.6 mg, UV purity >99%. HPLC conditions method 6.

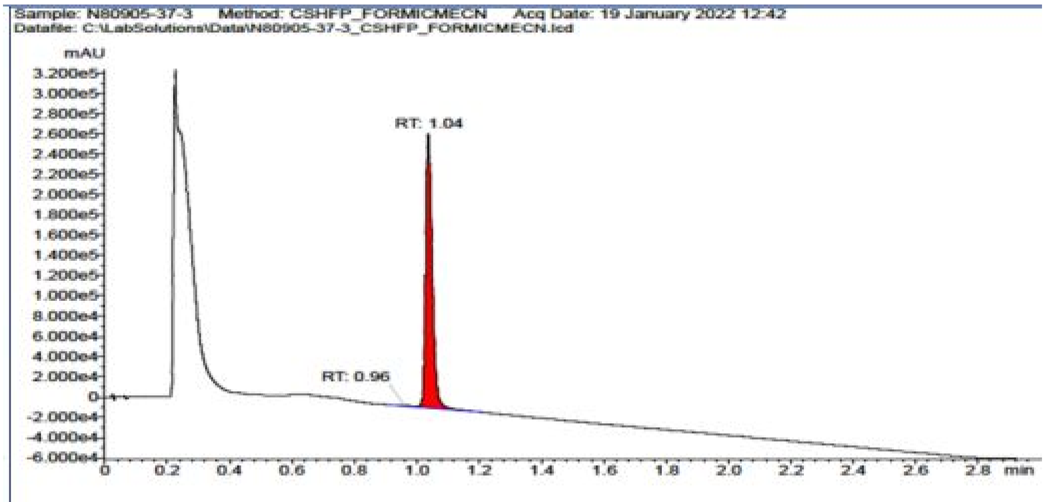

RT (mins)	Peak Area	Peak Area (%)	Sample/Target
0.96	2269	0.55	-
1.04	410449	99.45	-

Figure S9. Analytical HPLC trace of Tb$\mathbf{L}^{3\ minor}$, t_R =1.04 min, 17.6 mg, UV purity >99%. HPLC conditions method 6.

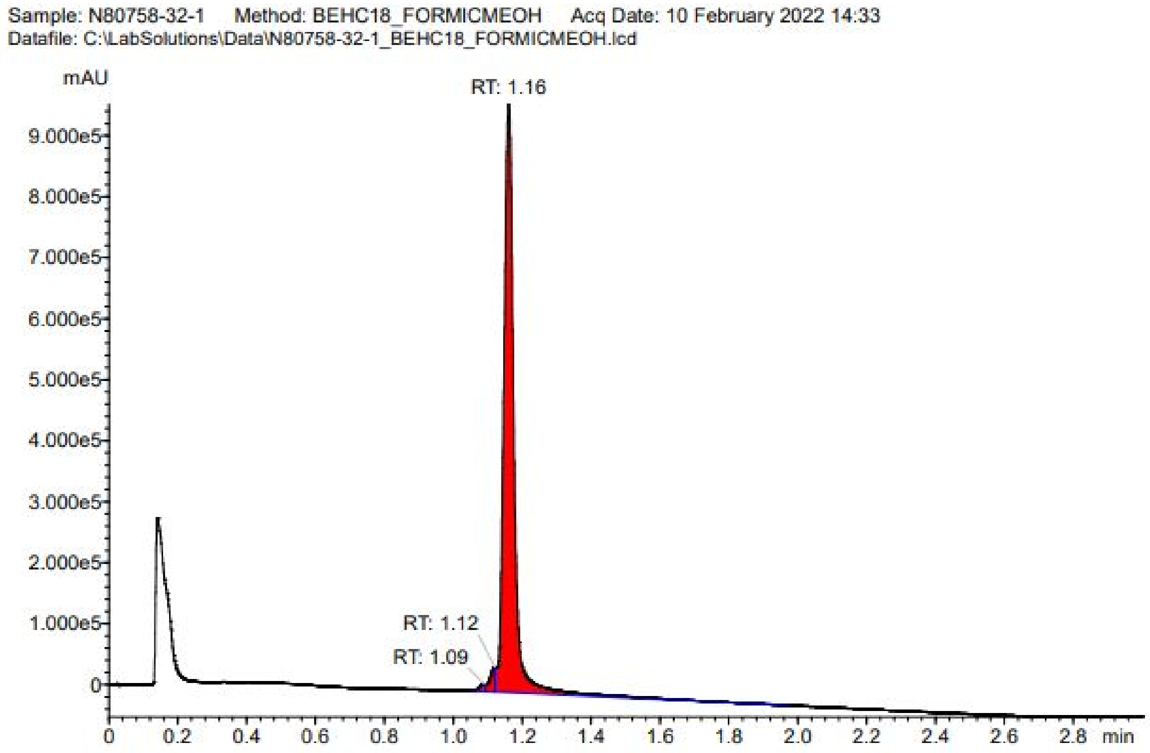

RT (mins)	Peak Area	Peak Area (%)	Sample/Target
1.09	15109	0.74	-
1.12	40000	1.959	-
1.16	1986377	97.301	-

Figure S10. Analytical HPLC trace of Tb$\mathbf{L}^4$, t_R =1.16 min, 41.4 mg, UV purity >97%. HPLC conditions method 7.

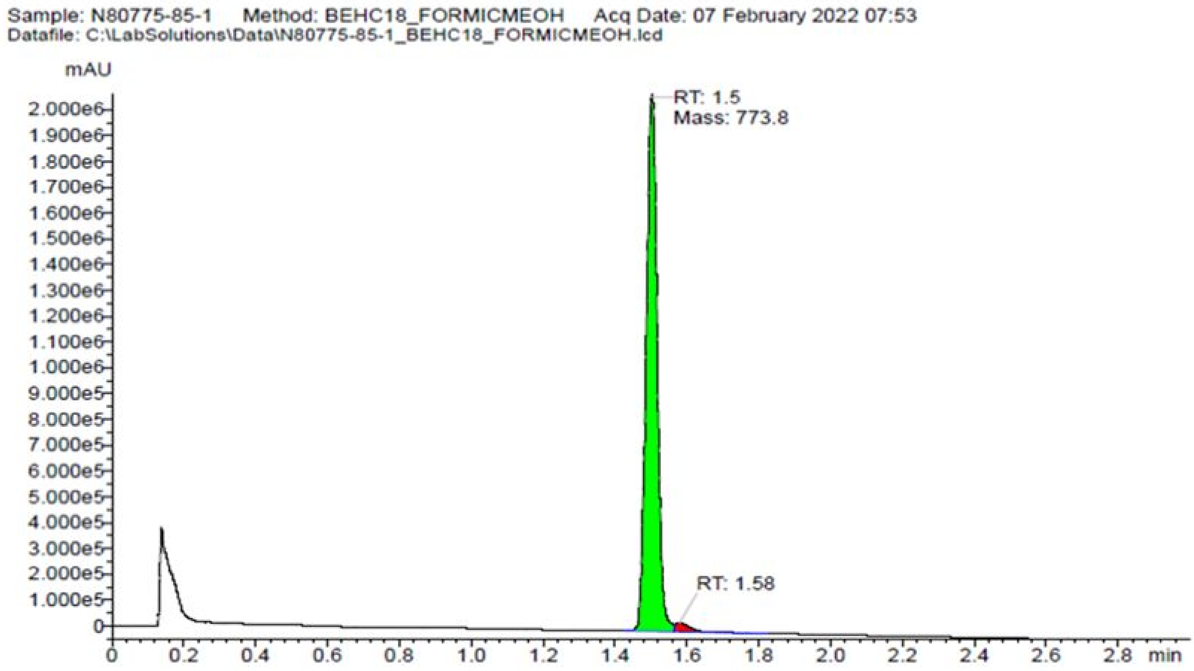

RT (mins)	Peak Area	Peak Area (%)	Sample/Target
1.5	4429725	97.666	N80775-85-1 (773.8)
1.58	105873	2.334	-

*Figure S11. Analytical HPLC trace of Tb**L**^{5}, t_R =1.5 min, 90.9 mg, UV purity >97%. HPLC conditions method 9.*

5. ^{1}H NMR spectra

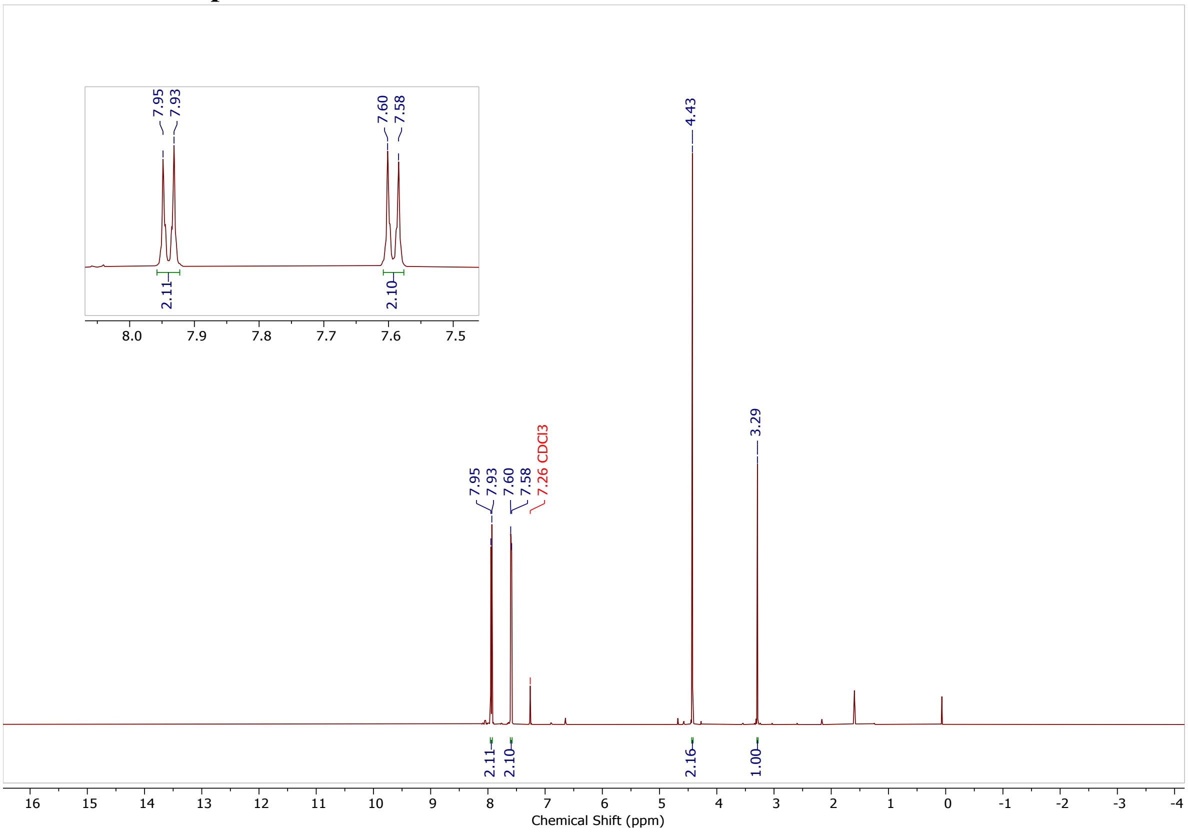

*Figure S12. ^{1}H NMR (500 MHz, $CDCl_3$) spectrum of **1**, 2-bromo-1-(4-ethynylphenyl)ethan-1-one.*

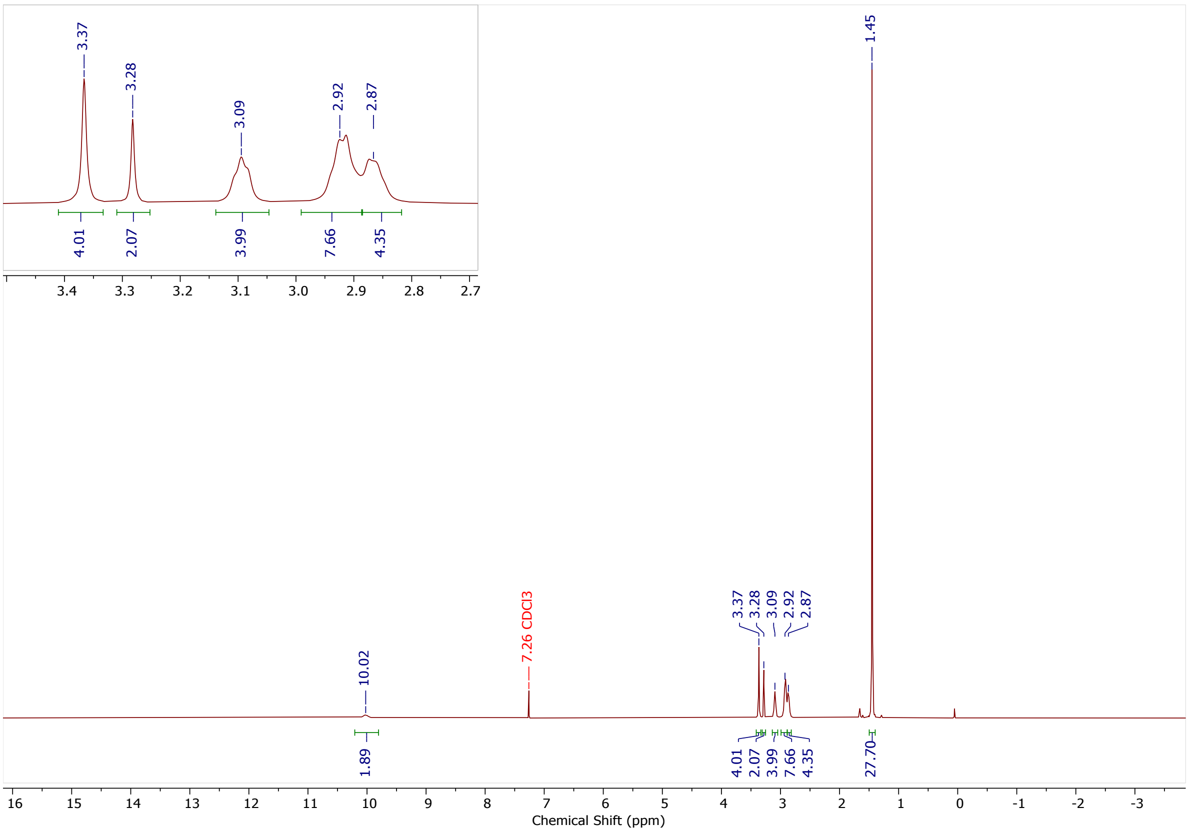

*Figure S 13. ^{1}H NMR (400 MHz, $CDCl_3$) spectrum of **2**, 1,4,7-Tris(tert-butoxycarbonylmethyl)-1,4,7,10-tetraazacyclododecane hydrobromide salt.*

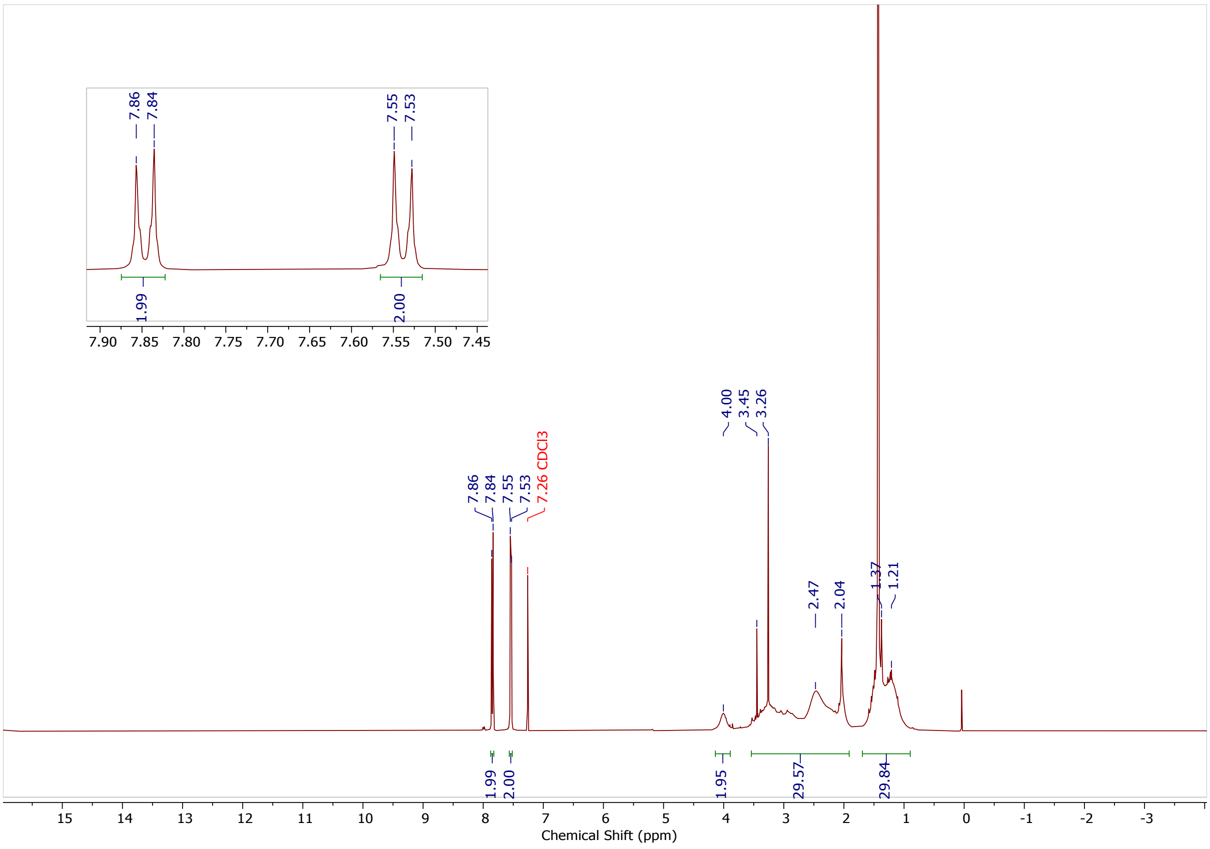

Figure S14. ^{1}H *NMR (400 MHz,* $CDCl_3$*) spectrum of* ***3****, 1-(4'-ethynyl-2-acetophenone)-4,7,10-tris(tert-butoxycarboxymethyl)-1,4,7,10-tetraazacyclododecane.*

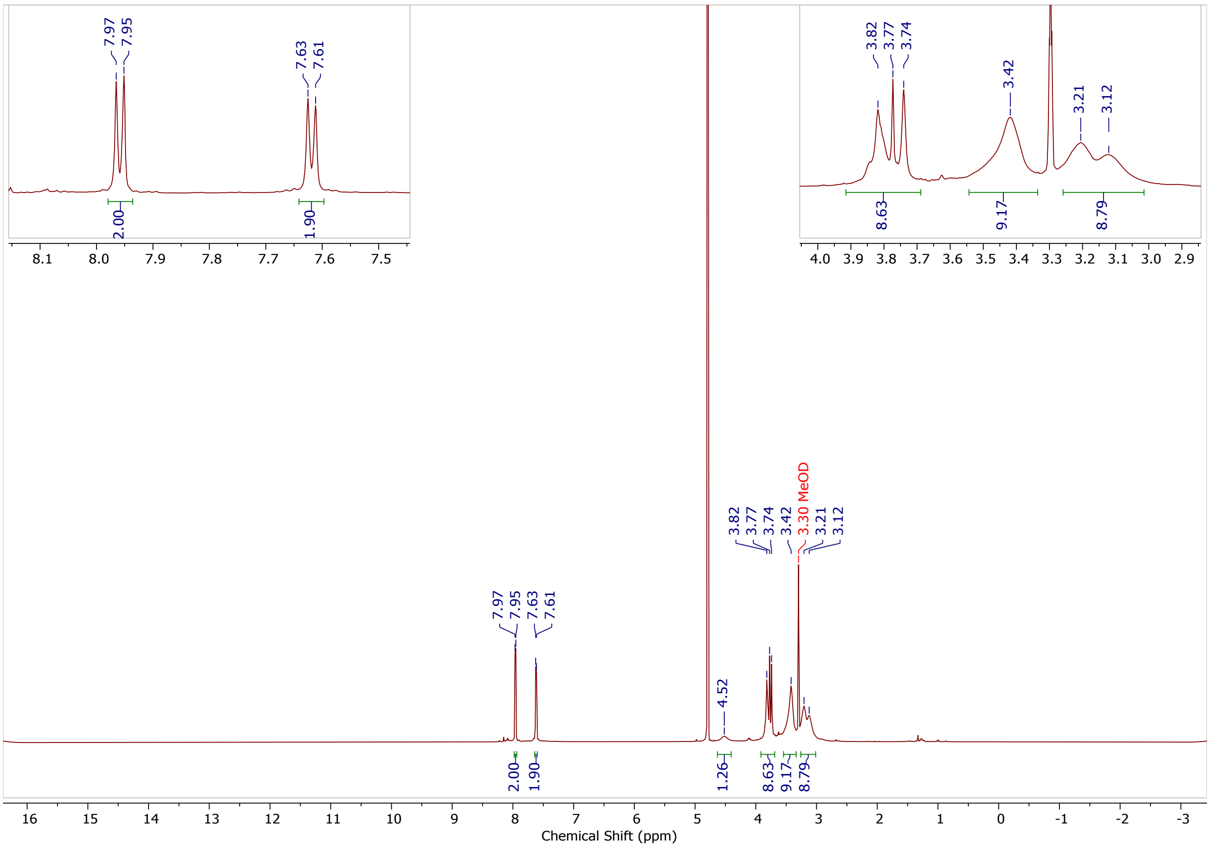

Figure S15. ^{1}H *NMR (600 MHz,* D_2O/CD_3OD *(1:1)) spectrum of* ***4****, 1-(4'-ethynyl-2-acetophenone)-4,7,10-tris(carboxymethyl)-1,4,7,10-tetraazacyclododecane*

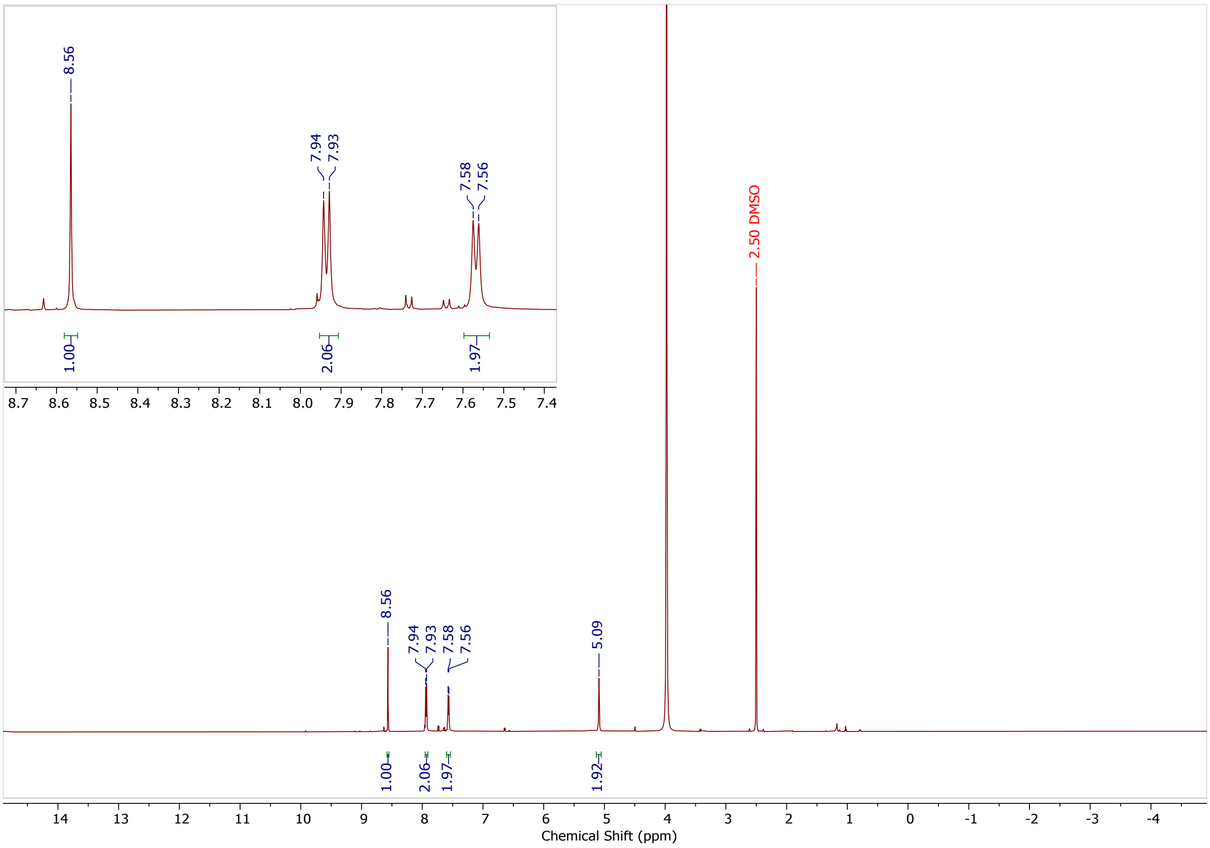

Figure S16. ^{1}H NMR (600 MHz, DMSO-d_6/D_2O (8:2)) spectrum of N^{10}-(TFA)-pteroic acid.

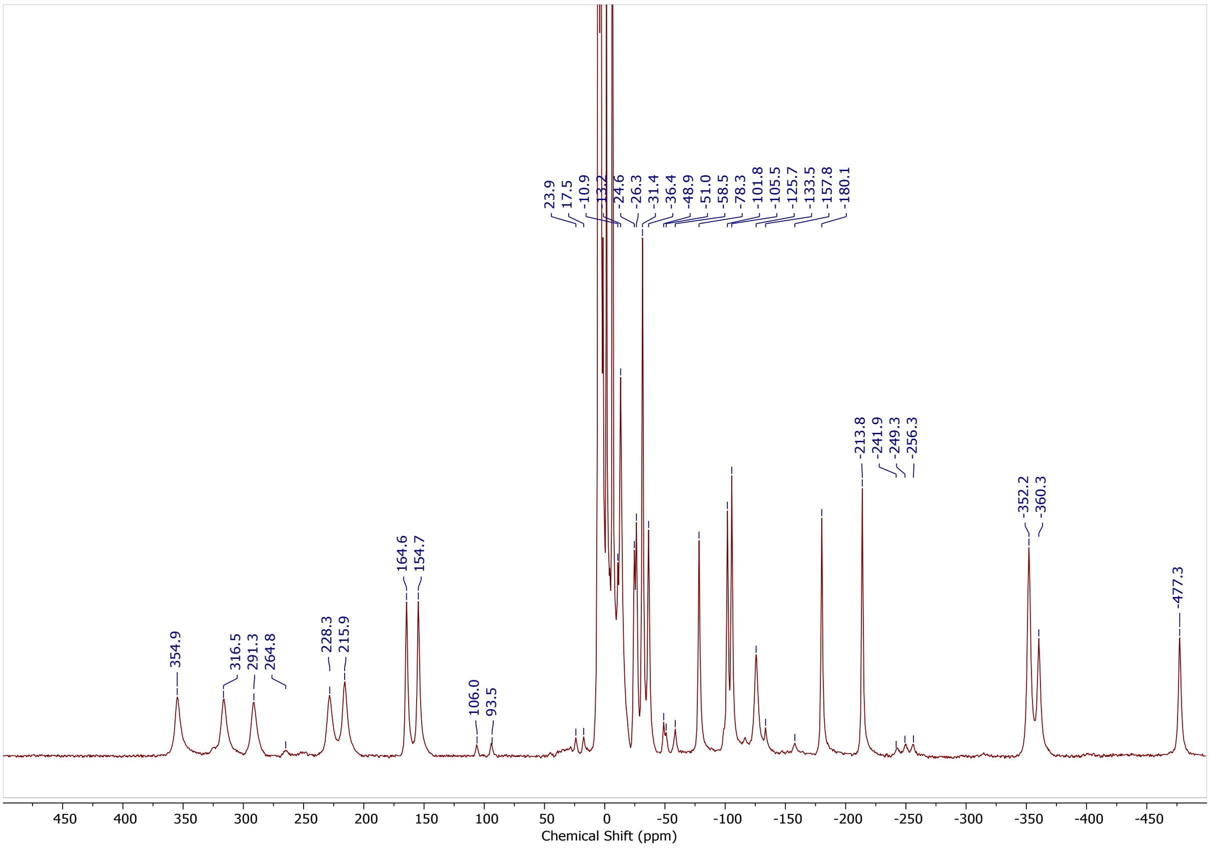

*Figure S17. ^{1}H NMR (400 MHz, MeOD) spectrum of **Tb4**, Tb-1-(4'-ethynyl-2-acetophenone)-4,7,10-tris(carboxymethyl)-1,4,7,10-tetraazacyclododecane.*

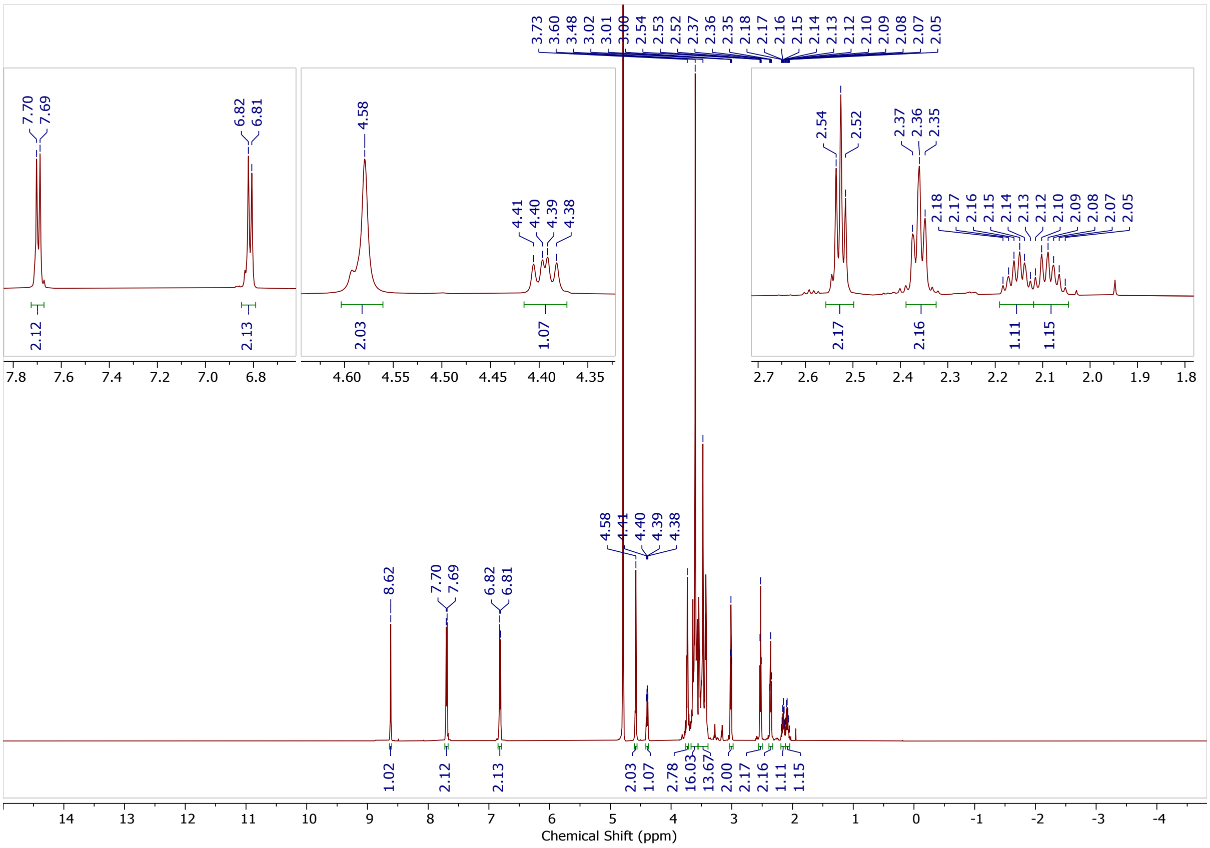

Figure S18. ^{1}H NMR (600 MHz, D_2O, pH 9) spectrum of **S2α**, α-isomer-FA-PEG$_6$-EDA.

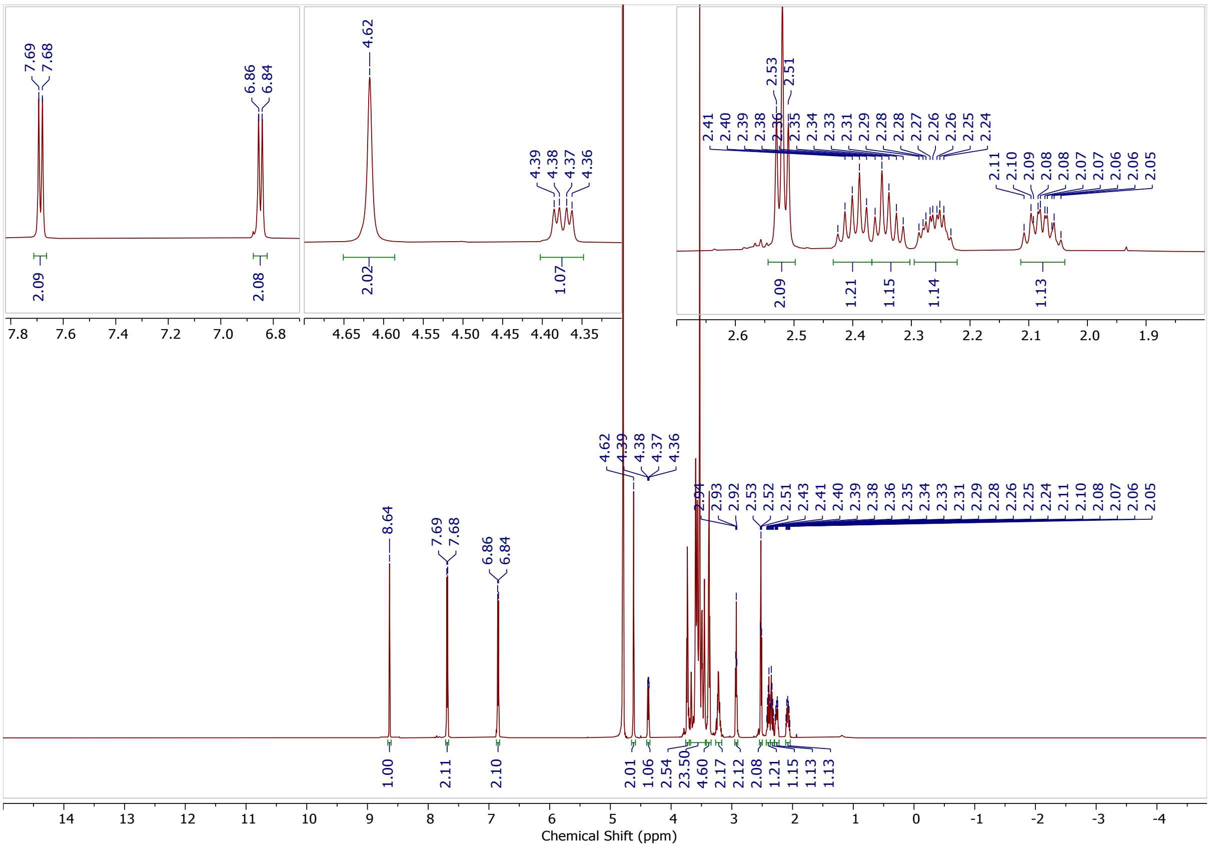

Figure S19. ^{1}H NMR (600 MHz, D_2O, pH 9) spectrum of **S2γ**, γ-isomer-FA-PEG$_6$-EDA.

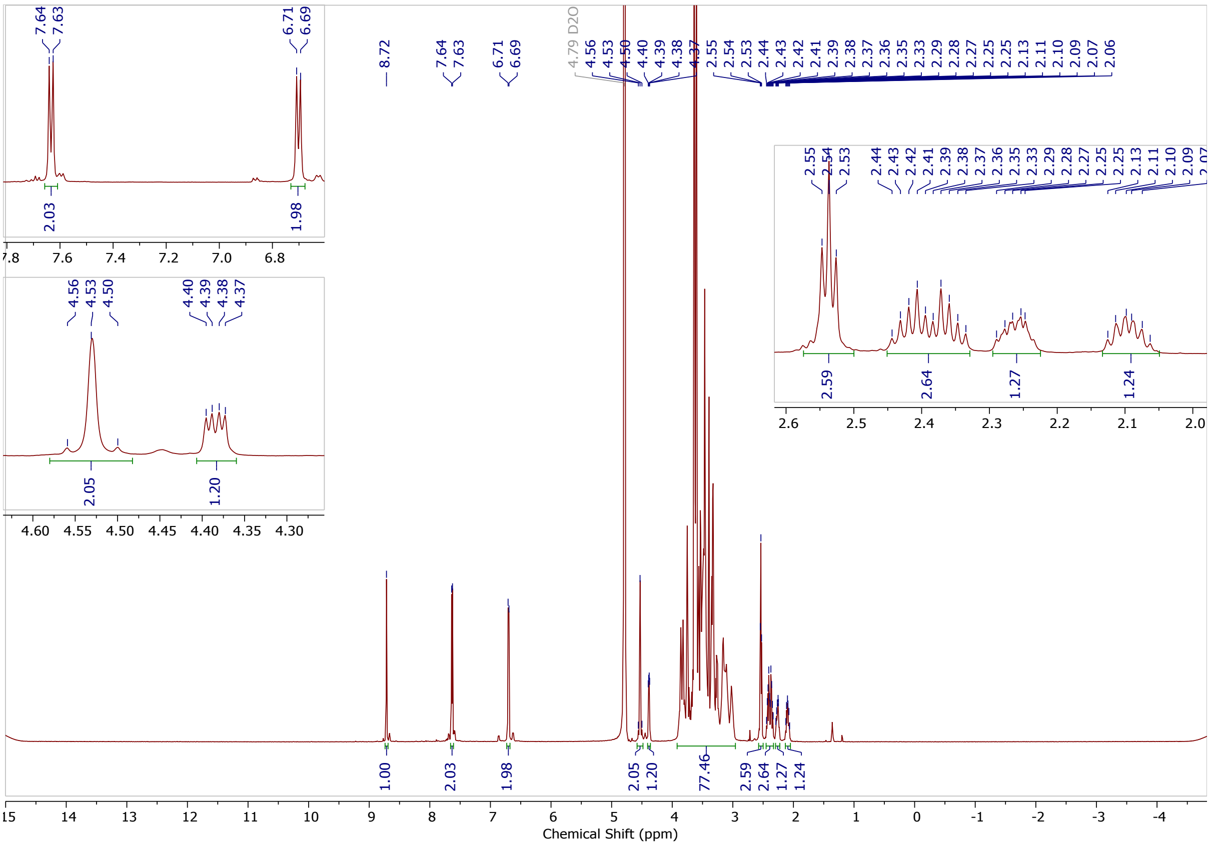

Figure S20. [1]*H NMR (600 MHz,* D_2O*, pH 9) spectrum of* $\boldsymbol{L^{1\gamma}}$*, γ-isomer-FA-PEG*$_6$*-EDA-DOTA.*

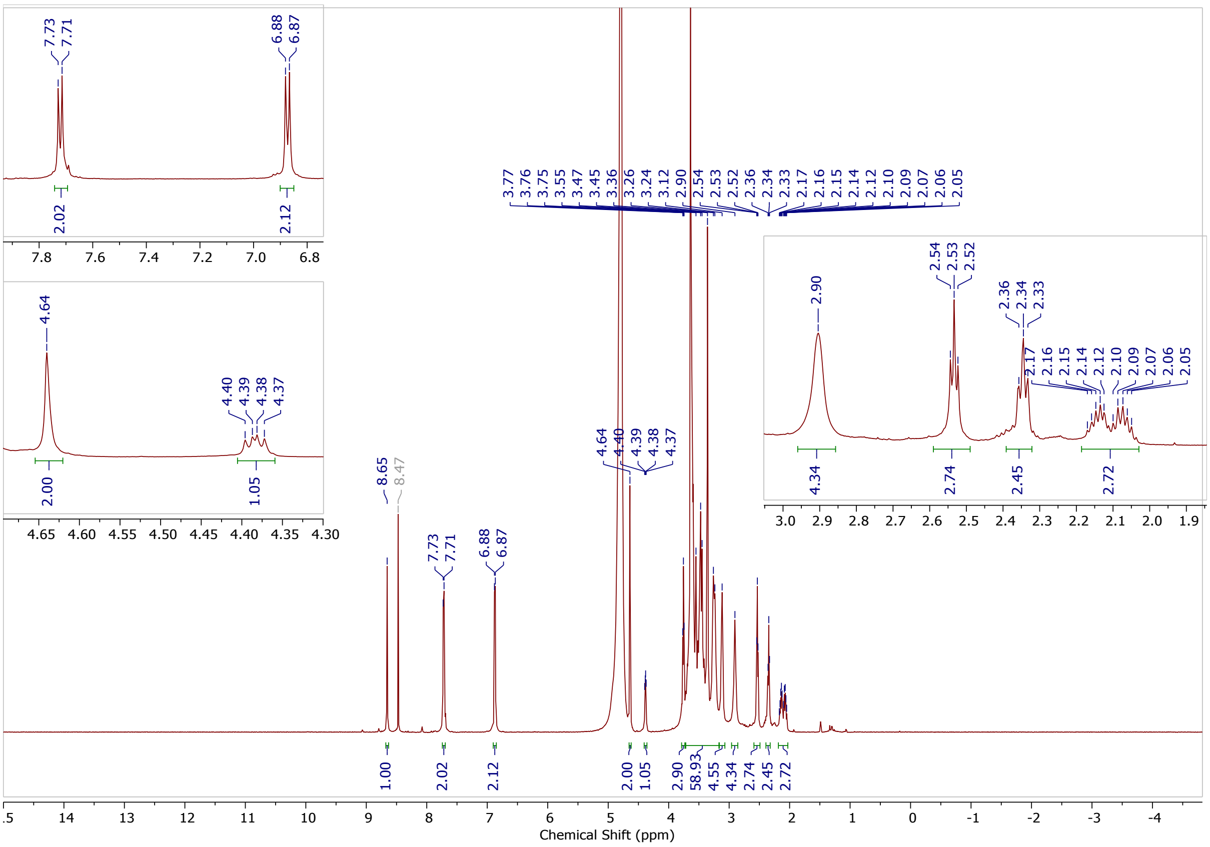

Figure S21. [1]*H NMR (600 MHz,* D_2O*, pH 9) spectrum of* $\boldsymbol{L^{1\alpha}}$*, α-isomer-FA-PEG*$_6$*-EDA-DOTA.*

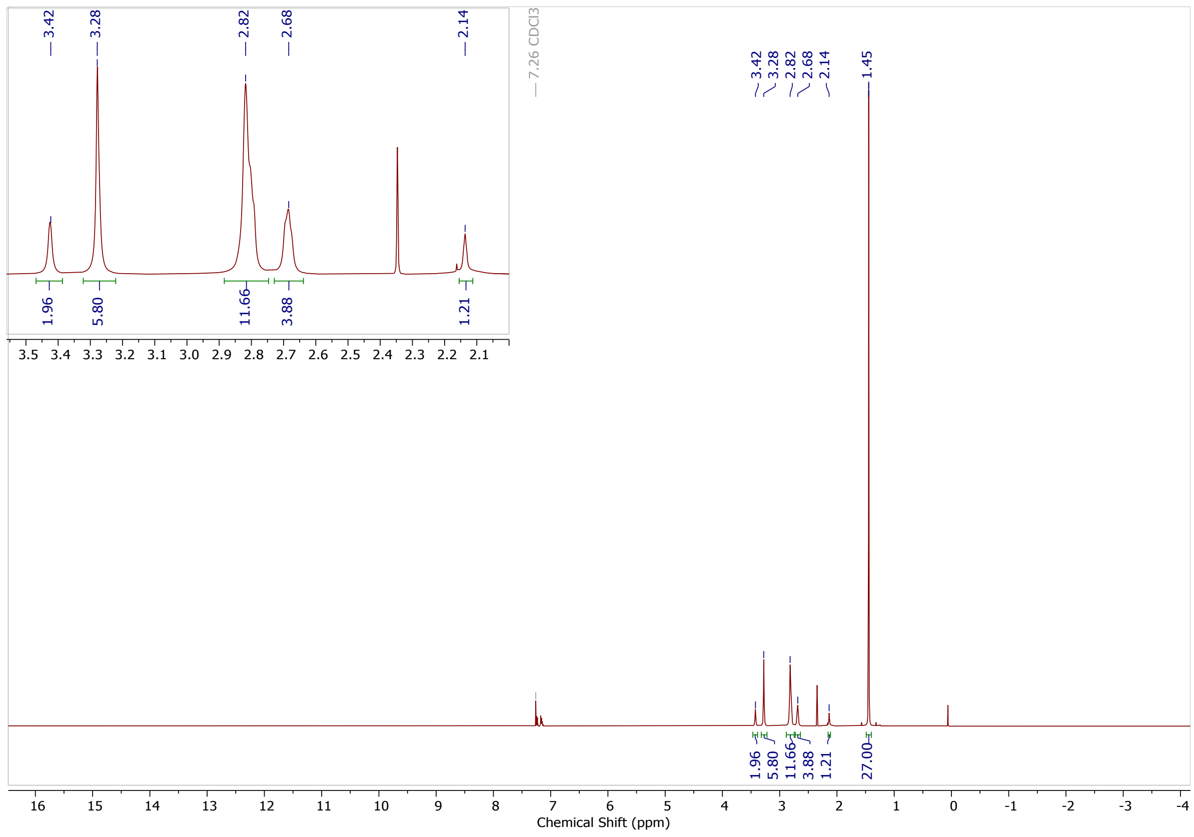

*Figure S22. ¹H NMR (500 MHz, D_2O) spectrum of **S3**, propargyl-tBu_3DO3A. Trace toluene at 7.25, 7.15 and 2.34 ppm.*

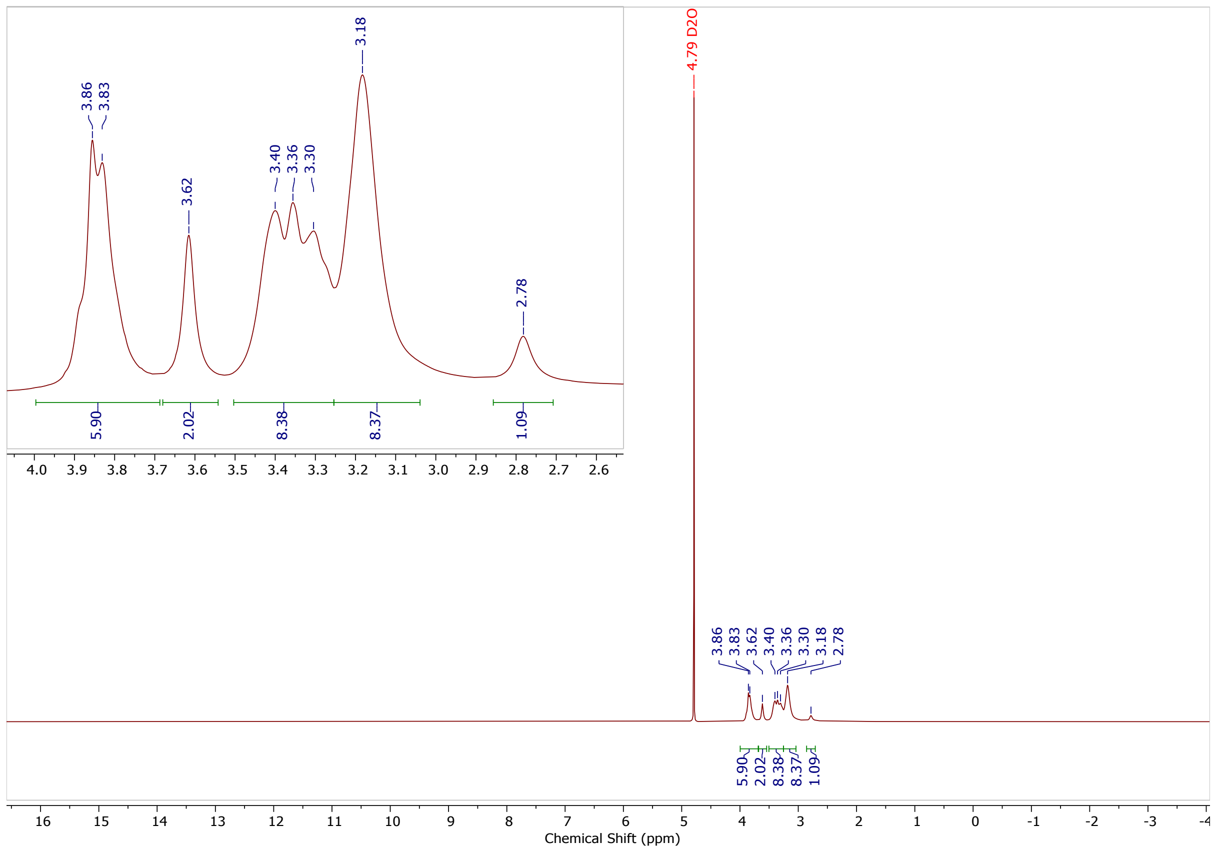

*Figure S23. ¹H NMR (500 MHz, D_2O) spectrum of **7**, propargyl-DO3A.*

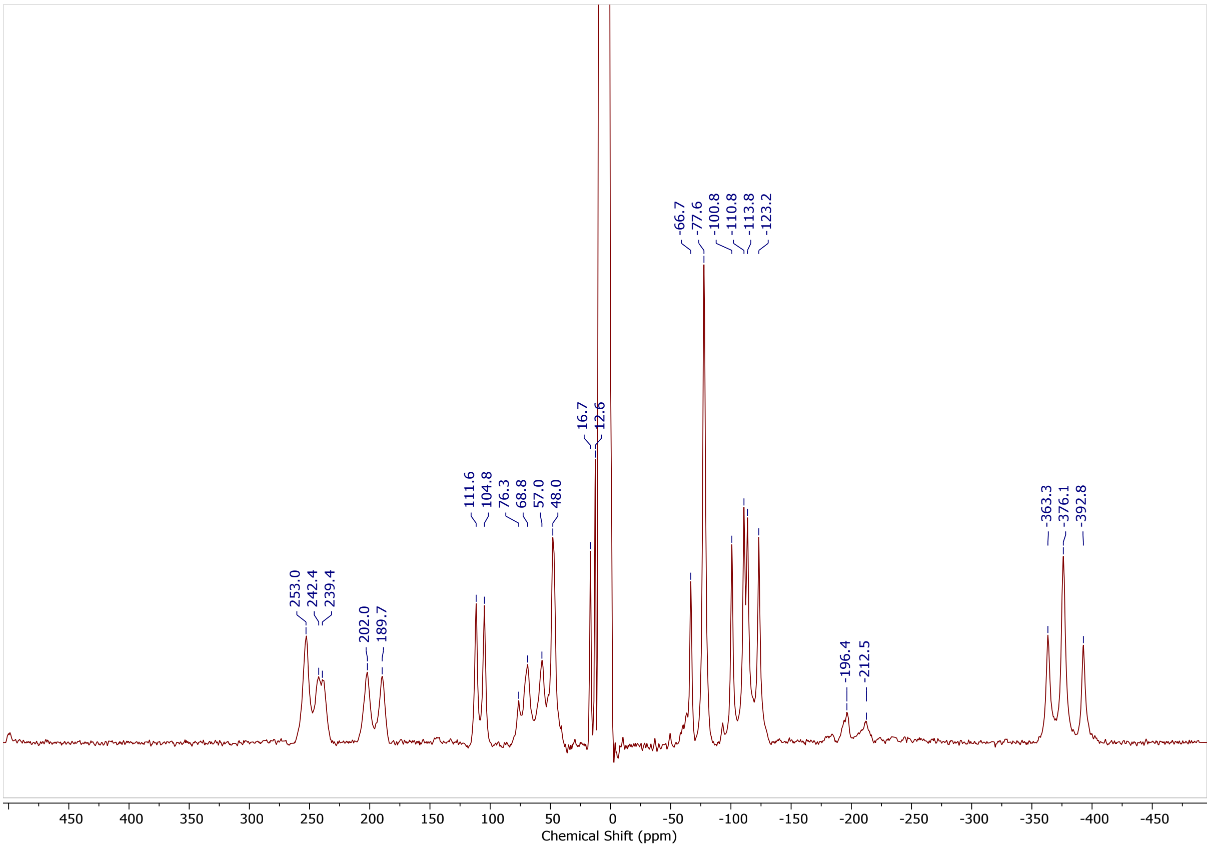

Figure S24. ^{1}H NMR (500 MHz, D_2O) spectrum of ***$TbL^{1\gamma}$****, Tb.DOTA-EDA-PEG$_6$-FA (γ-isomer).*

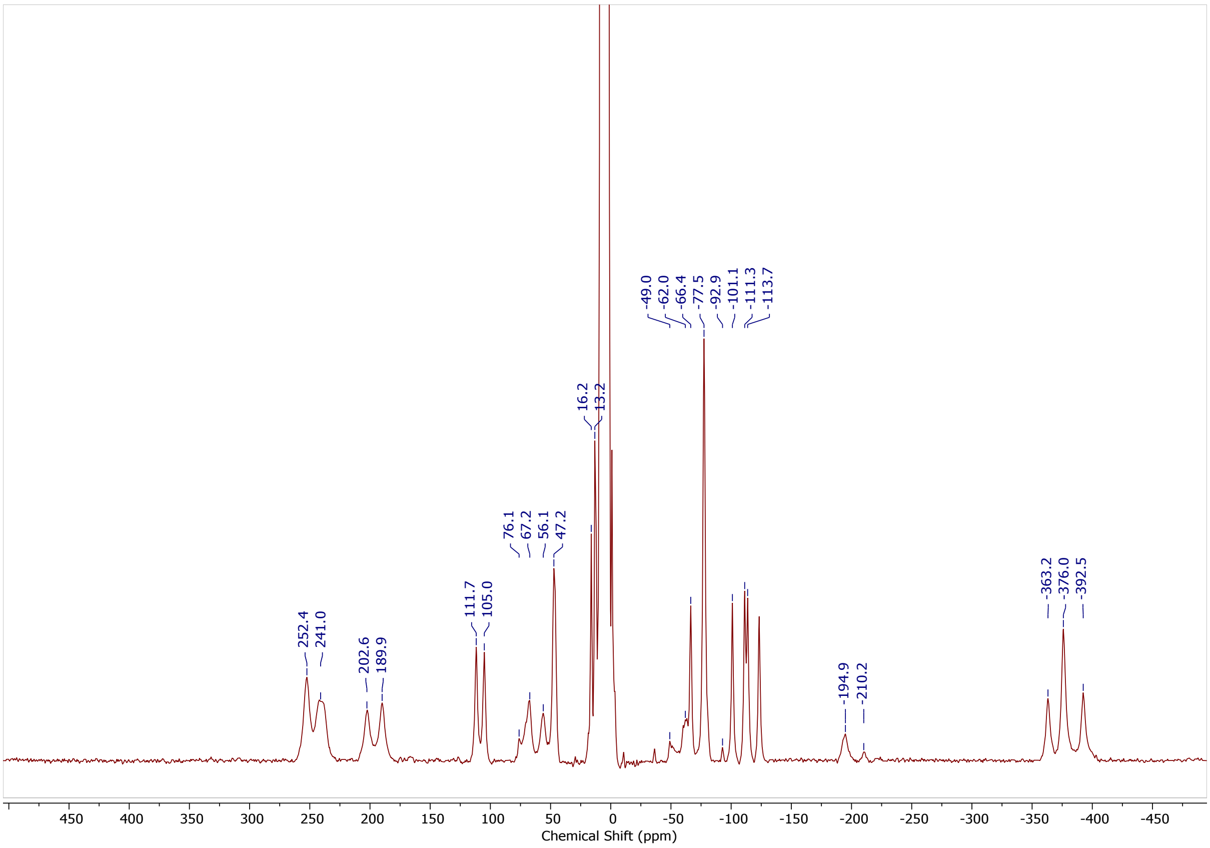

Figure S25. ^{1}H NMR (500 MHz, D_2O) spectrum of ***$TbL^{1\alpha}$****, Tb.DOTA-EDA-PEG$_6$-FA (α-isomer).*

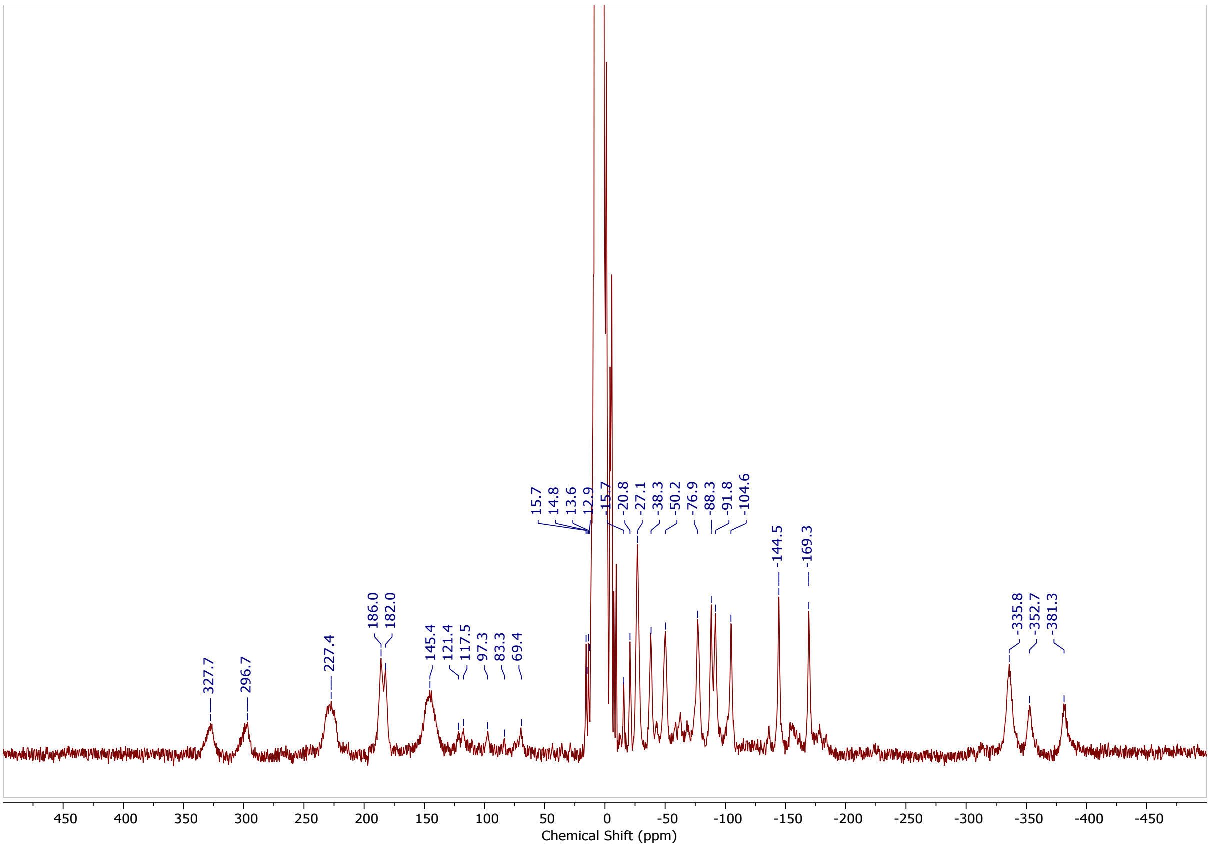

*Figure S26. ^{1}H NMR (400 MHz, D_2O/drop DMSO-d_6) spectrum of **TbL²**, Tb.pDO3A clicked to FA-PEG-N_3.*

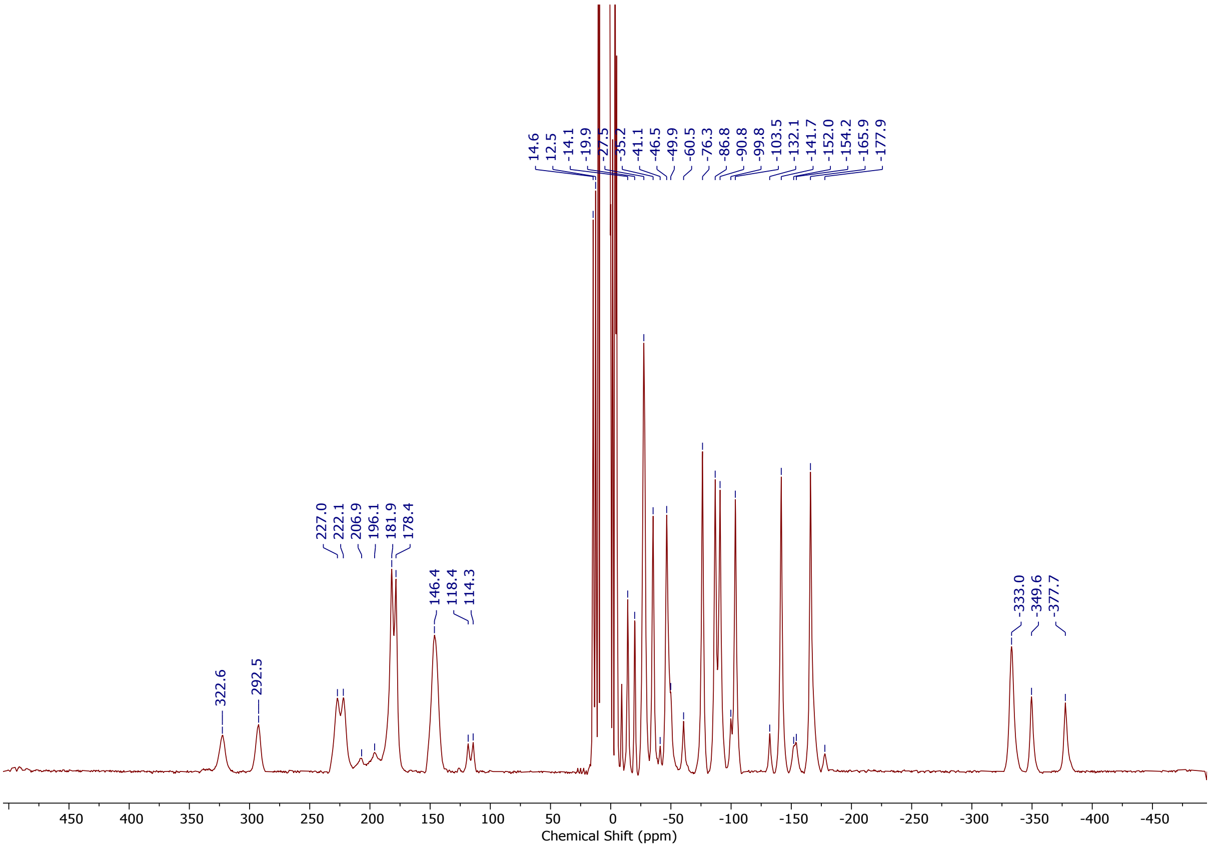

*Figure S27. ^{1}H NMR (500 MHz, D_2O) spectrum of $\textbf{TbL}^{\textbf{3major}}$**,** Tb.pDO3A clicked to FA-PEG$_5$-N_3 (major isomer).*

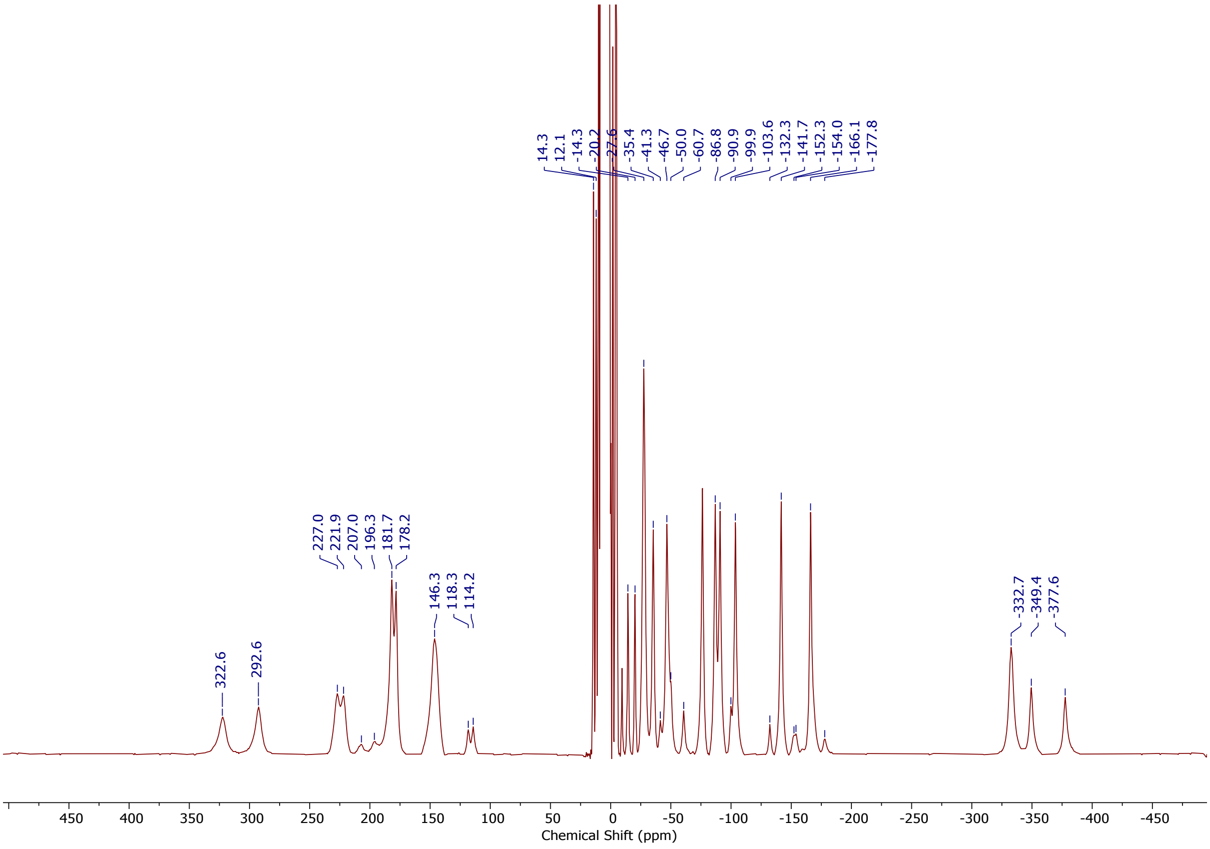

*Figure S28. ^{1}H NMR (500 MHz, D_2O) spectrum of **TbL^{3minor},** Tb.pDO3A clicked to FA-PEG$_5$-N$_3$ (minor isomer).*

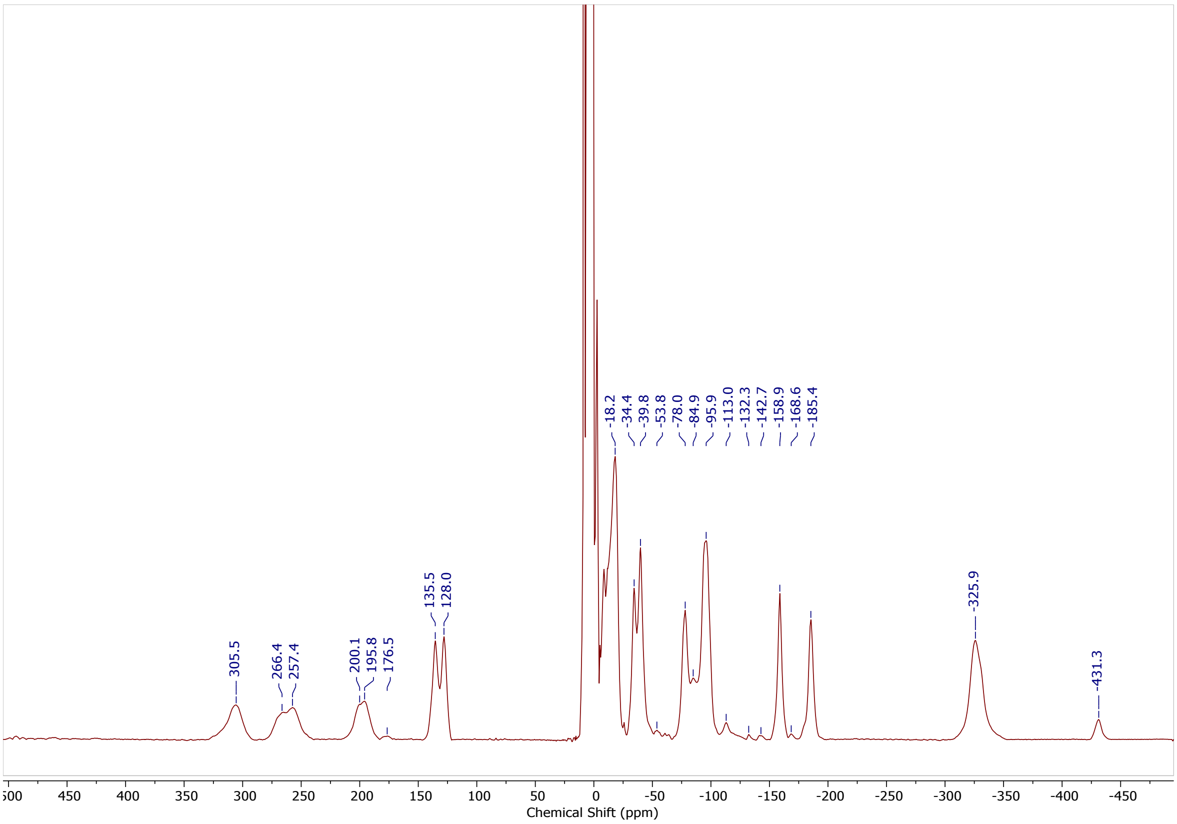

*Figure S29. ^{1}H NMR (500 MHz, D_2O) spectrum of **TbL^4** Tb.ethynylacetophenoneDO3A clicked to FA-PEG$_5$-N$_3$ (mixture of regioisomers).*

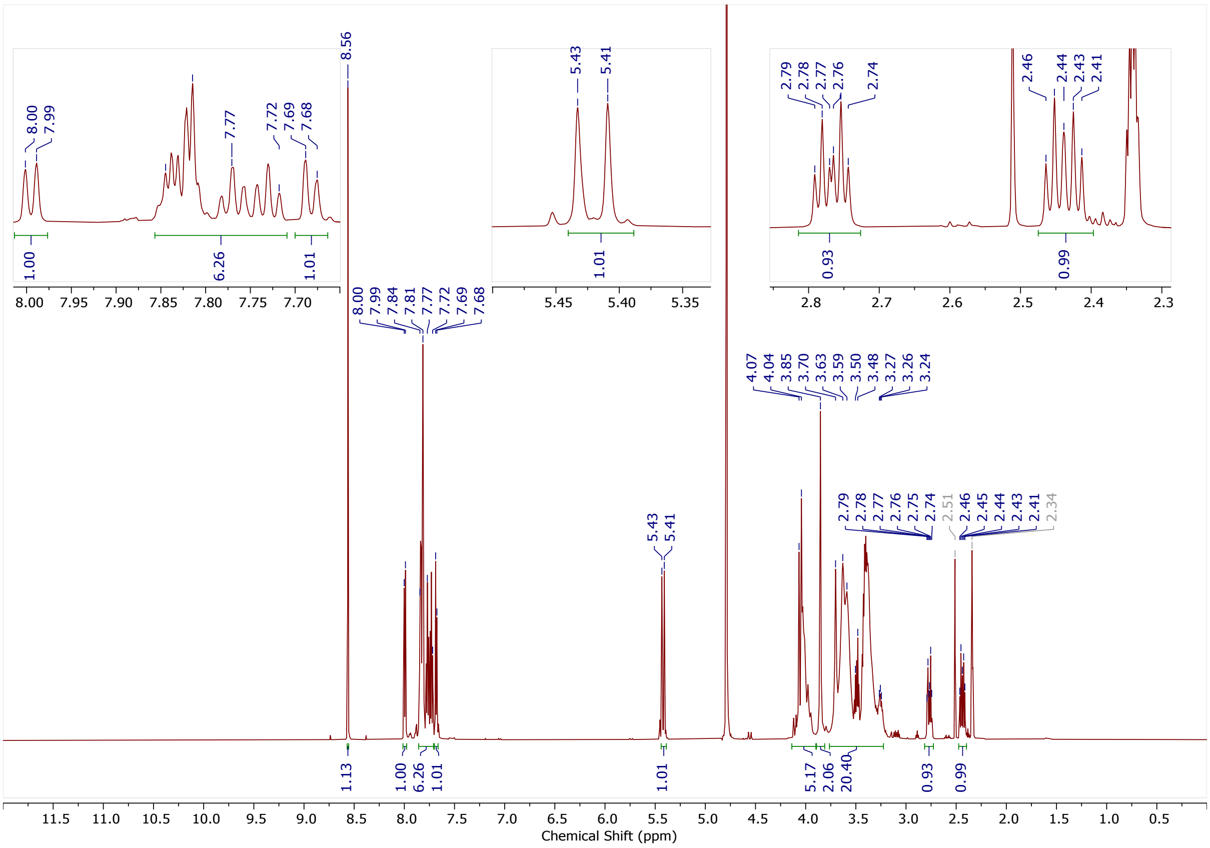

Figure S30. ^{1}*H NMR (600 MHz,* D_2O*) spectrum of* ***S6****, DOTA-DBCO.*

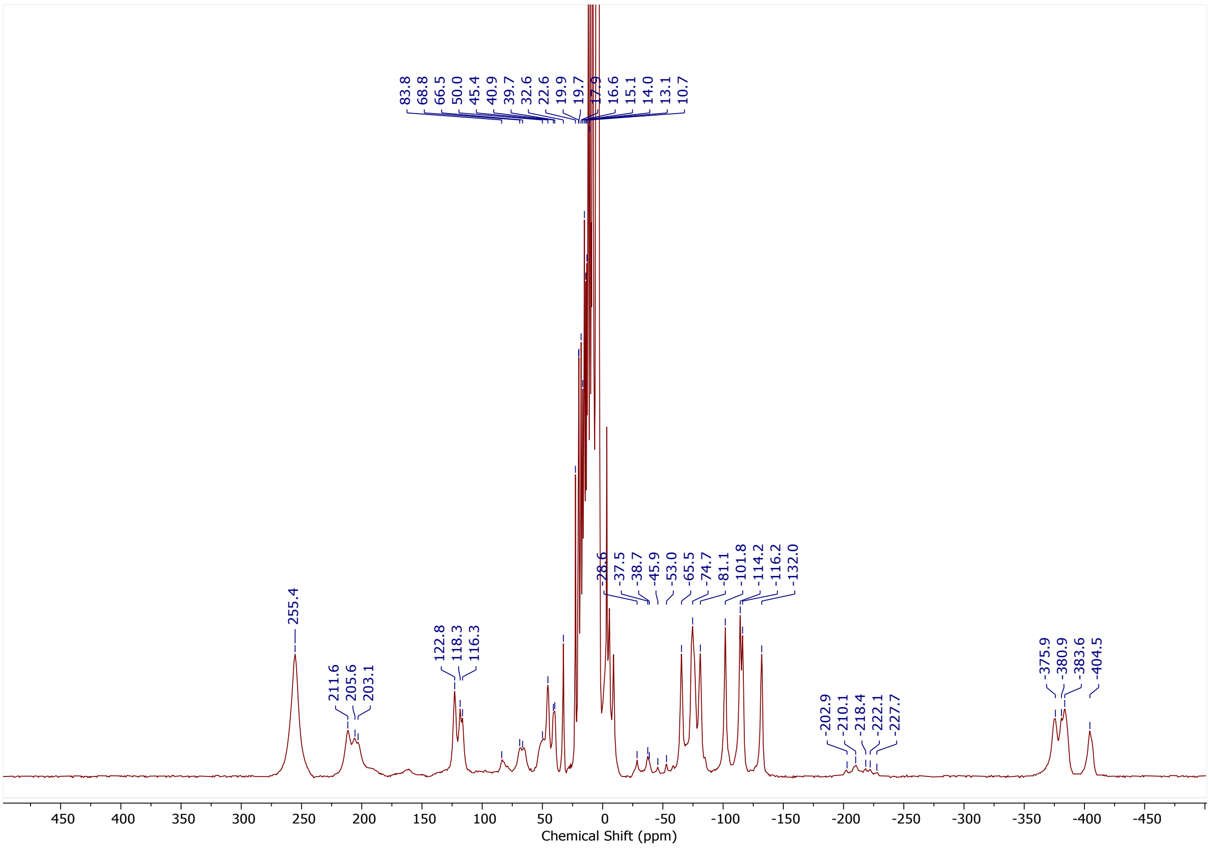

Figure S31. ^{1}*H NMR (400 MHz, 7* D_2O*: 3*CD_3CN*) spectrum of Tb****S6****, Tb-DOTA-DBCO*

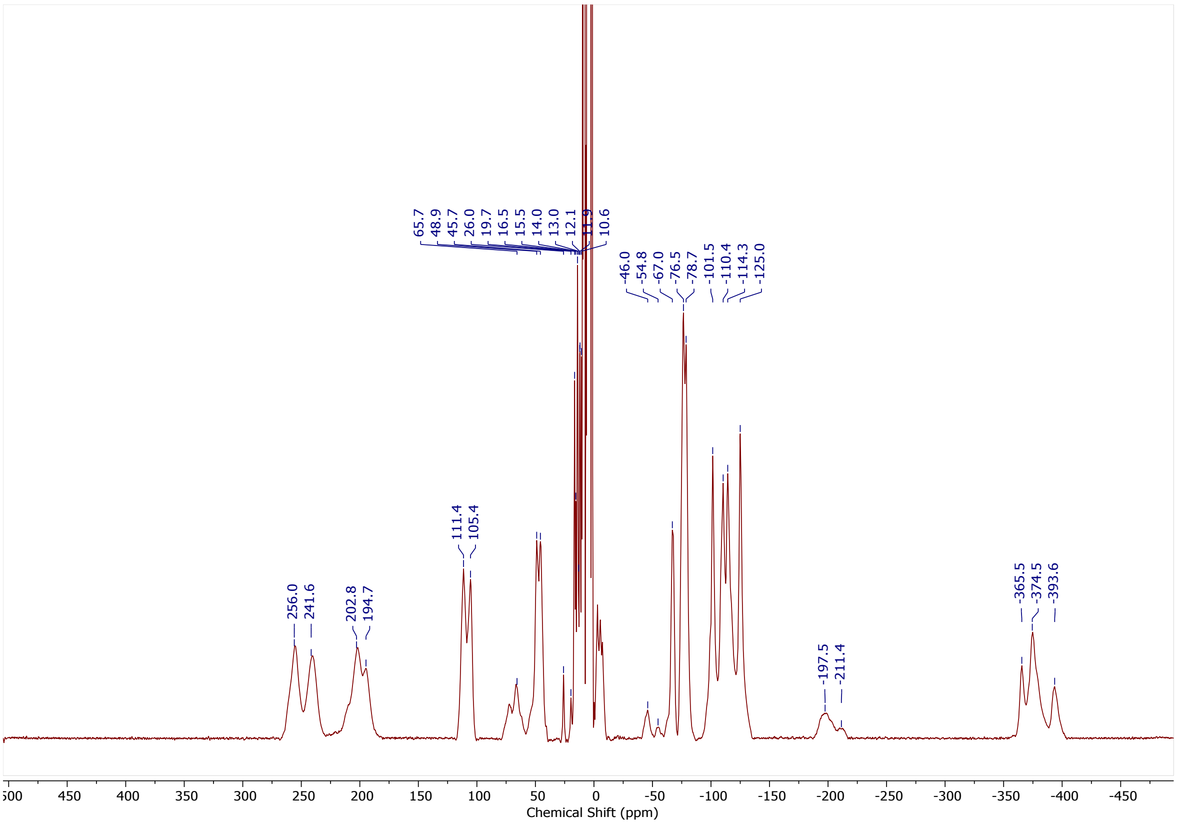

*Figure S32. ^{1}H NMR (500 MHz, D_2O) spectrum of **TbL[5]**, Tb-DOTA-DBCO clicked to FA-PEG$_5$-N_3 (mixture of regioisomers).*

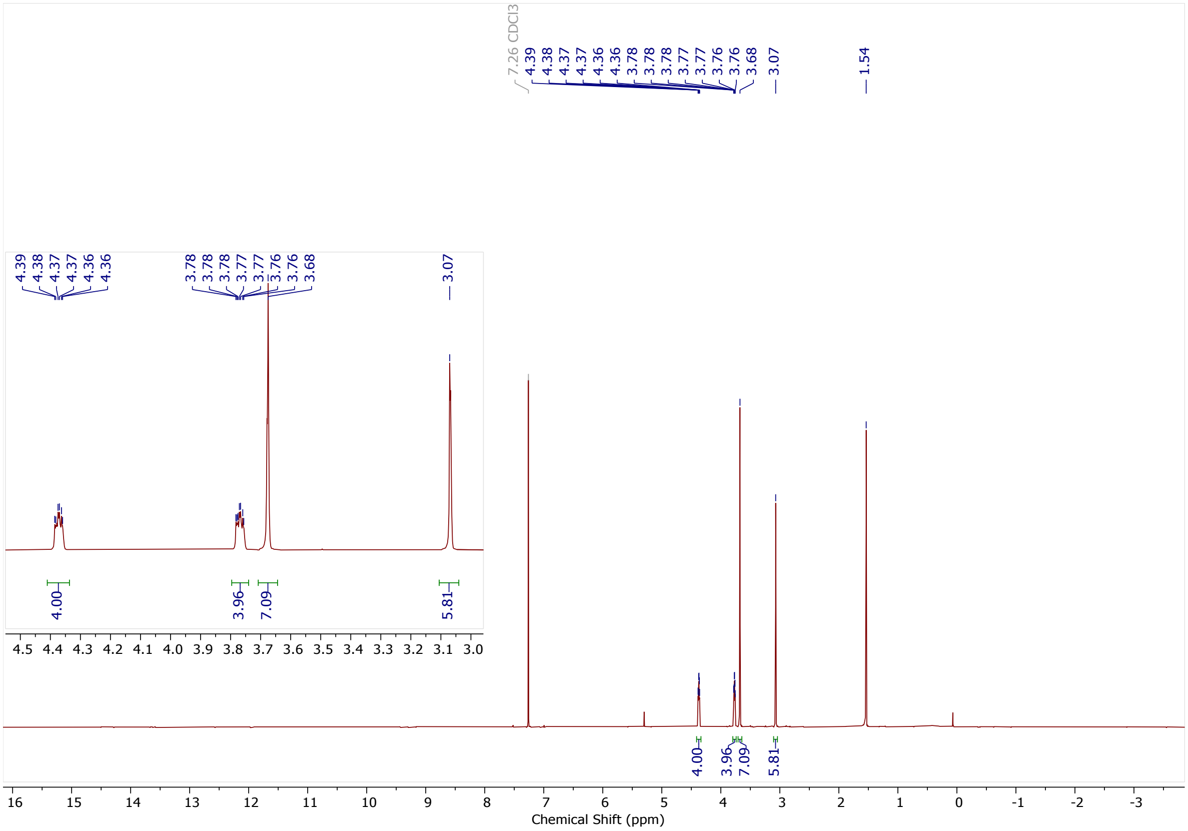

*Figure S33. ^{1}H NMR (400 MHz, $CDCl_3$) spectrum of **S4a**, dimesylate-PEG$_2$. Trace DCM at 5.3 ppm and water at 1.54 ppm.*

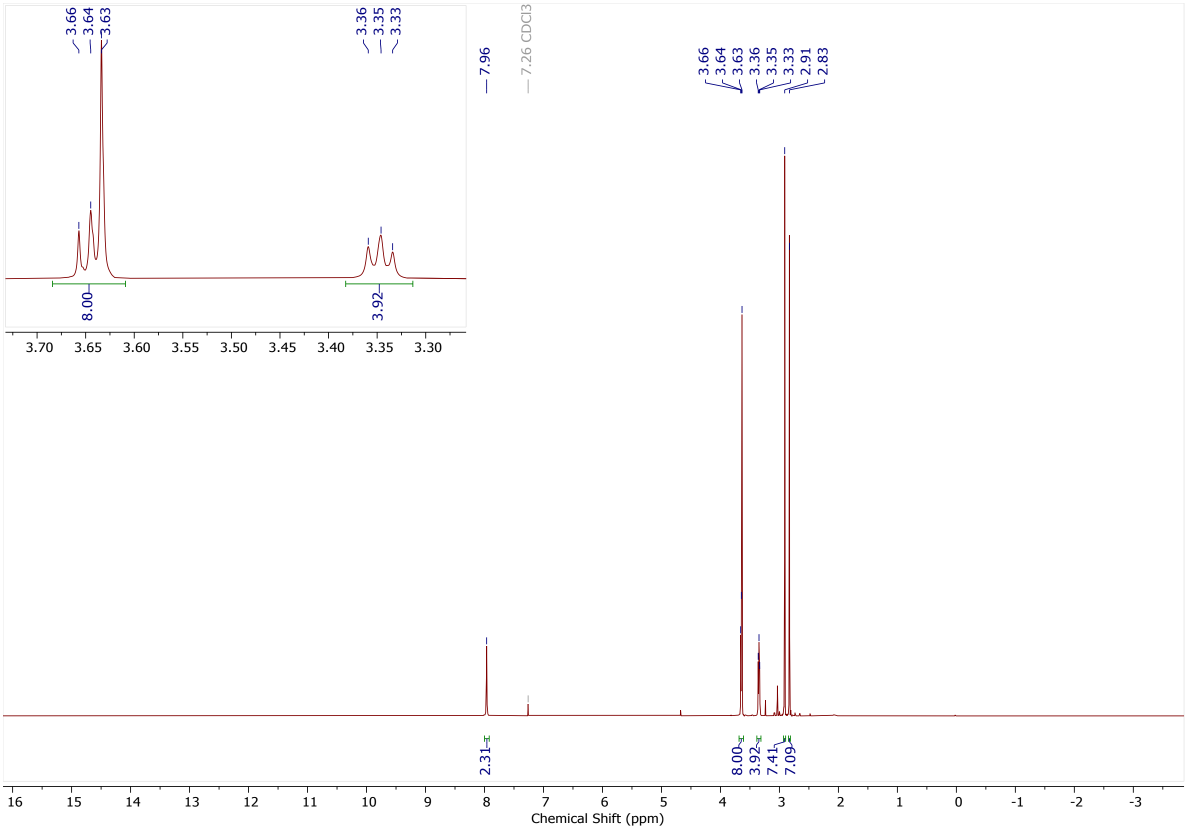

*Figure S34. ^{1}H NMR (400 MHz, $CDCl_3$) spectrum of **S5a**, 1,2-bis(2-azidoethoxy)ethane. DMF impurity at 7.96, 2.91 and 2.83 ppm.*

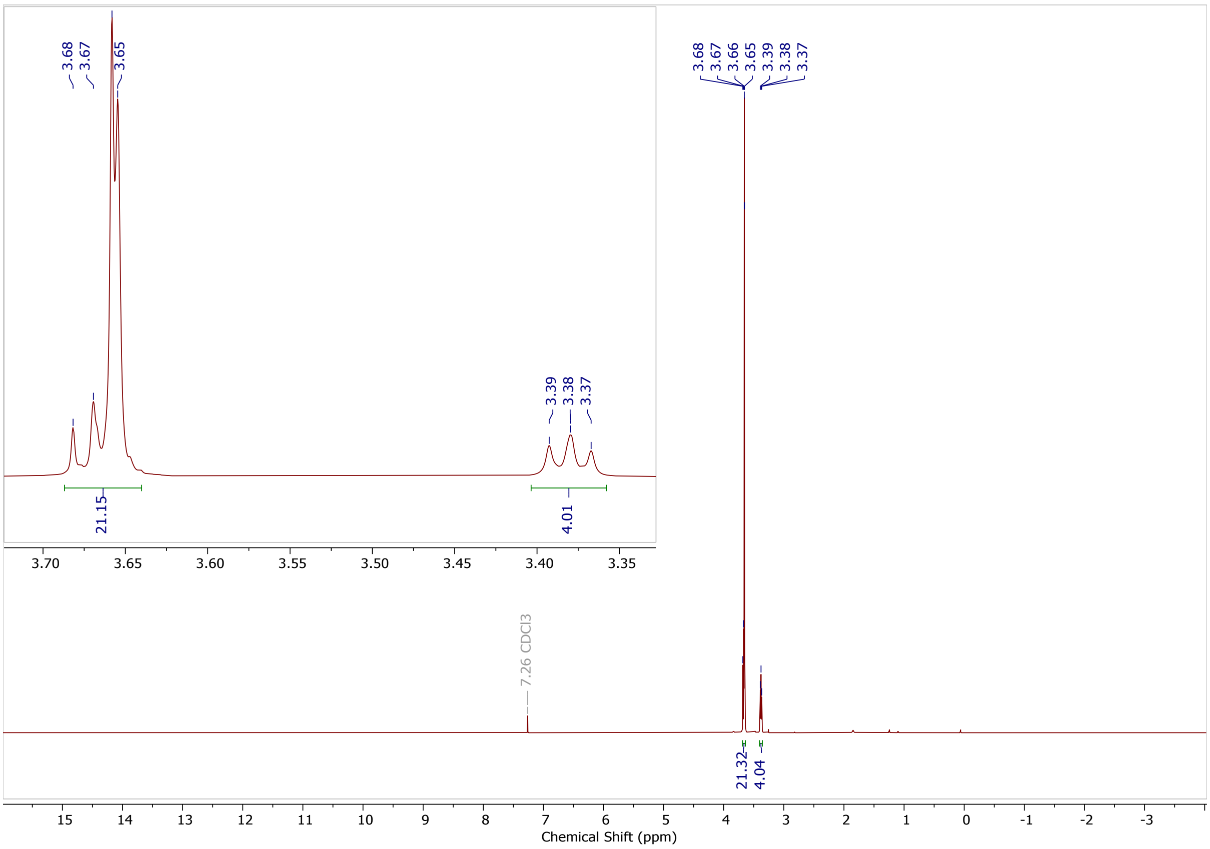

*Figure S35. ^{1}H NMR (400 MHz, $CDCl_3$) spectrum of **S5b**, 1,17-diazido-3,6,9,12,15-pentaoxaheptadecane.*

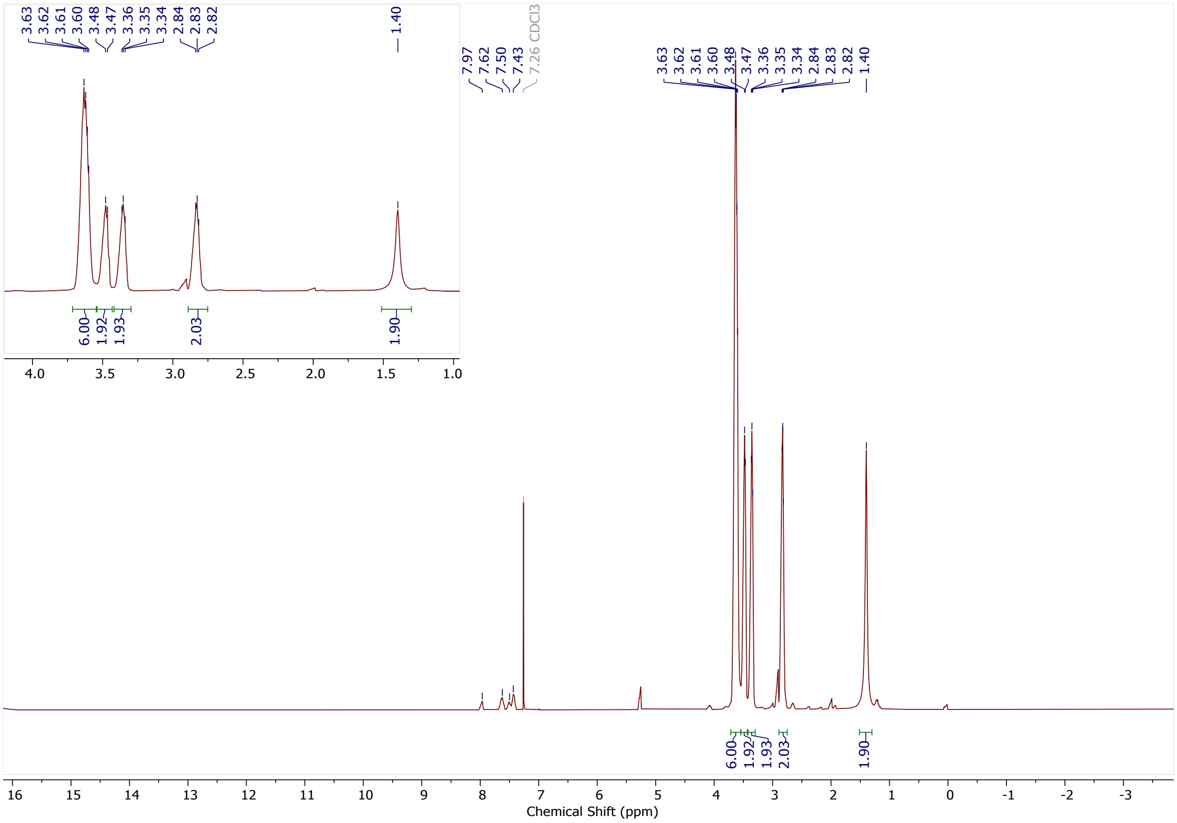

*Figure S36. ^{1}H NMR (400 MHz, $CDCl_3$) spectrum of **5a**, azide-PEG$_2$-amine. Contains triphenylphosphine or triphenylphosphine oxide impurity.*

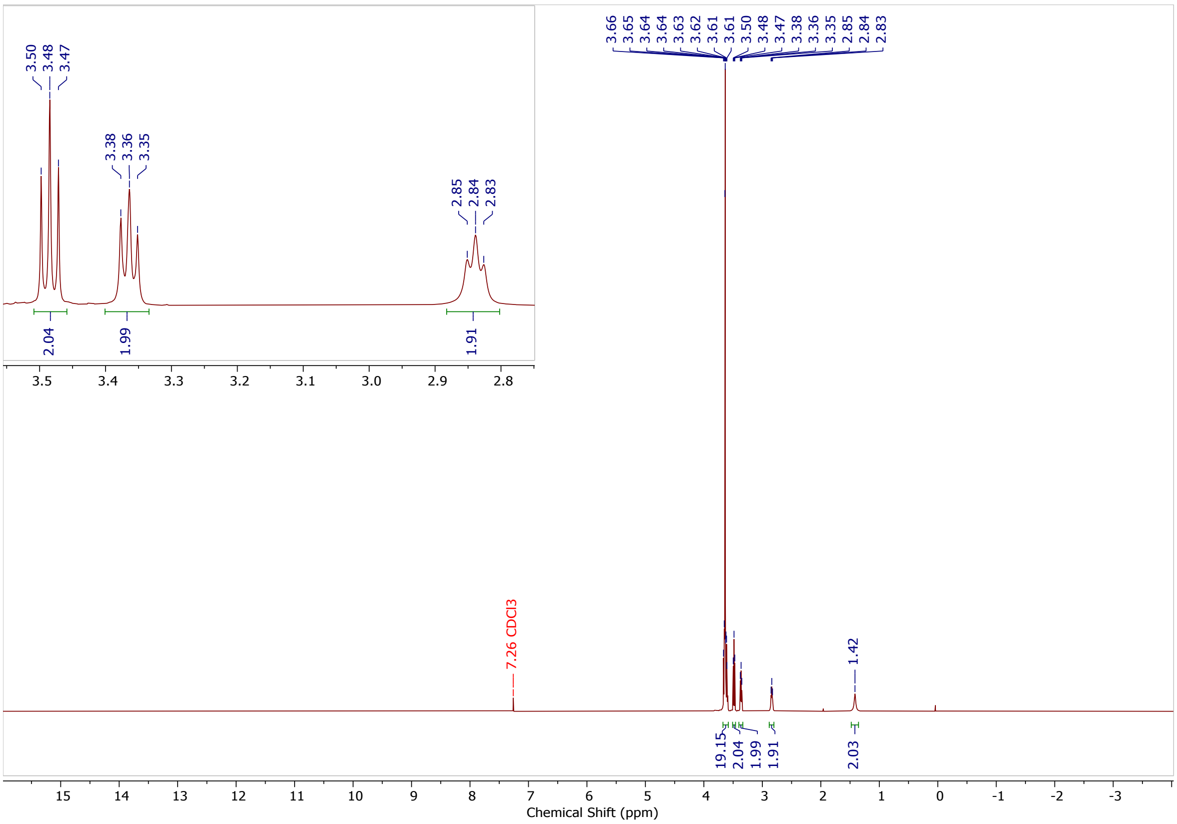

*Figure S37. ^{1}H NMR (400 MHz, $CDCl_3$) spectrum of **5b**, azide-PEG$_5$-amine.*

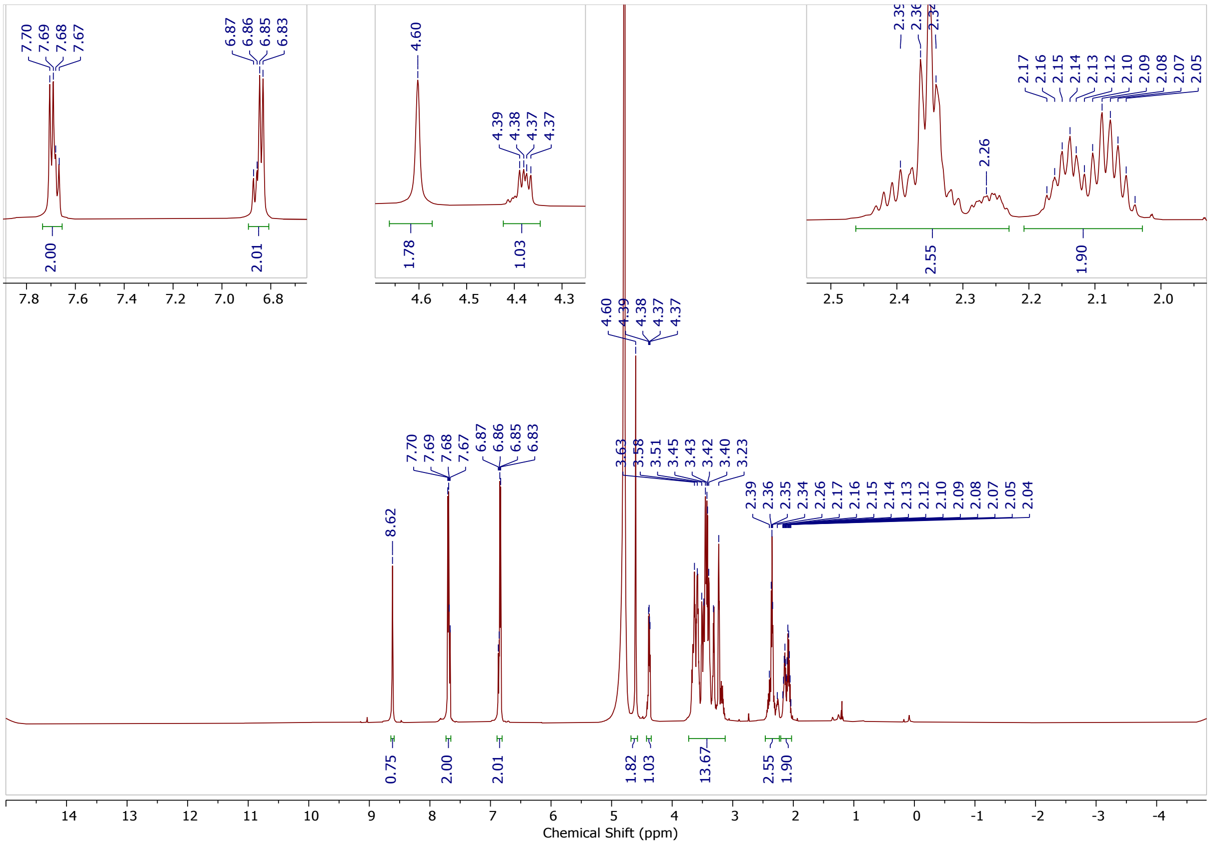

Figure S38. [1]*H NMR (600 MHz,* D_2O*, pH 9) spectrum of* ***6a****, Folate-PEG*$_2$*-azide, mixture of regioisomers*

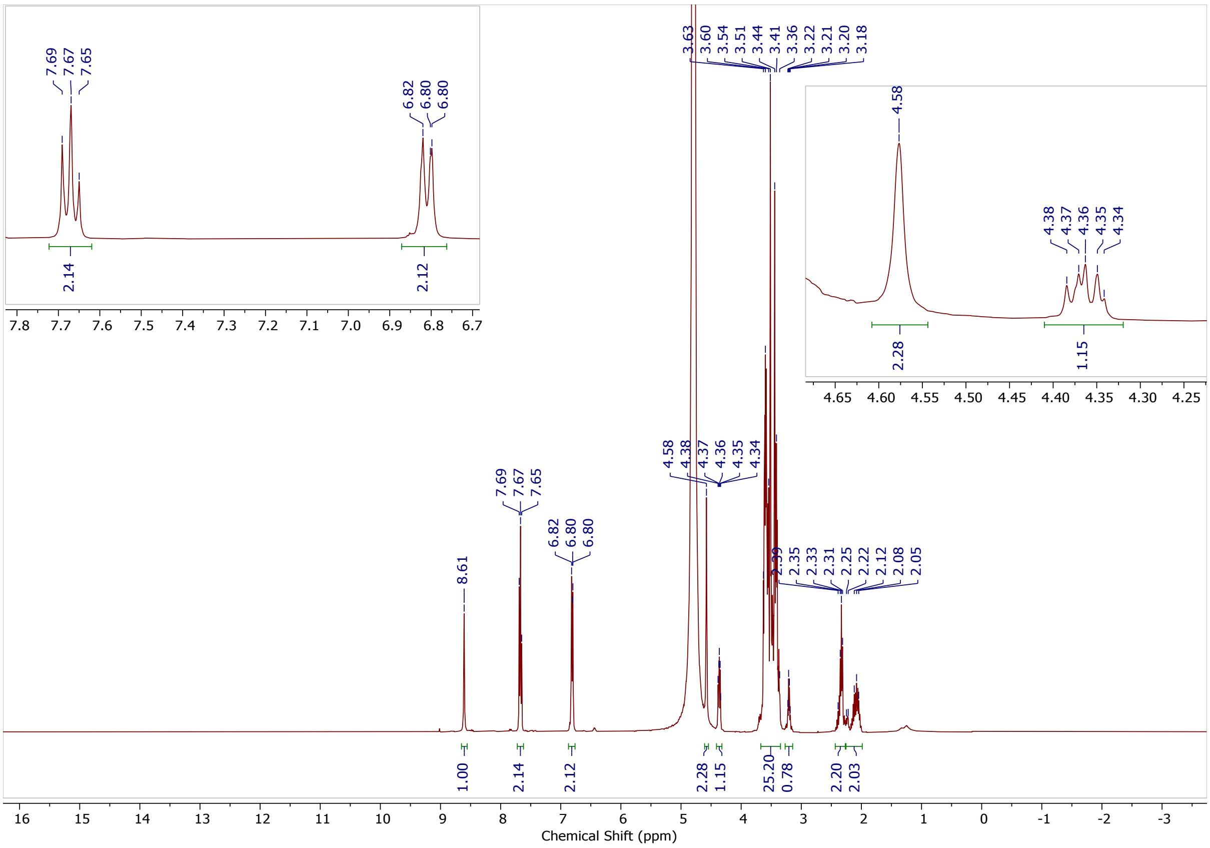

Figure S39. [1]*H NMR (400 MHz,* D_2O *with* NH_4OH*, pH 9) spectrum of* ***6b****, Folate-PEG*$_5$*-azide, mixture of regioisomers.*

6. *In silico* assessment of permeabilization properties

Table S1. In silico assessment of permeabilization properties of the compounds with regards to Lipinski's rule of five and Jorgensen's rule of three.

Ligand	#stars	Lipinski's rule of five						Jorgensen's rule of three		
		MW	DHB	AHB	QPlogPo/w	#rotor	PSA	QplogS	QPPCaco	#metab
**1**	**2**	**3**	**4**	**5**	**6**	**7**	**8**	**9**	**10**	**11**
**min**	**0**	**130.0**	**0.0**	**2.0**	**-2.0**	**0**	**7.0**	**-6.5**	**< 25 poor**	**1.0**
**max**	**5**	**725.0**	**6.0**	**20.0**	**6.5**	**15**	**200.0**	**0.5**	**> 500 great**	**8.0**
**Folic acid (ChEBI_27470)**	**150**	**441**	**5**	**9**	**-1.9**	**3**	**150**	**-0.9**	**3.8**	**1**
					**Ligand with DOTA**					
**L1α**	No data									
**L1γ**	20	1205	11	42	-7.4	42	485	2.0	0.0	21
**L2γ**	19	982	9	33	-5.2	27	429	1.4	0.0	16
**L3α**	18	1114	9	38	-5.2	36	449	2.0	0.0	19
**L3γ**	18	1114	9	38	-5.1	36	461	-0.4	0.0	19
**L4α**	19	1218	9	40	-3.9	37	476	-2.0	0.0	19
**L4γ**	19	1218	9	40	-3.9	37	486	-3.3	0.0	19
**L5α**	19	1392	9	42	-3.8	39	508	0.8	0.0	22
**L5γ**	18	1392	9	42	-3.5	39	506	0.2	0.0	22
					**Ligand without DOTA**					
**L1α**	18	861	8	28	-1.6	34	360	-3.2	0.0	13
**L1γ**	17	861	8	28	-1.4	34	357	-3.9	0.0	13
**L2α**	11	638	6	19	-0.2	19	287	-5.2	0.1	9
**L2γ**	10	638	6	19	-0.2	19	279	-4.7	0.1	9
**L3α**	15	770	6	24	0.5	28	303	-5.8	0.1	12
**L3γ**	15	770	6	24	0.2	28	303	-3.3	0.2	12
**L4α**	15	874	6	26	1.2	29	327	-5.1	0.2	11
**L4γ**	16	874	6	26	1.0	29	328	-5.9	0.1	11
**L5α**	17	1048	6	28	2.3	31	361	-7.3	0.1	14
**L5γ**	16	1048	6	28	2.3	31	357	-7.3	0.0	14

#Stars – number of property or descriptor values that fall outside the 95% range of similar values for known drugs. A large number of stars suggests that a molecule is less drug-like than molecules with few stars. The following properties and descriptors are included in the determination of #stars: MW, dipole, IP, EA, SASA, FOSA, FISA, PISA, WPSA, PSA, volume.

#rotor, donorHB, accptHB, glob, QPpolrz, QPlogPC16, QPlogPoct, QPlogPw, QPlogPo/w, logS, QPLogKhsa, QPlogBB, #metabol (see Table 2).

MW – molecular weight of the molecule, Dalton.

DHB and **AHB** – estimated number of hydrogen bonds that would be donated and would be accepted by the solute to water molecules in an aqueous solution. Values are averages taken over a number of configurations so that they can be non-integer. **QPlogPo/w** – predicted octanol/water partition coefficient.

#rotor – number of non-trivial (not CX3), non-hindered (not alkene, amide, small ring) rotatable bonds.

PSA – Van der Waals surface area of polar nitrogen, oxygen, and carbonyl carbon atoms.

QplogS – conformation-independent predicted aqueous solubility, log S. S in mol dm^{-3} is the concentration of the solute in a saturated solution that is in equilibrium with the crystalline solid.

QPPCaco – Predicted apparent Caco-2 cell permeability in nm/sec. Caco-2 cells are a model for the gut-blood barrier. These predictions are for non-active transport.

#metab – number of likely metabolic reactions.

7. Folate-FITC competition assay

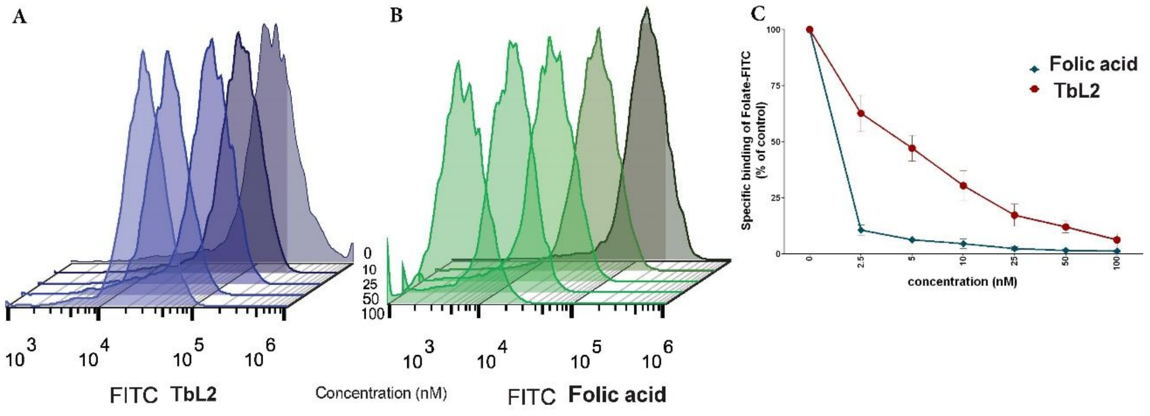

Figure S40. Folate-FITC competition assay. ***A***. *FITC histogram for Tb**L**^{2}.* ***B***. *FITC histogram for Folic acid.* ***C***. *comparison of different concentration of Folic acid and Tb**L**^{2} with regards to folate-FITC competitive binding.*

8. References

(1) Supkowski, R. M.; Horrocks, W. D. On the determination of the number of water molecules, *q*, coordinated to europium(III) ions in solution from luminescence decay lifetimes. *Inorganica Chimica Acta* **2002**, *340*, 44-48. DOI: 10.1016/s0020-1693(02)01022-8.

(2) Beeby, A.; Clarkson, I. M.; Dickins, R. S.; Faulkner, S.; Parker, D.; Royle, L.; de Sousa, A. S.; Williams, J. A. G.; Woods, M. Non-radiative deactivation of the excited states of europium, terbium and ytterbium complexes by proximate energy-matched OH, NH and CH oscillators: an improved luminescence method for establishing solution hydration states. *Journal of the Chemical Society-Perkin Transactions 2* **1999**, (3), 493-503. DOI: 10.1039/a808692c.

(3) Gelernt, B.; Findeisen, A.; Stein, A.; Poole, J. A. Absolute measurement of the quantum yield of quinine bisulphate. *Journal of the Chemical Society, Faraday Transactions 2* **1974**, *70*, 939. DOI: 10.1039/f29747000939.
